# Long non-coding RNA linc00921 suppresses tumorigenesis and epithelial-to-mesenchymal transition of triple-negative breast cancer via targeting miR-9-5p/LZTS2 axis

**DOI:** 10.1007/s13577-022-00685-6

**Published:** 2022-02-18

**Authors:** Jie Zhang, Lina Zhang, Jianlong Wang, Jing Zhao, Xuelian Zhao, Chunli Zhang, Peng Han, Cuizhi Geng

**Affiliations:** 1 Department of Plastic Surgery, Second Affiliated Hospital of Hebei Medical University, Shijiazhuang, 050000 Hebei China; 2 Breast Disease Diagnostic and Therapeutic Center, Fourth Affiliated Hospital of Hebei Medical University, Shijiazhuang, 050035 Hebei China; 3 Department of Minimally Invasive Surgery, Second Affiliated Hospital of Hebei Medical University, Shijiazhuang, 050000 Hebei China; 4 Department of Anus and Intestine Surgery, Second Affiliated Hospital of Hebei Medical University, ShijiazhuangHebei, 050000 China

**Keywords:** Triple-negative breast cancer, linc00921, LZTS2, miR-9-5p, EMT

## Abstract

Triple-negative breast cancer (TNBC) is the most aggressive subtype of breast cancer. Dysregulation of long non-coding RNAs (lncRNAs) plays crucial roles in the initiation and progression of TNBC. In this study, we analyzed public GEO profiles to verify the key lncRNAs in TNBC. Linc00921 was selected for further study. Low expression of linc00921 was observed in 49 of 95 TNBC tissues. Low expression of linc00921 was correlated with poor postoperative disease-free survival (DFS) and overall survival (OS) of TNBC patients. Overexpression of linc00921 with lentivirus suppressed the proliferation, migration and invasion of TNBC cells. A luciferase reporter assay showed that linc00921 could sponge miR-9-5p in TNBC. Moreover, linc00921 and miR-9-5p occupied the same Argonaute-2 (Ago2) protein in TNBC cells. Leucine zipper tumor suppressor 2 (LZTS2) was recognized as a target gene of miR-9-5p, and thereby a linc00921/miR-9-5p/LZTS2 axis was identified in TNBC cells. Overexpression of linc00921 promoted nuclear export of β-catenin, neutralized its function, and subsequently promoted epithelial-to-mesenchymal transition (EMT) in TNBC. A xenograft tumor mouse model showed that the miR-9-5p inhibitor upregulates LZTS2 expression and induce nuclear export of β-catenin in TNBC. Thus, linc00921 upregulates LZTS2 by sponging miR-9-5p to suppress tumorigenesis and EMT of TNBC. Linc00921/miR-9-5p/LZTS2 axis may be a novel biomarker and therapeutic target for TNBC patients.

**Supplementary Information:**

The online version contains supplementary material available at 10.1007/s13577-022-00685-6.

## Introduction

Breast cancer ranks first in female malignant tumor, threatening women’s health worldwide [1]. Breast cancers are classified into four subtypes: Luminal A, Luminal B, HER2 (human epidermal growth factor receptor 2) overexpression and triple-negative (also known as basal-like), according to the expression of estrogen receptor (ER), progesterone receptor (PR), Ki-67 and HER-2. Among these 4 subtypes, triple-negative breast cancer (TNBC) still has the poorest prognosis due to its formidable aggressiveness and high rate of recurrence [2]. Despite the improvement in treating approaches including surgery, chemotherapy, radiotherapy and immunotherapy, the overall survival of TNBC patients remains unsatisfactory. Dysregulation of genes, especially loss or attenuation of tumor-suppressing genes, plays crucial roles in the initiation and progression of TNBC [3]. Thus, further elucidation of the mechanisms of aberrant expression of tumor suppressor genes is necessary for establishing novel treatment strategies to improve the prognosis of TNBC patients.

The Encyclopedia of DNA Elements (ENCODE) project has confirmed that most human transcripts are non-coding RNAs including microRNAs (miRNAs) and long non-coding RNAs (lncRNAs) [4]. MiRNAs are evolutionarily conserved single-stranded RNAs that consist of approximately 21–24 nucleotides [5]. MiRNAs can bind to the 3′-untranslated regions (UTRs) of target mRNAs to exert regulatory functions by mediating their degradation or translational inhibition [6]. Studies have confirmed that dysregulation of miRNAs is involved in tumorigenesis, metastasis and treatment resistance [7]. As another important member of non-coding RNAs, lncRNAs are a group of transcripts longer than 200 nucleotides, with limited or no protein-coding capacity [8]. Emerging studies have demonstrated that lncRNAs can post-transcriptionally regulate gene expression by acting as competitive endogenous RNAs (ceRNAs) which sponge miRNAs to neutralize their functions [9]. Thus, lncRNAs function as either tumor-promoting genes or tumor-suppressing genes, due to their specific target genes in cancers [10]. For instance, lncRNAs H19, ANRIL, NORAD, ROR, XIST, NEAT1 and MALAT1 function as oncogene, while some lncRNAs, including MEG3, NCRNACCND1 and CASC15-S, function as tumor suppressors [11]. Accumulating studies have confirmed that dysregulation of lncRNAs is also a key factor contributing to the progression of TNBC [7]. However, the precise clinical significance and biological functions of most lncRNAs in TNBC are still not clear.

In the present study, we analyzed two public GEO profiles and showed that linc00921 is an underlying tumor-suppressing lncRNA in TNBC. Low expression of linc00921 was significantly correlated with poor prognosis of TNBC patients. Overexpressing linc00921 inhibited the proliferation, migration and invasion of TNBC cells. Furthermore, we verified a linc00921/miR-9-5p/leucine zipper tumor suppressor 2 (LZTS2) axis in TNBC. The linc00921/miR-9-5p/LZTS2 axis suppressed the progression of TNBC by promoting the nuclear export of β-catenin. Therefore, this study provided evidence that linc00921 might be a novel diagnostic indicator and therapeutic target for treating TNBC.

## Materials and methods

### Patients and specimens

TNBC tissues and matched para-carcinoma tissues were collected from 95 patients who underwent radical operation at the Fourth Affiliated Hospital of Hebei Medical University (Shijiazhuang, China) between July 2016 and January 2017. The median patient age at the time of surgery was 50 years (range 29–74 years). None of the TNBC patients received preoperative radiotherapy, chemotherapy or immunotherapy. The clinical stage and histological tumor type were determined according to the International Union Against Cancer (UICC) Classification of 2009 (seventh edition). Patient clinical information including age, TNM stage, invasion range, and lymph node metastasis, was collected and stored in a database. All participant information was updated every 3 months by telephone follow-up. Complete follow-up was updated until death or July 2021. Carcinoma tissue and para-carcinoma tissue specimens were collected and treated promptly after surgery. This research was approved by the Ethics Committee of Fourth Affiliated Hospital of Hebei Medical University and was carried out according to the guidelines of Declaration of Helsinki. All patients involved in this study provided informed consent.

### Bioinformatic analysis

The microarray data profiles (GSE119233 and GSE115275) were downloaded from the Gene Expression Omnibus (GEO) database (http://www.ncbi.nlm.nih.gov/geo/). Limma package in R was used to screen differentially expressed lncRNAs. Venn diagram were made by using VennDiagram in R to present the overlapping lncRNAs. RNA-sequencing (RNA-seq) data for a BRCA cohort were downloaded from The Cancer Genome Atlas (TCGA) (http://cancergenome.nih.gov/). The expression of linc00921 and LZTS2 is presented as fragments per kilobase million (FPKM). The TNBC patients were screened out according to ER, PR and HER2 expression status. The RNA-seq data were analyzed by using the DESeq2 package in R.

### Cell culture

Human TNBC cell lines (MDA-MB-468, HCC-1937, MDA-MB-231, MDA-MB-436, MDA-MB-453) and a human normal mammary epithelial cell line (MCF-10A) were obtained from the Research Center of Fourth Affiliated Hospital of Hebei Medical University (Shijiazhuang, China). All employed cell lines were routinely cultured in DMEM (Thermo Fisher Scientific, Waltham, MA, USA) containing 10% fetal bovine serum (FBS) (Gibco, New York, NY, USA) with 1% Pen/Strep and 1% non-essential amino acids (Solarbio, Beijing, China), at 37 °C in a humidified atmosphere in a 5% CO_2_ incubator.

### Western blotting

For western blotting analysis, proteins were extracted by using RIPA lysis buffer (Beyotime, Shanghai, China). Proteins were added to 4–20% gels, subjected to 160 V to promote migration, and then transferred onto PVDF membranes (Millipore, Billerica, MA, USA). The membranes were blocked with 5% BSA for 1 h and incubated at 4 °C overnight with primary antibodies as follows: anti-LZTS2 (sc-514618, Santa Cruz Biotechnology, Dallas, Texas, USA), anti-β-catenin (GB11015, Servicebio, Wuhan, China), anti-phosphor-β-catenin (#4176, Cell Signaling Technology, Danvers, MA, USA), anti-E-cadherin (GB81868, Servicebio), anti-N-cadherin (66219-1-Ig, Proteintech, Rosemont, IL, USA), anti-Histone H3 (ab176842, Abcam, Cambridge, UK) and anti-GAPDH (GB11002, Servicebio). Then, the species-matched secondary antibodies were incubated for 2 h at room temperature. Then protein bands were developed by an ECL chemiluminescent substrate kit (Biosharp Life Sciences, Hefei, China). Band intensities were quantified using ImageQuant LAS 500 (GE, Boston, MA, USA). For evaluation of the expression of β-catenin located in the cytoplasm and nucleus, GAPDH and histone H3 were used as internal controls, respectively.

### Immunohistochemistry (IHC)

IHC was performed by using the streptavidin-peroxidase (SP) method. Briefly, sections (5-μm thick) were pretreated using sodium citrate buffer (pH 6.0; 0.01 mol/l; Solarbio) at 98 °C for 5 min to retrieve cell antigens and then blocked with goat serum at room temperature for 20 min. After incubation with antibody to LZTS2 at a dilution of 1:100 overnight at 4 °C, the sections were biotinylated secondary antibody and streptavidin-biotinylated horseradish peroxidase complex (Zsbio, Beijing, China). Diaminobenzidine (DAB, Zsbio) was used as a color developer. In each experiment, negative control sections were treated similarly with phosphate-buffered saline (PBS) instead of the primary antibody. IHC staining was evaluated by using IHC Profiler in Image J. Scores 0 and 1+ were regarded as negative expression and scores of 2+ and 3+ were regarded as positive expression.

### Reverse transcription quantitative PCR (RT-qPCR)

Total RNA from tissues and cells was extracted with TRIzol reagent (Invitrogen, Carlsbad, CA, USA), and cDNA was synthesized according to the manufacturer’s instructions by using a RevertAid™ First Strand cDNA Synthesis Kit (Thermo Fisher Scientific, Waltham, MA, USA). RT-qPCR analysis was performed using an ABI Prism 7900-HT Sequence Detection System (96-well, Applied Biosystems). Primers were designed and synthesized by RiboBio (Guangzhou, China). The primers used in RT-qPCR are listed in Supplementary Tables 1 and 2. The relative expression of lncRNAs was normalized to that of GAPDH, and miRNAs were normalized to U6.

### Plasmid transfection

A eukaryotic expression plasmid of the human linc00921 gene was constructed using a pCDH-CMV-MCS-EF1-copGFP-T2A-Puro vector (Huayueyang, Beijing, China), referred to as pCDH-linc00921. The full-length linc00921 was used to establish a vector. The empty vector (EV) was used as negative control. HCC-1937 and MDA-MB-231 cells were cultured in six-well plates. When cells reached 80–90% confluence, transient transfections were performed by using Lipofectamine 2000 (Invitrogen) according to the manufacturer’s instructions. At 48 h after transfection, gene expression was confirmed by using Western blotting analysis or RT-qPCR.

### Cell counting kit-8 (CCK-8) assay

Cell proliferation was measured by conducting a CCK-8 assay according to the manufacturer's protocol. Briefly, approximately 2 × 10^3^ cells were seeded in a 96-well plate and incubated overnight. When cells adhered well, 10 μl of CCK-8 solution (Solarbio) was added to each well and incubated for 2 h at 37 °C. A microplate reader spectrophotometer was used to measure the optical density (OD) at 450 nm. Proliferation rates were determined at 0, 24, 48, 72, and 96 h after transfection. Experiments were performed in triplicate.

### Wound-healing experiments

First, 5 × 10^5^ TNBC cells were seeded in 6-well plates. After the cell monolayer was scraped with a sterile micropipette tip, the wells were washed with serum-free medium three times. The first image of each scratch was acquired at time zero. After 48 h, each scratch was examined and captured. Then the healed area was measured to evaluate the migration index. The migration index = (scratch at 0 h − scratch at 48 h)/scratch at 0 h.

### Transwell assay

The tumor cell migration assay was performed in a 24-well Transwell chamber (Corning, NY, US), which contained an 8 μm pore size polycarbonate membrane filter and was precoated with 100 μg Matrigel for the invasion assay (Becton-Dickinson, Bedford, USA). Briefly, the cells were seeded in the upper chambers and incubated in 500 μl of DMEM medium without FBS, while 500 μl of medium with 10% FBS was placed in the lower chambers. The plates were incubated for 24 h in a 5% CO_2_ humidified incubator at 37 °C. Cells on the upper side of the filters were removed by cotton-tipped swabs, and the filters were washed with PBS. Then the cells on the lower side were fixed in 4% formaldehyde and stained with 1% crystal violet in PBS for 5 min at room temperature. The cells on the lower side of the filters were defined as migrating cells and counted at 200 × magnification in 5 random fields of each filter.

### Fluorescence in situ hybridization (FISH)

The FISH assay in TNBC cells was performed as previously described [12]. FISH probes were designed and synthesized by Servicebio. A biotin-labelled probe specific to linc00921 was used in hybridization. Probe sequence: 5′-TCCTTGGAACAGCGATCTTGCCTG-3′. The samples were counterstained with 6-diamidino-2-phenylindole (DAPI) and observed by confocal microscopy.

### Dual-luciferase reporter assay

Plasmids containing the firefly luciferase reporter were constructed with wild-type LZTS2-3′-UTR (LZTS2-3′-UTR-WT) and mutant LZTS2-3′-UTR (LZTS2-3′-UTR-Mut). Approximately 5 × 10^4^ cells were seeded in 24-well plates and allowed to settle overnight. The next day, the cells were transfected with recombinant plasmids or an empty plasmid encoding the firefly luciferase reporter with Lipofectamine 2000. The Renilla luciferase reporter pRL-CMV (Promega) was used as an internal control and used for normalization. The mutant sequences of linc00921 and the LZTS2-3′-UTR were created by using a Mut Express II Fast Mutagenesis Kit (Vazyme, Nanjing, China). After 48 h, the reporter luciferase activity was measured with the Dual-luciferase Reporter assay system (Promega) according to the manufacturer’s instructions. All transfection assays were carried out in triplicate.

### RNA immunoprecipitation (RIP)

RIP experiments were conducted using the BersinBio™ RIP Kit (BersinBio, Guangzhou, China) according to the manufacturer’s instructions. Briefly, TNBC cells were collected, and RIP lysate was added to obtain cell lysates. Anti-Ago2 antibody (ab186733, Abcam) or IgG (ab172730, Abcam) complex was prepared for immunoprecipitation. Then, the cell supernatants were incubated with magnetic bead-antibody complexes for 2 h at 4 °C. Then, RNA was purified, and the obtained RNA was used to detect the expression of linc00921 and miR-9-5p by RT-qPCR.

### Isolation of nuclear and cytoplasmic fractions

Cytosolic and nuclear fractions of HCC-1937 and MDA-MB-231 cells were collected by using the Nuclear and Cytoplasmic Protein Extraction Kit (Beyotime). The detailed experimental procedures were performed according to the manufacturer’s instructions.

### In vivo tumor growth assay

Ten female BALB/c nude mice (4 weeks old) were used to establish a xenograft tumor model. The nude mice were randomly divided into 2 groups for injecting different TNBC cells. Approximately 5 × 10^6^ HCC-1937 or HCC-1937^linc00921-KD^ cells were subcutaneously injected into BALB/c nude mice. Tumor growth was recorded by measuring the width (W) and length (L). Tumor volume was calculated at the indicated times by using the formula: 0.5 × L × W^2^. These mice were sacrificed and the tumor tissues were harvested on the 30th day. The experiments involving animals were approved by the Ethics Committee for the Use and Care of Animals of Hebei Medical University.

### Statistical analysis

All statistical analyses were performed with SPSS statistics software, version 25.0 (SPSS, Chicago, IL, USA). The graphs were made by using GraphPad Prism 9.2. Data are presented as the mean ± SD. All P values were two-tailed and a *P* value < 0.05 was considered statistically significant. Data were obtained from at least three independent experiments with a similar pattern.

## Results

### Low expression of linc00921 in tumor tissues was correlated with poor postoperative prognosis of TNBC patients

**Fig. 1:**
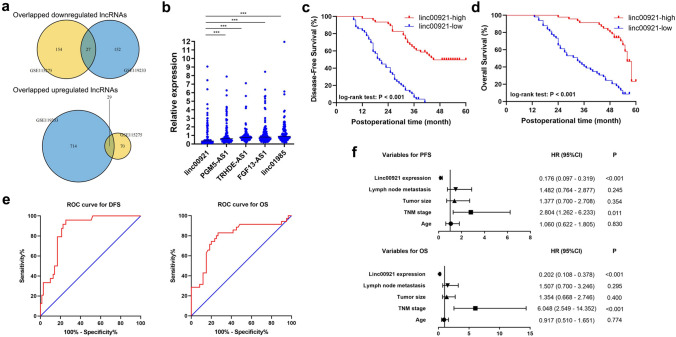
Linc00921 expressed at low levels in TNBC tissues and positively correlated with the postoperative prognosis of patients. **a** Schematic illustrations showing the overlapping lncRNAs in GSE119233 and GSE115275. **b** The expression of the top 5 overlapping lncRNAs which were downregulated in TNBC specimens, detected by RT-qPCR (*N* = 3). **c**, **d** TNBC patients with high expression of linc00921 had longer PFS and OS than those with low expression. **e** ROC curves of linc00921 for predicting DFS and OS of TNBC patients. **f** Variables predicting PFS and OS of TNBC patients, analyzed by Cox proportional hazards model. ***P < 0.001

To verify the potential dysregulated lncRNAs in TNBC, we analyzed two public GEO profiles GSE119233 and GSE115275, which contained the differentially expressed lncRNAs between TNBC and normal breast tissues. By setting the fold-change to 2 and the *P* value to 0.01, we identified 922 and 280 validated lncRNAs that were differentially expressed in GSE119233 and GSE115275, respectively (Supplementary Fig. 1 and Supplementary Table 3). By overlapping the results, we found that 27 lncRNAs had downregulated expression and 29 lncRNAs had upregulated expression in TNBC tissues compared to normal breast tissues (Fig. 1a and Supplementary Table 4). Then, we analyzed the top 5 lncRNAs with significantly downregulated expression in our collected TNBC specimens by RT-qPCR. The results showed that all these lncRNAs had downregulated expression in TNBC tissues, and linc00921 (NR_033904.1) exhibited the lowest expression (Fig. 1b). Furthermore, we analyzed the RNA-seq data of a TNBC cohort in the TCGA database. The expression of linc00921 was lower in the TNBC tissues than in normal breast tissues (*P* < 0.001, Fig. 1c). Therefore, we chose linc00921 for further study.

Then, we calculated the cut-off value of linc00921 expression to define its expression in TNBC specimens. As a result, the cut-off value of linc00921 expression was determined to be 0.55 by using X-tile [13]. Among the 95 TNBC tissues, 49 cases (51.58%) showed low expression of linc00921. Then, we analyzed the correlation between linc00921 and the clinical parameters of the TNBC patients. The results showed that low expression of linc00921 was significantly correlated with high TNM stage, large tumor size and positive lymph node metastasis but not with the age of the TNBC patients (Table 1). Next, to elucidate the predictive role of linc00921 in prognosis of TNBC patients, we analyzed its correlation with the post-operational disease-free survival (DFS) and overall survival (OS) of TNBC patients. Kaplan–Meier analysis indicated that low expression of linc00921 was correlated with shorter DFS (Fig. 1d) (log-rank test: *P* < 0.001). The median DFS for the patients with high and low linc00921 expression was 45.0 and 20.0 months, respectively. Moreover, Kaplan–Meier analysis demonstrated that low expression of linc00921 was correlated with shorter OS (Fig. 1d) (log-rank test: *P* < 0.001), and the median OS for the patients with high and low linc00921 expression was 56.0 and 33.0 months, respectively. These results indicated that low expression of linc00921 was significantly correlated with poor prognosis in TNBC patients. Next, we evaluated whether linc00921 could be an independent biomarker for predicting the prognosis of TNBC patients by drawing receiver operator characteristic (ROC) curves. The area under the ROC curve (AUC) was 86.59% for DFS (95% CI 79.31–93.87%) and 79.33% for OS (95% CI 69.27–89.40%) (both *P* < 0.001; Fig. 1e). The sensitivity of linc00921 for predicting DFS and OS was 0.746 and 0.733, and the specificity of linc00921 for predicting DFS and OS was 0.958 and 0.829. Then, we conducted Cox proportional hazards model to further assess the predictive role of linc00921 in the prognosis of TNBC patients. The covariates included in the Cox proportional hazards model were age, TNM stage, tumor size, lymph node metastasis and linc00921 expression. After stepwise multivariate survival analysis, TNM stage and linc00921 were found to be significantly correlated with the DFS and OS of the TNBC patients (Fig. 1f). In summary, low expression of linc00921 was significantly correlated with the prognosis of the TNBC patients, and linc00921 expression might be regarded as a potential independent predictive biomarker for TNBC patients.

**Table 1:** Correlations of linc00921 expression with clinical parameters of TNBC patients

	Total	*N* (%)		*P*
		High expression of linc00921	Low expression of linc00921	
Age				
< 60	71	36 (50.70%)	35 (49.30%)	0.486
≥ 60	24	10 (41.67%)	14 (58.33%)	
TNM stage				
I + II	68	42 (61.76%)	26 (38.24%)	< 0.001
III	27	4 (14.81%)	23 (85.19%)	
Tumor size				
T1 + T2	64	36 (56.25%)	28 (43.75%)	0.031
T3 + T4	31	10 (32.26%)	21 (67.74%)	
Lymph node metastasis				
Negative	32	22 (68.75%)	10 (31.25%)	0.009
Positive	63	24 (38.10%)	39 (61.90%))	

### Linc00921 overexpression suppresses the proliferation, migration, and invasion of TNBC cells

**Fig. 2:**
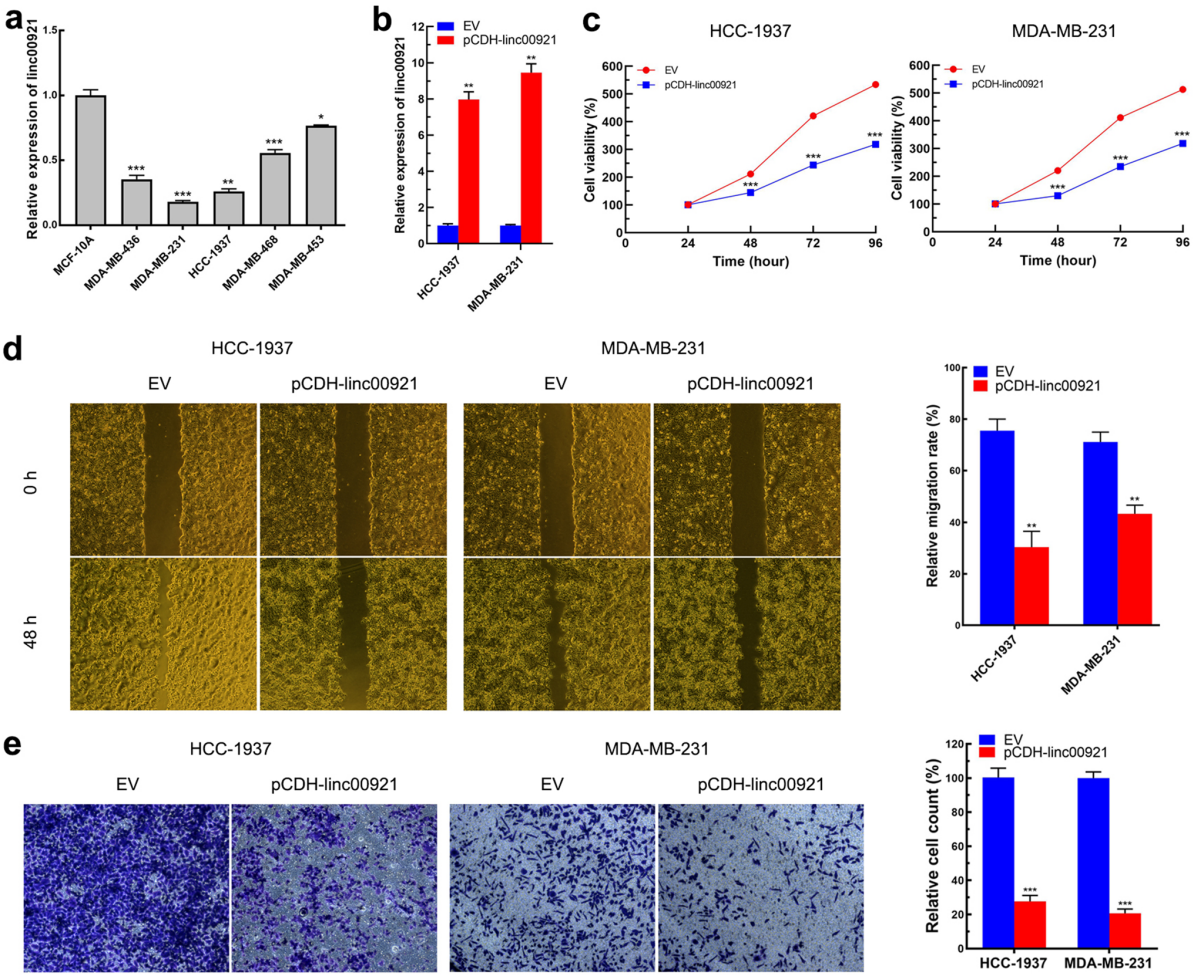
Overexpression of linc00921 inhibits the proliferation, migration and invasion of TNBC cells in vitro. **a** Expression of linc00921 in TNBC cell lines, assayed by RT-qPCR, MCF-10A cell were used as a control. (*N* = 3). **b** Plasmid packaging linc00921, referred to as pCDH-linc00921, increased the expression of linc00921 in TNBC cells. **c** Overexpression of linc00921 inhibited the proliferation of TNBC cells, assayed by CCK-8 (*N* = 3). **d** Overexpression of linc00921 inhibited the migration of TNBC cells, assayed by wound-healing experiment (*N* = 3). **e** Overexpression of linc00921 inhibited the invasion of TNBC cells, assayed by Transwell (*N* = 3). *P < 0.05, **P < 0.01, ***P < 0.001

To further study the function of linc00921 in TNBC, we used RT-qPCR to determine linc00921 expression in a human normal mammary epithelial cell line (MCF-10A) and TNBC cell lines (MDA-MB-468, HCC-1937, MDA-MB-231, MDA-MB-436, MDA-MB-453). The results demonstrated that the expression of linc00921 was lower in all chosen TNBC cell lines than in MCF-10A cells (Fig. 2a) (all *P* < 0.001). Among these TNBC cell lines, HCC-1937 and MDA-MB-231 cells showed the lowest expression of linc00921; thus, these two cell lines were chosen for subsequent studies. Then, we overexpressed linc00921 with plasmids in HCC-1937 and MDA-MB-231 cells to reveal the biological role of linc00921 in TNBC progression (Fig. 2b). First, we used a CCK-8 assay to evaluate the effect of linc00921 on the proliferation of HCC-1937 and MDA-MB-231 cells. The results showed that linc00921 overexpression significantly decreased the proliferation of HCC-1937 and MDA-MB-231 cells (Fig. 2c). Next, we assessed the effect of linc00921 overexpression on cell migration and invasion by conducting wound healing and Transwell experiments. As Fig. 2d shows, linc00921 overexpression significantly inhibited the migration of HCC-1937 and MDA-MB-231 cells, compared with the corresponding control cells (*P* < 0.001). Similarly, linc00921 overexpression also significantly inhibited the invasion of HCC-1937 and MDA-MB-231 cells, compared with the corresponding control cells (155.17 ± 2.95 cells/field vs*.* 76.64 ± 3.17 cells/field; 145.41 ± 2.97 cells/field vs. 72.89 ± 1.60 cells/field; *P* < 0.001; Fig. 2e). Taken above, overexpression of linc00921 inhibited the proliferation, colony formation, migration, and invasion ability of HCC-1937 and MDA-MB-231 cells, which indicated that LZTS2 might play important roles in managing the malignant behavior of TNBC cells.

### Linc00921 acts as a ceRNA to sponge miR-9-5p in TNBC cells

**Fig. 3:**
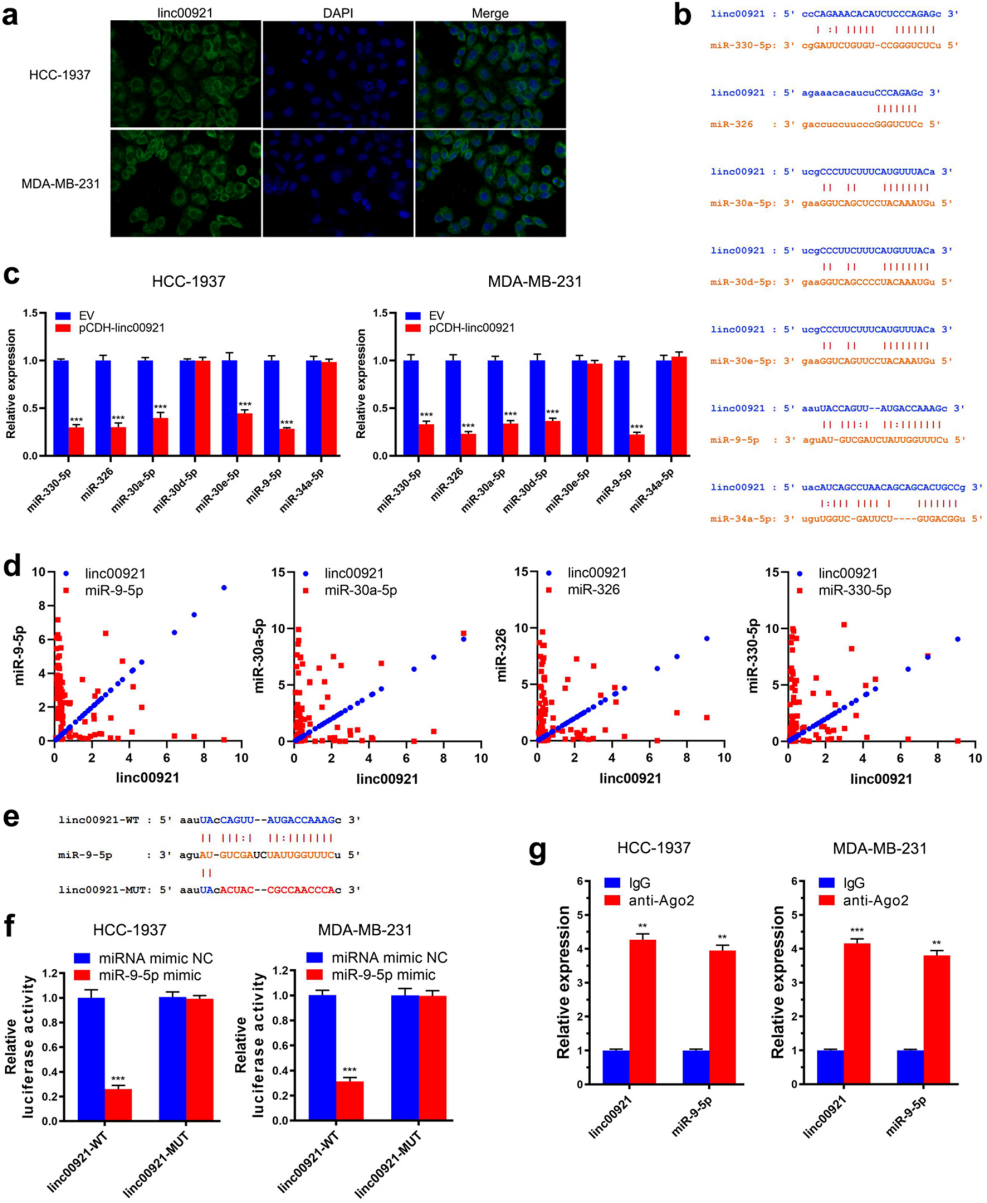
Linc00921 sponges miR-9-5p in TNBC. **a** Linc00921 was predominantly located in the cytoplasm of TNBC cells, detected by FISH. (*N* = 3). **b** Candidate miRNAs potentially sponged by linc00921, predicted by using bioinformatics algorithms. **c** Effects of overexpression of linc00921 on the expressions of candidate miRNAs in TNBC cells (*N* = 3). **d** Correlation between candidate miRNAs and linc00921 in TNBC tissues. **e** The sequences of linc00921-WT and linc00921-Mut used for luciferase reporter assays. **f** Luciferase activity of TNBC cells transfected with linc00921-WT was decreased by co-transfection of the miR-9-5p mimic while the cells transfected with linc00921-Mut were not decreased (*N* = 3). **g** RIP was performed using an Ago2 antibody in TNBC cells, and the enrichment of linc00921 and miR-9-5p was detected by RT-qPCR (*N* = 3). **P < 0.01, ***P < 0.001

To reveal the functional mechanisms of linc00921 in TNBC, we first detected its subcellular location in HCC-1937 and MDA-MB-231 cells by FISH. The results showed that linc00921 was located in the cytoplasm of HCC-1937 and MDA-MB-231 cells (Fig. 3a). As cytoplasmic lncRNAs can act as competing endogenous RNAs (ceRNAs), we then used bioinformatic algorithms to predict miRNAs that have the potential to be sponged by linc00921. After overlapping the results from starBase (https://starbase.sysu.edu.cn/) and RNAhybrid (https://bibiserv.cebitec.uni-bielefeld.de/rnahybrid/), we found 7 candidate miRNAs possibly binding with linc00921 (Fig. 3b). Then, we detected the expression of these 7 miRNAs in HCC-1937 and MDA-MB-231 cells and analyzed the alterations after transfection with pCDH-linc00921. The results showed that miR-330-5p, miR-326, miR-30a-5p and miR-9-5p were downregulated by overexpression of linc00921 in both HCC-1937 and MDA-MB-231 cells (Fig. 3c). For further determining the precise miRNAs sponged by linc00921, we detected the expression of the above candidate 4 miRNAs in our collected TNBC specimens and then analyzed their correlations with linc00921. The results showed that only miR-9-5p had a significant correlation with linc00921 (Spearman *r* = − 0.484, *P* < 0.001) while the other miRNAs all exhibited no correlation (*P* > 0.05) (Fig. 3d). The above results showed that miR-9-5p might be sponged by linc00921 in TNBC. Thus, we conducted a luciferase reporter assay to further verify whether linc00921 sponged miR-9-5p in TNBC cells. For this goal, luciferase reporters containing wild-type (linc00921-WT) and mutated miR-9-5p binding sites (linc00921-MUT) were constructed and transfected into the HCC-1937 and MDA-MB-231 cells (Fig. 3e). Co-transfection of the miR-9-5p mimic significantly reduced the luciferase activity in the HCC-1937 and MDA-MB-231 cells transfected with linc00921-WT but not in those transfected with linc00921-MUT (Fig. 3f). Then, RIP experiments were performed on the lysates of HCC-1937 and MDA-MB-231 cells by using an antibody against Ago2. As anticipated, the expression levels of linc00921 and miR-9-5p were significantly higher in Ago2 pellets of HCC-1937 and MDA-MB-231 cells than in IgG pellets (Fig. 3g). These findings provide evidence that linc00921 and miR-9-5p occupy the same Ago2 protein to form an RNA-induced silencing complex (RISC) in TNBC cells, indicating that they are members of a gene regulatory axis.

### MiR-9-5p targets the 3′-UTR of LZTS2 mRNA in TNBC cells

**Fig. 4:**
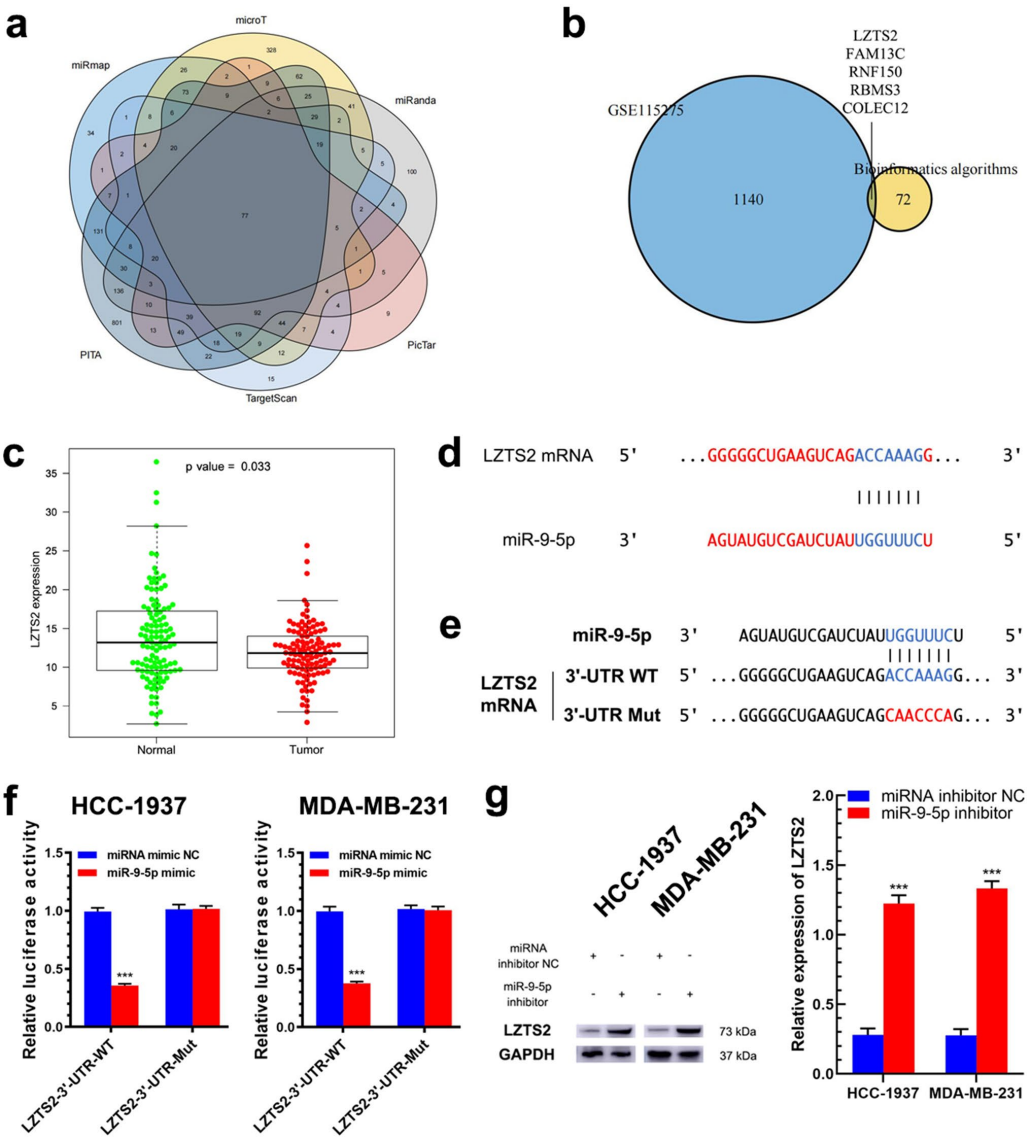
MiR-9-5p targets LZTS2 in TNBC. **a** Overlapping target genes of miR-9-5p, predicted by using 6 bioinformatics algorithms. **b** Predicted target genes of miR-9-5p in TNBC, by overlapping the results from GSE115275 and bioinformatics algorithms. **c** Expression of LZTS2 in TNBC cohort in the TCGA database. **d** Predicted binding between miR-9-5p and LZTS2’s mRNA. **e** Wild-type and mutated sequences of the 3′-UTR of LZTS2’s mRNA for binding miR-9-5p. **f** Schematic illustration showing the effect of miR-9-5p on luciferase reporters containing the wild type or mutated sequence of the 3′-UTR of LZTS2 mRNA (*N* = 3). **g** Effect of miR-9-5p on the expression of LZTS2 in TNBC cells, assayed by western blotting (*N* = 3). ***P < 0.001

Then, the bioinformatic algorithms PITA (https://genie.weizmann.ac.il/pubs/mir07/mir07_data.html), miRmap (https://mirmap.ezlab.org/), microT (http://diana.imis.athena-innovation.gr/DianaTools/index.php?r=microT_CDS/), miRanda (http://www.bioinformatics.com.cn/local_miranda_miRNA_target_prediction_120), PicTar (https://pictar.mdc-berlin.de/) and TargetScan (http://www.targetscan.org/vert_72/) were used to predict the target mRNAs whose 3′-UTR showed the potential to bind with miR-9-5p. After the results were overlapped, 77 genes exhibited the potential to be regulated by miR-9-5p (Fig. 4a and Supplementary Tables 5, 6). Moreover, we analyzed the public GEO profile GSE115275 to further reveal the mRNAs with downregulated expression in TNBC. By setting the fold-change as 2 and P value as 0.01, 2696 validated mRNAs were found to be differentially expressed in TNBC tissues in the GSE115275 profile, among which 1145 had downregulated expression and 1551 had upregulated expression (Supplementary Table 7). By overlapping the results from bioinformatic algorithms and GSE115275, we found 5 candidate genes: LZTS2, FAM13C, RNF150, RBMS3 and COLEC12 (Fig. 4b). Then, leucine zipper tumor suppressor 2 (LZTS2) was chosen for further study, as its precise function and clinical significance in TNBC were unclear. Furthermore, we analyzed the TNBC cohort in The Cancer Genome Atlas (TCGA) database and found that LZTS2 expression in TNBC tissues was lower than that in normal breast tissues (*P* = 0.033; Fig. 4c).

As Fig. 4d shows, there was one hypothetical miR-9-5p binding site in the 3′-UTR of LZTS2 mRNA. To further study the inhibitory function of miR-9-5p on LZTS2, we conducted reporter assays with a luciferase plasmid harboring the 3′-UTR sequence of LZTS2 containing the predicted site for binding miR-9-5p. Furthermore, we generated mutant reporter vectors containing mutations in the miR-9-5p binding site on the LZTS2 3′-UTR (LZTS2-3′-UTR-Mut) (Fig. 4e). These plasmids were transfected into HCC-1937 and MDA-MB-231 cells that were cotransfected with miR-9-5p mimic. As shown in Fig. 4f, miR-9-5p mimic decreased the luciferase activity in the HCC-1937 and MDA-MB-231 cells which were transfected with LZTS2-3′-UTR-WT (0.36 ± 0.02 vs. 0.99 ± 0.03, 0.35 ± 0.02 vs. 1.02 ± 0.04, both *P* < 0.001), but not in those transfected with LZTS2-3′-UTR-Mut (1.02 ± 0.03 vs. 1.01 ± 0.04, *P* = 0.909; 1.02 ± 0.03 vs. 1.01 ± 0.03, *P* = 0.723). This result suggested that miR-9-5p specifically targeted the binding site in the 3′-UTR of LZTS2 mRNA. Moreover, LZTS2 expression in HCC-1937 and MDA-MB-231 cells was increased by co-transfection of a miR-9-5p inhibitor (0.22 ± 0.03 vs. 1.28 ± 0.03, 0.23 ± 0.01 vs. 1.28 ± 0.05, both *P* < 0.001), which was consistent with the result of the luciferase reporter assay (Fig. 4g). These results demonstrated that miR-9-5p could downregulate LZTS2 expression in TNBC by binding to its mRNA 3′-UTR.

### The linc00921/miR-9-5p/LZTS2 axis suppresses the epithelium-to-mesenchymal transition of HCC-1937 and MDA-MB-231 cells

**Fig. 5:**
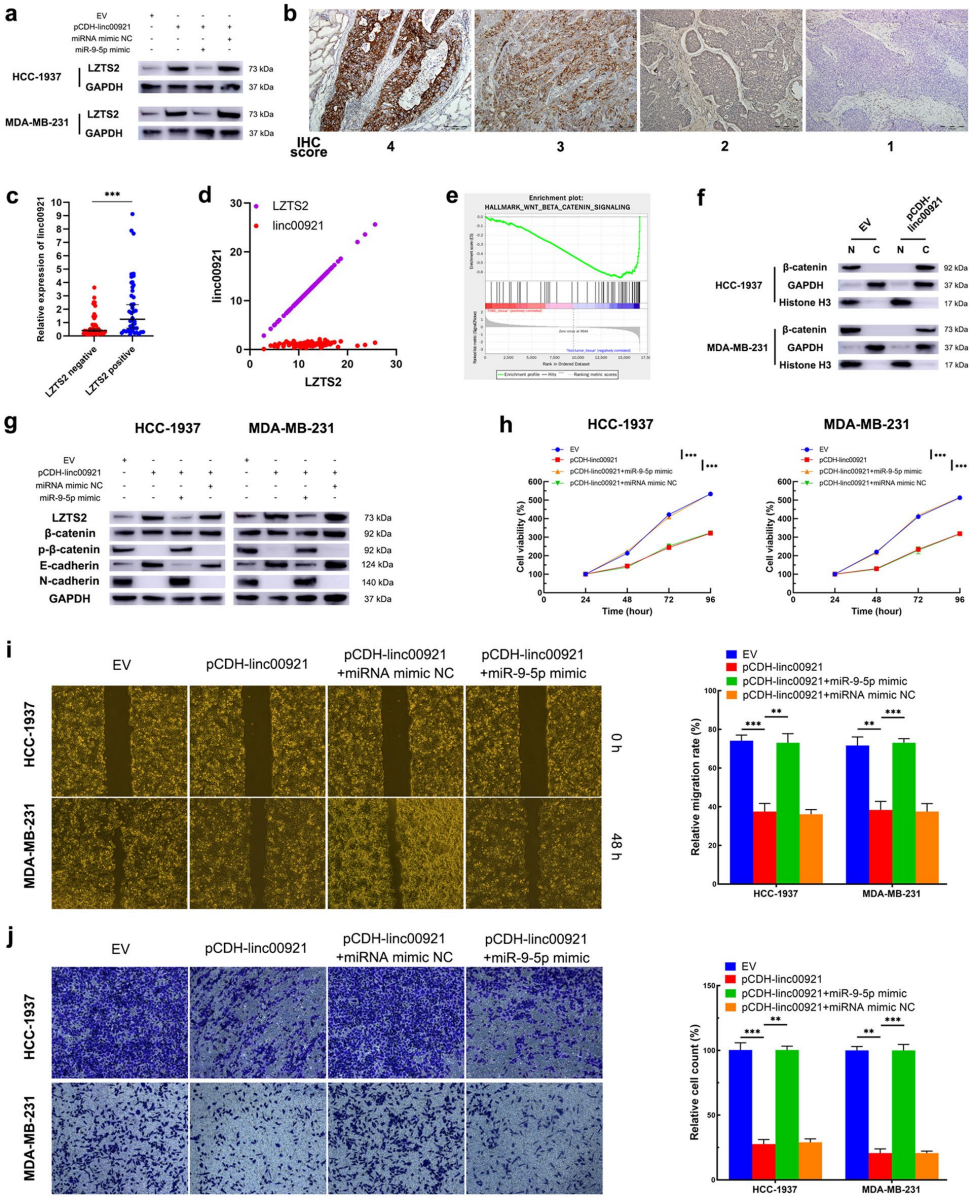
The linc00921/miR-9-5p/LZTS2 axis promotes the proliferation, migration, invasion and EMT in TNBC cells. **a** The effect of the linc00921/miR-9-5p axis on the expression of LZTS2 (*N* = 3). **b** Representative IHC staining of LZTS2 in TNBC tissues. **c** The expression of linc00921 in LZTS2 positive and LZTS2 negative TNBC tissues. **d** Correlation between linc00921 and LZTS2 in TNBC cohort in the TCGA database. **e** LZTS2 was enriched in gene set of Wnt/β-catenin pathway, analyzed by performing GSEA. **f** The effect of linc00921 on the subcellular location of β-catenin in HCC-1937 and MDA-MB-231 cells (*N* = 3). **g** The effect of linc00921 on the expression of EMT-related proteins in HCC-1937 and MDA-MB-231 cells (*N* = 3). **h** The effect of the linc00921/miR-9-5p/LZTS2 axis on the proliferation of HCC-1937 and MDA-MB-231 cells (*N* = 3). **i** The effect of the linc00921/miR-9-5p/LZTS2 axis on the migration of HCC-1937 and MDA-MB-231 cells (*N* = 3). **j** The effect of the linc00921/miR-9-5p/LZTS2 axis on the invasion of HCC-1937 and MDA-MB-231 cells (*N* = 3). **P < 0.01, ***P < 0.001

Based on our above results, we assumed that linc00921 might regulate LZTS2 by sponging miR-9-5p in TNBC and performed miRNA rescue experiments. Western blotting results showed that overexpression of linc00921 upregulated LZTS2 expression in HCC-1937 and MDA-MB-231 cells while the miR-9-5p mimic neutralized this upregulation (Fig. 5a). Furthermore, we assayed the expression of LZTS2 in our collected TNBC specimens by conducting IHC. The IHC staining of LZTS2 mainly was located in the cytoplasm of TNBC cells (Fig. 5b) (H&E staining of the tissue slice is shown in Supplementary Fig. 2A). Among the 95 TNBC patients, 51 (53.68%) exhibited negative expression of LZTS2 while the other 44 (46.62%) exhibited positive expression. Then, the TNBC patients were classified into two groups according to the expression status of LZTS2 to analyze the correlation between LZTS2 and linc00921. The expression of linc00921 was significantly higher in the LZTS2-positive group than in LZTS2-negative group (Fig. 5c). Next, we analyzed the TCGA data and found that linc00921 and LZTS2 were positively correlated in TNBC (Spearman *r* = 0.235, *P* = 0.01; Fig. 5d), which was consistent with the results obtained from our specimens. The above results provide evidence that a linc00921/miR-9-5p/LZTS2 regulatory axis exists in TNBC.

To further identify the underlying downstream pathway of the linc00921/miR-9-5p/LZTS2 axis, we performed gene set enrichment analysis (GSEA) based on GSE115725. The results showed that LZTS2 was enriched in the Wnt/β-catenin pathway gene set which plays crucial roles in epithelial-to-mesenchymal transition (EMT) (Fig. 5e). Since LZTS2 promotes nuclear export of β-catenin [14], we then detected the effect of the linc00921/miR-9-5p/LZTS2 axis on the subcellular location of β-catenin. By conducting nuclear and cytoplasmic fraction isolation experiments followed by western blotting, we found that overexpression of linc00921 significantly decreased the nuclear-cytoplasmic ratio of β-catenin (Fig. 5f). Since nuclear localization was essential for β-catenin to exert its promoting function in EMT, we then assayed whether the linc00921/miR-9-5p/LZTS2 axis affected EMT-related proteins in HCC-1937 and MDA-MB-231 cells. As anticipated, overexpression of linc00921 inhibited phosphorylation of β-catenin, resulting in increased expression of E-cadherin and decreased expression of N-cadherin in HCC-1937 and MDA-MB-231 cells, while co-transfection of miR-9-5p mimic reversed this phenomenon (Fig. 5g). Since phosphorylated β-catenin is essential for cell cycle progression in TNBC [15], we assayed the effect of the linc00921/miR-9-5p/LZTS2 axis on proliferation in HCC-1937 and MDA-MB-231 cells. The proliferation of HCC-1937 and MDA-MB-231 cells was significantly inhibited by overexpression of linc00921, while these effects were abolished by co-transfection of miR-9-5p mimic (Fig. 5h). As EMT has promoting effects on numerous malignant behavior of cancer cells [16], we then detected the effect of the linc00921/miR-9-5p/LZTS2 axis on migration and invasion in HCC-1937 and MDA-MB-231 cells. Furthermore, wound-healing and Transwell Matrigel assay showed that overexpression of linc00921 inhibited migration and invasion of HCC-1937 and MDA-MB-231 cells, while miR-9-5p mimic offset the impact of linc0921 overexpression (Fig. 5i, j). In summary, these results illustrated that the linc00921/miR-9-5p/LZTS2 axis promoted the progression of TNBC cells.

### Overexpression of linc00921 upregulates LZTS2 expression and suppresses tumor growth of TNBC in vivo

**Fig. 6:**
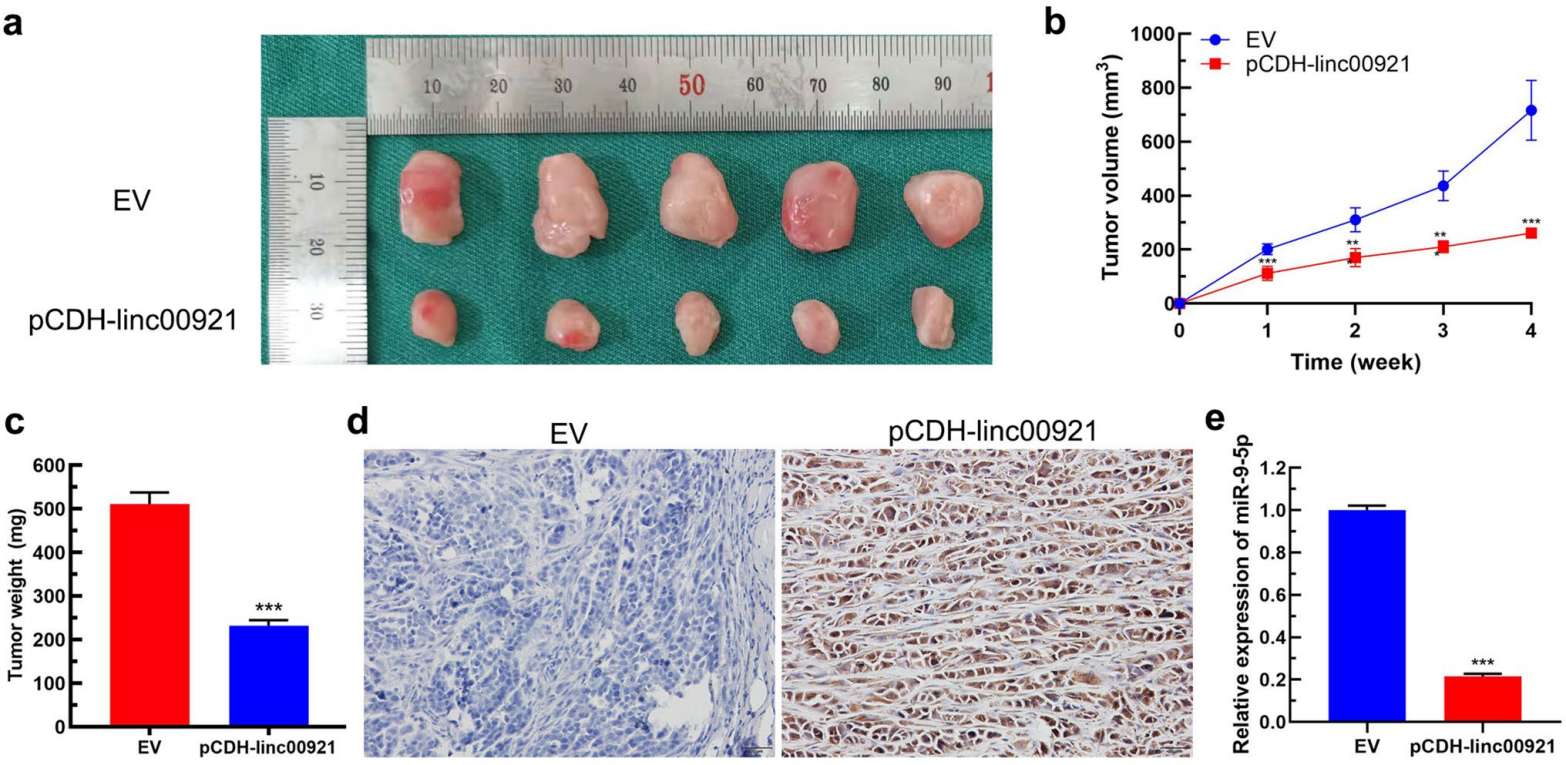
Linc00921 suppresses the progression of TNBC in vivo. **a** Xenograft tumors of sacrificed mice (*N* = 5 for each group). **b** Tumor volumes of xenograft tumors, calculated by using the following formula: 0.5 × L × W^2^** (*N* = 5 for each group). **c** Tumor weights of xenograft tumors at Day 28 (*N* = 5 for each group). **d** Presentative IHC staining of LZTS2 in xenograft tumors of EV transfection group and pCDH-linc00921 transfection group. **e** Relative expression of miR-9-5p in xenograft tumors of EV transfection group and pCDH-linc00921 transfection group (*N* = 5 for each group). ***P < 0.001

Finally, we established a xenograft tumor mouse model to investigate whether linc00921 acted as a tumor-suppressor in vivo. The results showed that the size and weight of tumors in the pCDH-linc00921 group were significantly reduced, compared to those in the EV group (Fig. 6a–c, *P* < 0.01). Moreover, the tumor tissues of the pCDH-linc00921 group showed positive expression of LZTS2 but the EV group showed negative expression of LZTS2 (Fig. 6d) (H&E staining of the tissue slice is shown in Supplementary Fig. 2B). Moreover, the expression of miR-9-5p was significantly lower in the pCDH-linc00921 group than in EV group (Fig. 6e). Taken together, these results suggested that linc00921 could suppress the progression of TNBC in vivo by suppressing the expression of LZTS2.

## Discussion

TNBC is the most aggressive subtype of breast cancer and is not sensitive to endocrine therapy or anti-HER2 agents [17]. However, our lack of knowledge of the dysregulated molecules involved in TNBC limits the improvement of clinical therapeutic efficacy. Accumulating studies have demonstrated that attenuation of tumor-suppressing genes, one of the most frequent cancer hallmarks, contributes to the initiation and progression of TNBC [18]. LncRNAs were recently identified as key regulators of gene expression whose dysregulation has been confirmed to be correlated with carcinogenesis [11]. Herein, revealing the roles of the tumor-suppressing lncRNAs might be conducive to establishing novel treatment strategies for improving the prognosis of TNBC patients. In the present study, we analyzed the public GEO profiles GSE119233 and GSE115275 to verify the key lncRNAs correlated with TNBC. By confirming the expression of the candidate lncRNAs with downregulated expression by RT-qPCR, we finally chose linc00921 for further study since its clinical significance and biological mechanisms in cancers were not clear. Linc00921 was expressed at low levels in approximately 51.58% of our collected TNBC tissues. Furthermore, low expression of LZTS2 was significantly correlated with unsatisfactory clinical parameters of TNBC patients, including higher TNM stage, larger tumor size and positive lymph node metastasis, indicating that it might act as a tumor-suppressing lncRNA in TNBC. Furthermore, by conducting Kaplan–Meier analysis and Cox proportional hazards model, we found that linc00921 could be regarded as an independent biomarker for predicting the prognosis of TNBC patients. To confirm the tumor-suppressing effects of linc00921, we overexpressed linc00921 in TNBC cells by establishing plasmids. Overexpression of linc00921 significantly suppressed the proliferation, migration and invasion ability of TNBC cells. Taken together, these results provide evidence that linc00921 is a tumor-suppressor in TNBC.

Since lncRNAs, especially cytoplasmic lncRNAs, could exert regulatory functions by sponging specific miRNAs [19], we used the bioinformatics algorithms to predict the probable miRNAs potentially sponged by linc00921. Although we found four possible miRNAs which exhibited possibility to be sponged by linc00921, only miR-9-5p was significantly negatively correlated with linc00921 in our collected TNBC tissues, significantly. MiR-9-5p, located at human 1q22, is a recognized miRNA correlated with cancers, but its precise function varies due to the differences in target mRNAs. For instance, miR-9-5p could suppress tumor proliferation and migration in gastric cancer by targeting neuropilin-1 [20], but it acted as an oncogene promoting angiogenesis and invasion by inhibiting SOCS5 in cervical cancer [21]. Regarding TNBC, miR-9-5p was a miRNA with upregulated expression but its functional mechanisms were unclear [22]. Next, we analyzed the overlapping results from bioinformatics algorithms and GSE115275 to predict the target genes of miR-9-5p in TNBC. Among the results, we focused on LZTS2 whose functions in TNBC had not been revealed. LZTS2, also known as LAPSER1, belongs to the LZTS family which is related to transcription and cell cycle regulation [23]. LZTS2 is located at human 10q24.31, which is proximate to the site of the classical tumor suppressor PTEN, indicating that LZTS2 might also exert tumor suppressing effects [24]. The presence of LZTS2 is crucial for preventing spontaneous tumor development, indicating that LZTS2 exhibits tumor-suppressing effects even in tumor initial phase [25]. Emerging studies have demonstrated that the expression of LZTS2 is attenuated or even lost in numerous malignant tumors, such as prostate cancer, non-small cell lung cancer (NSCLC) and colon cancer [26–28]. Since the expression of LZTS2 in TNBC was still unknown, we first detected its expression in our collected specimens. The results showed that LZTS2 exhibited low expression in approximately 53.68% of TNBC specimens. By conducting a luciferase reporter assay, we validate the regulatory effect of miR-9-5p on LZTS2, thus, we hypothesized a linc00921/miR-9-5p/LZTS2 axis in TNBC. By performing miRNA rescue experiments, we observed a linc00921-induced upregulation of LZTS2 expression, which was neutralized by the miR-9-5p mimic, indicating that the linc00921/miR-9-5p/LZTS2 axis indeed existed in TNBC.

EMT refers to the process by which epithelial cancer cells acquire a mesenchymal phenotype [29]. EMT is important for some cancer cells to exhibit malignant behavior, such as invasion, metastasis, immune escape [30]. Activation of the Wnt/β-catenin pathway is crucial for EMT initiation in many kinds of cancers including TNBC [31]. Thyssen et al*.* reported that LZTS2 could interact with β-catenin and thereby promote its nuclear export [14]. Nuclear localization is essential for β-catenin to exhibit its function; thus, nuclear export attenuates the function of β-catenin, which results in deactivation of Wnt/β-catenin pathway [32]. We then detected the effect of the linc00921/miR-9-5p/LZTS2 axis on the expression of EMT-related proteins. The results showed that overexpression of linc00921 upregulated the expression of E-cadherin and downregulated the expression of N-cadherin in TNBC cells. Meanwhile, the above results were reversed by co-transfection of the miR-9-5p mimic. E-cadherin is necessary for epithelial cells to maintain cell–cell adhesion; thus, attenuation of E-cadherin is regarded as a hallmark of EMT [33]. Along with the loss of E-cadherin, upregulated expression of N-cadherin, which is a downstream regulatory target of β-catenin, is another marker of the EMT process in cancers [34]. Taken above, these results provide evidence that linc00921/miR-9-5p/LZTS2 can suppress the β-catenin-mediated EMT process in TNBC.

In conclusion, we confirmed the low expression status and tumor-suppressing function of linc00921 in TNBC. We identified linc00921 as a potential biomarker for predicting the prognosis of TNBC patients. Our findings also corroborated that linc00921 upregulated LZTS2 expression by sponging miR-9-5p and thereby suppressed β-catenin-mediated EMT. This study highlighted the novel clinical significance of the linc00921/miR-9-5p/LZTS2 axis in TNBC.

## Supplementary Information

Below is the link to the electronic supplementary material.

Supplementary Fig. 1 Differentially expressed lncRNAs in TNBC-related GEO profiles. a Differentially expressed lncRNAs in GSE119233. b Differentially expressed lncRNAs in GSE115275. (JPG 600 KB)

Supplementary Fig. 2 The H&E staining of slices of TNBC tissues. a The H&E staining of slices in Fig. 5b. I, II, III, and IV present the slices of IHC score 1, 2, 3, 4 of LZTS2, respectively. b The H&E staining of slices in Fig. 6d. I and II present the slices of EV and Pcdh-linc00921 group, respectively. (JPG 3634 KB)

Supplementary file3 (DOCX 16 KB)

Supplementary file4 (DOCX 15 KB)

Supplementary file5 (DOCX 65 KB)

Supplementary file6 (DOCX 14 KB)

Supplementary file7 (DOCX 90 KB)

Supplementary file8 (DOCX 17 KB)

Supplementary file9 (DOCX 60 KB)

## Data Availability

The analyzed data sets generated during the study are available from the corresponding author on reasonable request.
