# Supplementary figures and images for: Long non-coding RNA linc00921 suppresses tumorigenesis and epithelial-to-mesenchymal transition of triple-negative breast cancer via targeting miR-9-5p/LZTS2 axis
Source: Hum Cell. 2022 Feb 18;35(3):909–23. doi: 10.1007/s13577-022-00685-6 (PMC9013323; doi:10.1007/s13577-022-00685-6)

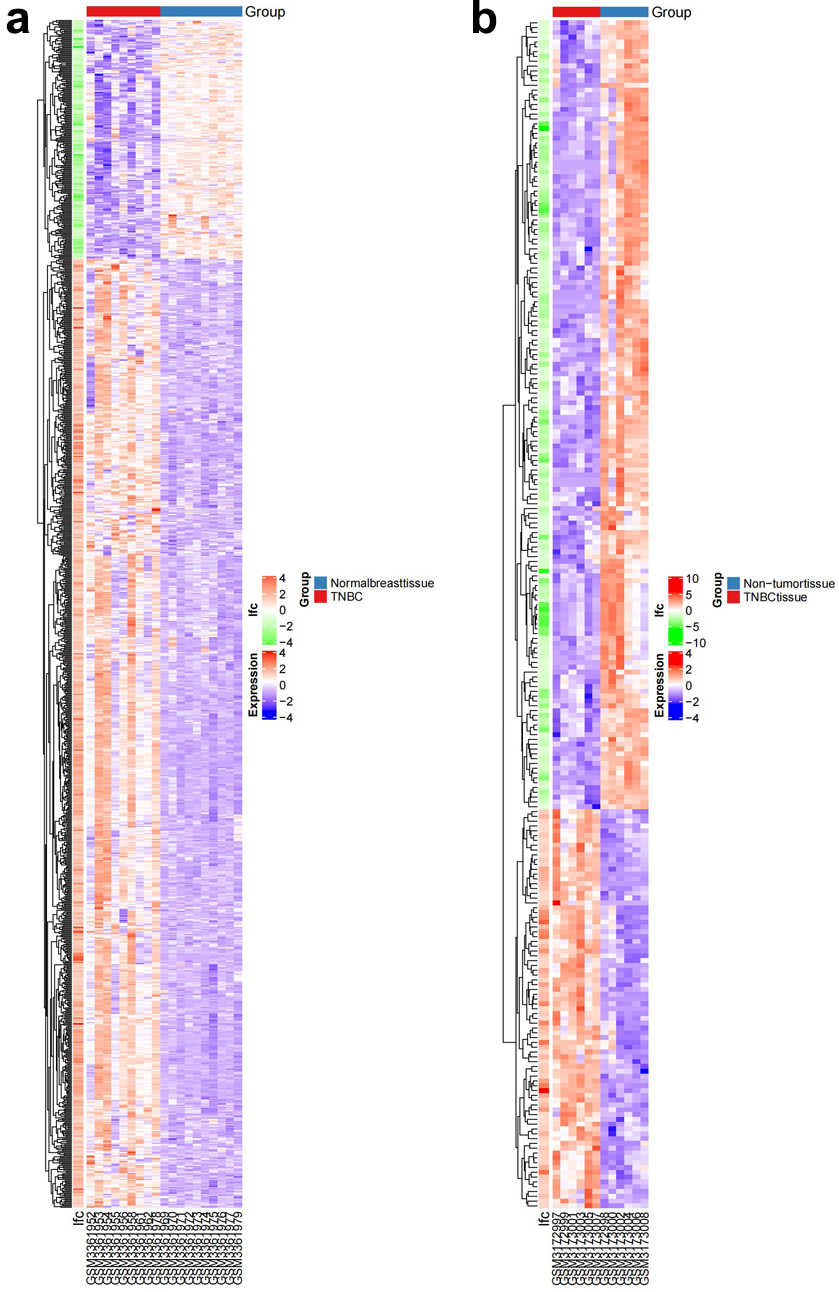

Supplement: Supplementary file 1 — Supplementary Fig. 1 Differentially expressed lncRNAs in TNBC-related GEO profiles. a Differentially expressed lncRNAs in GSE119233. b Differentially expressed lncRNAs in GSE115275. (JPG 600 KB) [file 13577_2022_685_MOESM1_ESM.jpg]

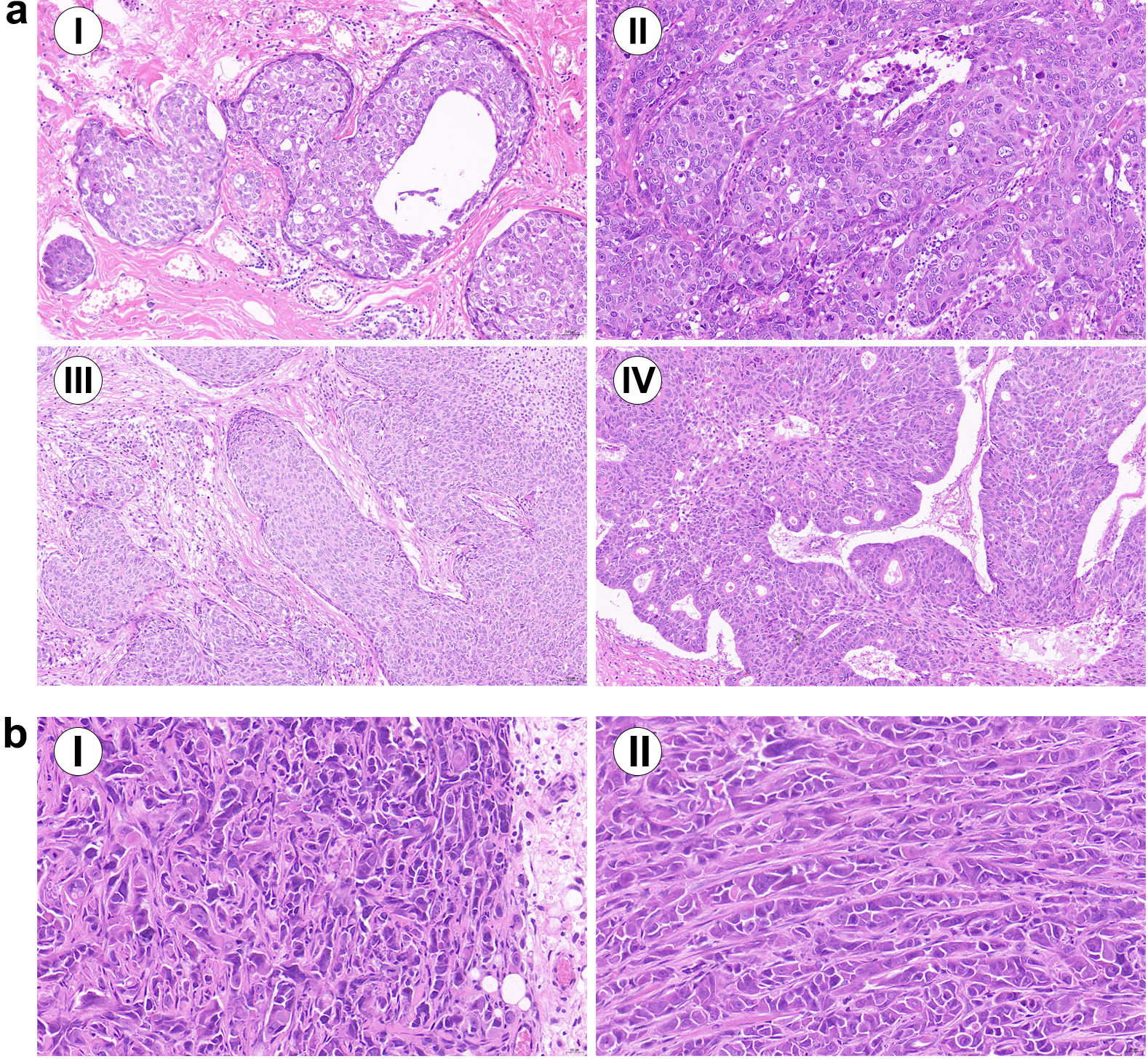

Supplement: Supplementary file 2 — Supplementary Fig. 2 The H&E staining of slices of TNBC tissues. a The H&E staining of slices in Fig. 5b. I, II, III, and IV present the slices of IHC score 1, 2, 3, 4 of LZTS2, respectively. b The H&E staining of slices in Fig. 6d. I and II present the slices of EV and Pcdh-linc00921 group, respectively. (JPG 3634 KB) [file 13577_2022_685_MOESM2_ESM.jpg]
