# Supplementary material for: Long non-coding RNA linc00921 suppresses tumorigenesis and epithelial-to-mesenchymal transition of triple-negative breast cancer via targeting miR-9-5p/LZTS2 axis
Source: Hum Cell. 2022 Feb 18;35(3):909–23. doi: 10.1007/s13577-022-00685-6 (PMC9013323; doi:10.1007/s13577-022-00685-6)
Supplement: Supplementary file 3 — Supplementary file3 (DOCX 16 KB) [file 13577_2022_685_MOESM3_ESM.docx]

**Supplementary Table 1 Primer sequences of lncRNA and mRNAs for RT-qPCR**

| Gene | Refseq | Sequence (5'-3') |
| --- | --- | --- |
| linc00921 | NR_033904.1 | Forward: TGACAGGCCATTTGCCAAGA  Reverse: GAGGATTGTCCTTGGAGCGT |
| PGM5-AS1 | NR_121192.1 | Forward: CCTTGGAATCTACAGGGCCG  Reverse: CGGACAGGCTGAAAGTACCA |
| TRHDE-AS1 | NR_026836.1 | Forward: CCGAGGTGGGAGAGACTAGA  Reverse: CTTAAAGTGCCCAGGGTGGA |
| FGF13-AS1 | NR_038405.1 | Forward: GAGCTGTAAGGAAGCTGAGGG  Reverse: TGCTCCCGATTAGGCTGTCT |
| linc01985 | NR_147054.1 | Forward: TTGCTTGAAATTGTGGCTGGA  Reverse: GGAGGTGCTGAAAGACCACG |
| GAPDH | NG_007073.2 | Forward: CCTGAGGGTTCTTTGTGCTGA  Reverse: AAAGGCTCAACCTTCCCCAT |
