# Supplementary material for: Long non-coding RNA linc00921 suppresses tumorigenesis and epithelial-to-mesenchymal transition of triple-negative breast cancer via targeting miR-9-5p/LZTS2 axis
Source: Hum Cell. 2022 Feb 18;35(3):909–23. doi: 10.1007/s13577-022-00685-6 (PMC9013323; doi:10.1007/s13577-022-00685-6)
Supplement: Supplementary file 4 — Supplementary file4 (DOCX 15 KB) [file 13577_2022_685_MOESM4_ESM.docx]

**Supplementary Table 2 Primer sequences of miRNAs and U6 for RT-qPCR**

| Gene | Forward Sequence (5'-3') |
| --- | --- |
| miR-330-5p | TCTCTGGGCCTGTGTC |
| miR-326 | CTCTGGGCCCTTCCT |
| miR-30a-5p | GCAGTGTAAACATCCTCGAC |
| miR-30d-5p | AGTGTAAACATCCCCGACT |
| miR-30e-5p | CGCAGTGTAAACATCCTTGAC |
| miR-9-5p | GCAGTCTTTGGTTATCTAGCTG |
| miR-34a-5p | GCAGTGGCAGTGTCTTAG |
| U6 | CTCGCTTCGGCAGCACATA |
