# Supplementary material for: Long non-coding RNA linc00921 suppresses tumorigenesis and epithelial-to-mesenchymal transition of triple-negative breast cancer via targeting miR-9-5p/LZTS2 axis
Source: Hum Cell. 2022 Feb 18;35(3):909–23. doi: 10.1007/s13577-022-00685-6 (PMC9013323; doi:10.1007/s13577-022-00685-6)
Supplement: Supplementary file 5 — Supplementary file5 (DOCX 65 KB) [file 13577_2022_685_MOESM5_ESM.docx]

**Supplementary Table 3 Differential lncRNAs in GSE119233 and GSE115275**

| **Downregulated genes in GSE119233** | **Upregulated genes in GSE119233** | **Downregulated genes in GSE115275** | **Upregulated genes in GSE115275** |
| --- | --- | --- | --- |
| LUARIS | LINC01705 | TRHDE-AS1 | LINC01614 |
| TRHDE-AS1 | LINC01929 | LINC02489 | WASIR1 |
| LINC01963 | LINC02657 | LINC02148 | LINC01139 |
| LINC02660 | LINC01195 | LINC01612 | MIR200CHG |
| SNHG29 | LINC01615 | FAM167A-AS1 | LINC00052 |
| PGM5P3-AS1 | LINC02608 | LINC01443 | MKNK1-AS1 |
| THAP9-AS1 | ATP2A1-AS1 | PARAL1 | LINC02544 |
| MIR4453HG | LINC01956 | FAM3D-AS1 | LINC00460 |
| PGM5-AS1 | LINC00305 | LINC01985 | LINC01055 |
| LINC00958 | LINC00337 | LINC02511 | NEBL-AS1 |
| RBPMS-AS1 | LINC02253 | LINC01735 | LINC01711 |
| LINC00842 | GSEC | PGM5-AS1 | LINC01857 |
| FGF13-AS1 | LINC00460 | FGF13-AS1 | GATA3-AS1 |
| TAT-AS1 | LINC01363 | CADM3-AS1 | LNCPRESS1 |
| LINC01985 | LINC01357 | IL1R1-AS1 | PVT1 |
| RHOXF1-AS1 | MAFA-AS1 | BRWD1-AS1 | LINC02446 |
| HOXA-AS2 | LINC02004 | LINC00377 | LINC00160 |
| EBLN3P | LINC01655 | DPP9-AS1 | LNCAROD |
| LINC02607 | ARLNC1 | LINC02066 | LINC00592 |
| LINC01578 | LINC02473 | DIO3OS | LINC02195 |
| LINC01697 | LINC00686 | NKAIN3-IT1 | LINC01675 |
| PCAT18 | LINC01740 | LINC01497 | LINC00930 |
| LINC01128 | TMEM202-AS1 | LINC02634 | LINC01655 |
| LINC02511 | LINC02182 | LINC02082 | C6orf99 |
| PGM5P4-AS1 | SCAT1 | MAGI2-AS3 | LINC02471 |
| POC1B-AS1 | SH3PXD2A-AS1 | SLC14A2-AS1 | LINC01929 |
| RAP2C-AS1 | LINC01833 | EMX2OS | SCAT1 |
| CERNA1 | CASC8 | PGM5P4-AS1 | LINC01388 |
| PCBP1-AS1 | LINC02159 | LINC00484 | DEPDC1-AS1 |
| TPT1-AS1 | LINC02087 | LINC01140 | RNF144A-AS1 |
| LINC02381 | LINC01500 | TPRG1-AS1 | ZFHX2-AS1 |
| TRAM2-AS1 | HAGLROS | TENM3-AS1 | LINC02244 |
| WDFY3-AS2 | MYLK-AS1 | PRR29-AS1 | TRPM2-AS |
| ZNF582-AS1 | SYP-AS1 | RAMP2-AS1 | MYB-AS1 |
| LYPLAL1-AS1 | LINC01784 | LINC00924 | HOXC13-AS |
| LRRC8C-DT | FAM99A | LINC01239 | ELF3-AS1 |
| LINC00662 | OGFRP1 | LINC00261 | LEF1-AS1 |
| LINC00997 | GORAB-AS1 | MIR497HG | LINC02461 |
| FAM85B | DSCR9 | LINC00840 | GAPLINC |
| SUCLG2-AS1 | SNHG25 | LINC02104 | C2orf27B |
| NR2F1-AS1 | SLC5A4-AS1 | VLDLR-AS1 | LINC01268 |
| NCKAP5-AS2 | LINC02434 | NGF-AS1 | LINC01822 |
| LINC01137 | LINC00689 | PCAT19 | LINC01643 |
| TSBP1-AS1 | LINC01222 | OXCT1-AS1 | MIR5689HG |
| EIF3J-DT | IL1R1-AS1 | LINC01273 | LINC00113 |
| LINC00517 | LINC02396 | LINC01715 | DGUOK-AS1 |
| MAGI2-AS3 | KIAA1614-AS1 | LINC01301 | HMGA1P4 |
| MAP4K3-DT | LINC00942 | PTENP1-AS | ATP2A1-AS1 |
| ATP2B1-AS1 | LINC01376 | LINC02515 | GRPEL2-AS1 |
| UBL7-AS1 | FAM224B | LINC01305 | TMEM92-AS1 |
| ILF3-DT | TDRG1 | MIR193BHG | TDRKH-AS1 |
| LINC02522 | RMRP | LINC01844 | C3orf35 |
| HOXC-AS1 | LINC02574 | HAND2-AS1 | FAM225B |
| LINC01778 | ENOX1-AS2 | DISC1FP1 | MMP25-AS1 |
| LINC02190 | GAPLINC | WWC2-AS2 | LINC02201 |
| LINC02428 | FAM83C-AS1 | LINC01197 | FAM225A |
| LNCTAM34A | LINC00698 | LINC01550 | LINC01771 |
| LINC01874 | SNHG3 | ISPD-AS1 | SERPINB9P1 |
| FAM66E | LINC01937 | LINC02382 | OGFRP1 |
| ZNF710-AS1 | LINC00880 | LINC01412 | LINC01431 |
| HOTAIRM1 | GFOD1-AS1 | LINC01616 | LINC01048 |
| TTC39A-AS1 | LINC01770 | ZNF436-AS1 | SREBF2-AS1 |
| LINC01011 | LINC02645 | HOXA-AS2 | HOXB-AS3 |
| SOD2-OT1 | FOXD3-AS1 | C5orf64 | LINC01705 |
| THRB-AS1 | LINC02588 | LINC01354 | LINC02280 |
| CKMT2-AS1 | H1FX-AS1 | MESTIT1 | ZRANB2-AS1 |
| GATA3-AS1 | LINC01455 | BVES-AS1 | TMEM9B-AS1 |
| CACNA1G-AS1 | USP2-AS1 | CCDC26 | GTSE1-DT |
| RMST | LINC01311 | LINC02580 | SGO1-AS1 |
| ST7-AS1 | LINC01191 | WDFY3-AS2 | HOXA11-AS |
| HHIP-AS1 | HOXA-AS3 | LINC01402 | LINC01934 |
| LINC01527 | CYP1B1-AS1 | LINC01091 | LINC00659 |
| STARD4-AS1 | LNCOC1 | MIR99AHG | LINC01168 |
| TESC-AS1 | LINC00928 | LRRC8C-DT | LINC01389 |
| LINC00920 | TOB1-AS1 | LINC00534 | LINC02317 |
| LINC00504 | LEF1-AS1 | LINC00028 | PABPC4-AS1 |
| LINC01091 | LINC01208 | LINC02227 | LINC01094 |
| CASC2 | LINC01136 | MAST4-AS1 | FOXD3-AS1 |
| ELN-AS1 | LINC00362 | HHIP-AS1 | OIP5-AS1 |
| LINC01252 | SZT2-AS1 | RBPMS-AS1 | OVAAL |
| LINC01070 | HCG21 | LINC00639 | SAMD12-AS1 |
| OLMALINC | LINC00654 | PICSAR | LINC00954 |
| DENND6A-AS1 | LINC01063 | ZBED3-AS1 | NRAV |
| GRIK1-AS1 | LINC01273 | TEX41 | LINC00539 |
| INTS6-AS1 | LINC02490 | SENCR | LINC00205 |
| LINC01515 | PRKCZ-AS1 | LINC01043 | WASIR2 |
| LINC01135 | MIR155HG | KIF25-AS1 | LINC00963 |
| WARS2-IT1 | LINC00518 | B4GALT1-AS1 | CT62 |
| MKLN1-AS | KRT73-AS1 | DDX11-AS1 | PIK3CD-AS1 |
| LINC01060 | LINC02336 | HLX-AS1 | LINC02668 |
| LINC01116 | LINC-ROR | EIF1B-AS1 | LINC00939 |
| SH3BP5-AS1 | LINC02038 | LINC00654 | LINC00426 |
| RASSF8-AS1 | ZKSCAN2-DT | LINC00911 | NAPA-AS1 |
| LINC01235 | LINC01714 | NR2F1-AS1 | RCAN3AS |
| RBMS3-AS3 | PRR7-AS1 | LINC01952 | LINC01054 |
| MAST4-AS1 | LINC01015 | ZEB2-AS1 | HOXC-AS3 |
| LINC01028 | ISPD-AS1 | SFTPD-AS1 | UCKL1-AS1 |
| PANCR | LINC02570 | RBM26-AS1 | GLIDR |
| ZRANB2-AS1 | LINC02269 | LMO7DN-IT1 | PANDAR |
| DHRS4-AS1 | MIR133A1HG | LYPLAL1-AS1 |  |
| LINC00616 | SPACA6P-AS | RRM1-AS1 |  |
| LINC01412 | LINC02347 | LINC02251 |  |
| NKAIN3-IT1 | MAMDC2-AS1 | ZFPM2-AS1 |  |
| UBA6-AS1 | GATA6-AS1 | ARHGAP5-AS1 |  |
| MATN1-AS1 | LINC00479 | COL4A2-AS1 |  |
| EIF1B-AS1 | PLCL2-AS1 | LINC00893 |  |
| SREBF2-AS1 | LINC01182 | LINC01856 |  |
| NAV2-AS1 | USP12-AS2 | CHL1-AS2 |  |
| SACS-AS1 | LINC00334 | SMAD5-AS1 |  |
| LINC00266-4P | LINC02280 | GNG12-AS1 |  |
| INKA2-AS1 | LINC02167 | LINC01504 |  |
| LINC01298 | APOBEC3B-AS1 | INKA2-AS1 |  |
| KMT2E-AS1 | LINC00523 | CYP1B1-AS1 |  |
| LINC01876 | LINC00511 | LINC02336 |  |
| RFX3-AS1 | LINC02092 | KCNJ2-AS1 |  |
| LINC02483 | RUNDC3A-AS1 | HOTAIRM1 |  |
| GMDS-DT | FEZF1-AS1 | LINC01541 |  |
| FGD5-AS1 | IGFL2-AS1 | ZBTB20-AS4 |  |
| TMEM44-AS1 | LINC01342 | LINC01558 |  |
| KCNJ2-AS1 | LINC00442 | TRIM52-AS1 |  |
| LINC02606 | LINC00297 | EPB41L4A-DT |  |
| LMO7-AS1 | TSPEAR-AS1 | ZNF571-AS1 |  |
| LINC00377 | FAM27E3 | LINC02005 |  |
| DLGAP1-AS5 | SAMSN1-AS1 | LINC01485 |  |
| DICER1-AS1 | LINC00462 | MSC-AS1 |  |
| BAALC-AS1 | RAPGEF4-AS1 | RNF217-AS1 |  |
| LINC02014 | LINC01444 | CAHM |  |
| LINC02246 | LINC00629 | LINC02016 |  |
| FAM198B-AS1 | EIF1AX-AS1 | TYMSOS |  |
| LINC01990 | LINC00887 | PRICKLE2-AS3 |  |
| LINC01165 | TTTY3B | LINC00656 |  |
| FAM157C | LINC02197 | CERS3-AS1 |  |
| LINC02586 | MIR503HG | LINC01625 |  |
| CHKB-DT | LINC01746 | PSG8-AS1 |  |
| LINC01481 | LINC01255 | LINGO1-AS1 |  |
| ZFHX4-AS1 | LINC02332 | HOXD-AS2 |  |
| LINC00630 | LINC01283 | FAM53B-AS1 |  |
| LINC00266-1 | MIR137HG | LINC01474 |  |
| LINC00667 | FARP1-AS1 | LINC00882 |  |
| FAM160A1-DT | LINC01153 | RAB11B-AS1 |  |
| LINC01863 | LNX1-AS1 | ITGB1-DT |  |
| LINC02412 | LINC01068 | LINC01093 |  |
| SNHG8 | LINC02257 | STK24-AS1 |  |
| STARD7-AS1 | LINC00443 | COL18A1-AS2 |  |
| FTX | ZMIZ1-AS1 | TMEM161B-AS1 |  |
| RERE-AS1 | LIX1-AS1 | HELLPAR |  |
| FRMD6-AS1 | LINC01613 | PCDH9-AS1 |  |
| TRPM2-AS | LINC00028 | SGMS1-AS1 |  |
| GATA2-AS1 | LINC01732 | LINC01688 |  |
| PAPPA-AS2 | OVOL1-AS1 | LINC00667 |  |
| OR2A1-AS1 | PTCSC3 | RERE-AS1 |  |
| LINC02062 | NHS-AS1 | NR2F2-AS1 |  |
| LINC01095 | LINC01978 | LINC01957 |  |
| H19 | LINC01588 | IDH1-AS1 |  |
| LINC01184 | NAV2-AS4 | RB1-DT |  |
| LINC00863 | LINC02331 | PRRT3-AS1 |  |
| LINC02019 | LINC02543 | IL6R-AS1 |  |
| SEC24B-AS1 | LINC01471 | C4A-AS1 |  |
| SPINT1-AS1 | LINC01006 | LINC01228 |  |
| BDNF-AS | LINC00626 | FOXN3-AS1 |  |
| LINC00921 | LINC02590 | TUSC8 |  |
| KCNMA1-AS3 | LINC00524 | CLCA4-AS1 |  |
| CCND2-AS1 | LINC02422 | LINC02420 |  |
| LINC00884 | MAFG-DT | LINC02237 |  |
| OSMR-AS1 | LINC00648 | TRAF3IP2-AS1 |  |
| NRAV | LINC02028 | GHRLOS |  |
| RPARP-AS1 | LINC01828 | LINC01081 |  |
| RTCA-AS1 | LINC02400 | MEG8 |  |
| WWC3-AS1 | FAM181A-AS1 | LINC02514 |  |
| PWRN1 | ST3GAL5-AS1 | ZNF503-AS1 |  |
| LINC01237 | MIR124-2HG | LINC01800 |  |
| MIR22HG | HMMR-AS1 | ATP1B3-AS1 |  |
| GAS1RR | LINC01355 | NUTM2A-AS1 |  |
| LINC00571 | LINC01657 | FAM223A |  |
| LINC01356 | PITPNM2-AS1 | LINC01275 |  |
| LAMA5-AS1 | LINC00210 | HMGN3-AS1 |  |
| LINC01894 | LINC02557 | LINC01016 |  |
| LINC00581 | MDC1-AS1 | BSN-DT |  |
| WAC-AS1 | LINC01913 | MIR4453HG |  |
|  | LINC02074 | BDNF-AS |  |
|  | LINC02102 | LINC02018 |  |
|  | MRGPRF-AS1 |  |  |
|  | LINC01695 |  |  |
|  | COX10-AS1 |  |  |
|  | LINC02554 |  |  |
|  | EPHA1-AS1 |  |  |
|  | ELF3-AS1 |  |  |
|  | MIR100HG |  |  |
|  | ATP2C2-AS1 |  |  |
|  | GASAL1 |  |  |
|  | LINC01388 |  |  |
|  | LINC00342 |  |  |
|  | HAGLR |  |  |
|  | LINC01081 |  |  |
|  | LINC01210 |  |  |
|  | LINC01782 |  |  |
|  | PTCSC2 |  |  |
|  | LINC01687 |  |  |
|  | LINC02287 |  |  |
|  | LINC00205 |  |  |
|  | LINC02556 |  |  |
|  | DBH-AS1 |  |  |
|  | LINC00607 |  |  |
|  | LINC02153 |  |  |
|  | KDM4A-AS1 |  |  |
|  | FAM138A |  |  |
|  | RCC2-AS1 |  |  |
|  | ITGB1-DT |  |  |
|  | LINC02544 |  |  |
|  | LINC02578 |  |  |
|  | MIR3150BHG |  |  |
|  | FOXP4-AS1 |  |  |
|  | LINC01475 |  |  |
|  | LINC00115 |  |  |
|  | LINC01716 |  |  |
|  | TTTY6B |  |  |
|  | LINC00895 |  |  |
|  | DSCAM-AS1 |  |  |
|  | LINC01389 |  |  |
|  | USP12-AS1 |  |  |
|  | LINC02443 |  |  |
|  | LGALS8-AS1 |  |  |
|  | LINC01919 |  |  |
|  | LINC02192 |  |  |
|  | LINC01797 |  |  |
|  | PCAT6 |  |  |
|  | NAV2-AS3 |  |  |
|  | CERS6-AS1 |  |  |
|  | BBOX1-AS1 |  |  |
|  | LINC01300 |  |  |
|  | LINC02010 |  |  |
|  | LINC01633 |  |  |
|  | LINC01232 |  |  |
|  | LINC00269 |  |  |
|  | LINC01204 |  |  |
|  | LINC01747 |  |  |
|  | MIR1302-2HG |  |  |
|  | LINP1 |  |  |
|  | LINC01415 |  |  |
|  | LINC00539 |  |  |
|  | ITGA6-AS1 |  |  |
|  | CLEC12A-AS1 |  |  |
|  | FRGCA |  |  |
|  | TMEM212-AS1 |  |  |
|  | ZSWIM8-AS1 |  |  |
|  | LINC01141 |  |  |
|  | LINC02323 |  |  |
|  | LINC02526 |  |  |
|  | LINC01505 |  |  |
|  | AQP4-AS1 |  |  |
|  | MID1IP1-AS1 |  |  |
|  | NAV2-AS5 |  |  |
|  | LINC01757 |  |  |
|  | LINC01230 |  |  |
|  | LINC00106 |  |  |
|  | KCNMA1-AS2 |  |  |
|  | TMEM92-AS1 |  |  |
|  | LINC02601 |  |  |
|  | LINC00567 |  |  |
|  | LINC01033 |  |  |
|  | SMIM10L2B-AS1 |  |  |
|  | MELTF-AS1 |  |  |
|  | GPC6-AS2 |  |  |
|  | SALRNA1 |  |  |
|  | LINC00317 |  |  |
|  | LINC02003 |  |  |
|  | EDRF1-DT |  |  |
|  | FAM230J |  |  |
|  | LINC00877 |  |  |
|  | CLMAT3 |  |  |
|  | SHANK2-AS2 |  |  |
|  | MYO16-AS1 |  |  |
|  | LINC01847 |  |  |
|  | LINC01853 |  |  |
|  | EXOSC10-AS1 |  |  |
|  | LINC02362 |  |  |
|  | LINC01952 |  |  |
|  | PDYN-AS1 |  |  |
|  | LINC00839 |  |  |
|  | UNC5B-AS1 |  |  |
|  | LINC01193 |  |  |
|  | LINC02559 |  |  |
|  | FGF14-AS1 |  |  |
|  | LINC01974 |  |  |
|  | LINC02639 |  |  |
|  | LINC00645 |  |  |
|  | PSMA3-AS1 |  |  |
|  | LINC01133 |  |  |
|  | LINC02341 |  |  |
|  | LINC02318 |  |  |
|  | LINC02655 |  |  |
|  | KIF9-AS1 |  |  |
|  | LINC02550 |  |  |
|  | LINC01413 |  |  |
|  | LINC02391 |  |  |
|  | SSTR5-AS1 |  |  |
|  | LINC01591 |  |  |
|  | LINC01656 |  |  |
|  | LHFPL3-AS1 |  |  |
|  | PCAT1 |  |  |
|  | ITPKB-AS1 |  |  |
|  | LINC01366 |  |  |
|  | LINC00454 |  |  |
|  | LINC02538 |  |  |
|  | ARHGEF7-AS2 |  |  |
|  | ELFN1-AS1 |  |  |
|  | LINC00491 |  |  |
|  | LYPLAL1-DT |  |  |
|  | ZMYND10-AS1 |  |  |
|  | LINC01192 |  |  |
|  | LINC02553 |  |  |
|  | LINC00638 |  |  |
|  | LINC00421 |  |  |
|  | C5orf66-AS1 |  |  |
|  | DDX11-AS1 |  |  |
|  | PITRM1-AS1 |  |  |
|  | SSSCA1-AS1 |  |  |
|  | BOLA3-AS1 |  |  |
|  | CCDC26 |  |  |
|  | HOXC-AS2 |  |  |
|  | LINC02576 |  |  |
|  | TLR8-AS1 |  |  |
|  | LINC01048 |  |  |
|  | G2E3-AS1 |  |  |
|  | LINC00408 |  |  |
|  | MAP3K14-AS1 |  |  |
|  | LINC01497 |  |  |
|  | LINC00943 |  |  |
|  | RPL34-AS1 |  |  |
|  | TONSL-AS1 |  |  |
|  | TTTY8B |  |  |
|  | TUSC7 |  |  |
|  | ZBTB20-AS5 |  |  |
|  | MIR7-3HG |  |  |
|  | LINC02055 |  |  |
|  | CD200R1L-AS1 |  |  |
|  | LINC00029 |  |  |
|  | LINC01132 |  |  |
|  | FAM138D |  |  |
|  | LINC01271 |  |  |
|  | LINC01771 |  |  |
|  | LINC02401 |  |  |
|  | LINC02076 |  |  |
|  | MIR200CHG |  |  |
|  | LINC02354 |  |  |
|  | LINC02392 |  |  |
|  | IL10RB-DT |  |  |
|  | LINC00461 |  |  |
|  | LINC00898 |  |  |
|  | LINC01563 |  |  |
|  | LINC01678 |  |  |
|  | SRGAP2-AS1 |  |  |
|  | LINC02289 |  |  |
|  | LINC02271 |  |  |
|  | EGFLAM-AS1 |  |  |
|  | PRKCA-AS1 |  |  |
|  | KIRREL3-AS2 |  |  |
|  | CSE1L-AS1 |  |  |
|  | C6orf47-AS1 |  |  |
|  | LINC01866 |  |  |
|  | LINC02217 |  |  |
|  | CYTOR |  |  |
|  | BCAR4 |  |  |
|  | LINC01082 |  |  |
|  | LINC00349 |  |  |
|  | TRPC7-AS1 |  |  |
|  | BMF-AS1 |  |  |
|  | LINC02532 |  |  |
|  | LINC01814 |  |  |
|  | LINC01150 |  |  |
|  | BAIAP2-DT |  |  |
|  | LINC00467 |  |  |
|  | HNF1A-AS1 |  |  |
|  | ARHGEF3-AS1 |  |  |
|  | LINC00664 |  |  |
|  | LINC-PINT |  |  |
|  | LINC02177 |  |  |
|  | LINC02438 |  |  |
|  | LINC01102 |  |  |
|  | LINC02126 |  |  |
|  | LINC00602 |  |  |
|  | MPPED2-AS1 |  |  |
|  | LINC02623 |  |  |
|  | LINC01163 |  |  |
|  | LINC02415 |  |  |
|  | MKX-AS1 |  |  |
|  | NIPBL-DT |  |  |
|  | LINC00670 |  |  |
|  | LINC02068 |  |  |
|  | ARHGAP26-AS1 |  |  |
|  | THOC7-AS1 |  |  |
|  | LINC00951 |  |  |
|  | SND1-IT1 |  |  |
|  | LINC00937 |  |  |
|  | LINC00671 |  |  |
|  | A1BG-AS1 |  |  |
|  | LINC02429 |  |  |
|  | LINC01121 |  |  |
|  | LINC01544 |  |  |
|  | LINC01582 |  |  |
|  | MIR4713HG |  |  |
|  | UVRAG-DT |  |  |
|  | LINC02470 |  |  |
|  | LINC01029 |  |  |
|  | LINC02317 |  |  |
|  | TAPT1-AS1 |  |  |
|  | LINC02591 |  |  |
|  | NTM-AS1 |  |  |
|  | WWTR1-AS1 |  |  |
|  | LINC01433 |  |  |
|  | PVT1 |  |  |
|  | SNHG9 |  |  |
|  | APOA1-AS |  |  |
|  | SATB2-AS1 |  |  |
|  | GLYCTK-AS1 |  |  |
|  | LINC01766 |  |  |
|  | PCDH9-AS4 |  |  |
|  | LINC00703 |  |  |
|  | MCM3AP-AS1 |  |  |
|  | TPM1-AS |  |  |
|  | NRIR |  |  |
|  | CCDC37-DT |  |  |
|  | LINC00589 |  |  |
|  | TSPOAP1-AS1 |  |  |
|  | DLG1-AS1 |  |  |
|  | MEG3 |  |  |
|  | SPRY4-AS1 |  |  |
|  | DCST1-AS1 |  |  |
|  | KIAA2012-AS1 |  |  |
|  | HIPK1-AS1 |  |  |
|  | SLC12A5-AS1 |  |  |
|  | LINC01834 |  |  |
|  | FGF12-AS2 |  |  |
|  | CPNE8-AS1 |  |  |
|  | ZFY-AS1 |  |  |
|  | NDUFB2-AS1 |  |  |
|  | FOXD2-AS1 |  |  |
|  | TTTY14 |  |  |
|  | PRKRA-AS1 |  |  |
|  | LINC01303 |  |  |
|  | KIRREL3-AS3 |  |  |
|  | LINC01031 |  |  |
|  | FOXCUT |  |  |
|  | LINC01215 |  |  |
|  | NARF-AS1 |  |  |
|  | LINC00508 |  |  |
|  | SNHG10 |  |  |
|  | OSBPL10-AS1 |  |  |
|  | LDLRAD4-AS1 |  |  |
|  | NPSR1-AS1 |  |  |
|  | LINC01040 |  |  |
|  | LINC01039 |  |  |
|  | ARRDC3-AS1 |  |  |
|  | LINC01099 |  |  |
|  | LINC02519 |  |  |
|  | LINC02240 |  |  |
|  | LINC01635 |  |  |
|  | LINC01762 |  |  |
|  | LINC00596 |  |  |
|  | LMCD1-AS1 |  |  |
|  | LINC00466 |  |  |
|  | LINC02243 |  |  |
|  | C2CD4D-AS1 |  |  |
|  | MYO3B-AS1 |  |  |
|  | LINC01922 |  |  |
|  | NDUFA6-DT |  |  |
|  | GHRLOS |  |  |
|  | MCPH1-AS1 |  |  |
|  | LINC01888 |  |  |
|  | CARD8-AS1 |  |  |
|  | SMIM25 |  |  |
|  | LINC02411 |  |  |
|  | FAM95B1 |  |  |
|  | SBF2-AS1 |  |  |
|  | LINC02088 |  |  |
|  | KCNMB2-AS1 |  |  |
|  | LINC01206 |  |  |
|  | LINC01896 |  |  |
|  | HCG14 |  |  |
|  | BMP7-AS1 |  |  |
|  | LINC01315 |  |  |
|  | LINC00345 |  |  |
|  | LINC02020 |  |  |
|  | HAS2-AS1 |  |  |
|  | LINC01727 |  |  |
|  | RBMS3-AS2 |  |  |
|  | LINC01097 |  |  |
|  | LINC01672 |  |  |
|  | INO80-AS1 |  |  |
|  | LINC00452 |  |  |
|  | ARHGEF7-AS1 |  |  |
|  | SOCS2-AS1 |  |  |
|  | LINC00934 |  |  |
|  | LINC02367 |  |  |
|  | TENM3-AS1 |  |  |
|  | CT62 |  |  |
|  | LINC00608 |  |  |
|  | LINC01712 |  |  |
|  | ZCCHC23 |  |  |
|  | LINC01824 |  |  |
|  | SGO1-AS1 |  |  |
|  | LINC02022 |  |  |
|  | HMGA1P4 |  |  |
|  | LINC00635 |  |  |
|  | GNAS-AS1 |  |  |
|  | LINC02363 |  |  |
|  | SNHG6 |  |  |
|  | CASC22 |  |  |
|  | LINC01393 |  |  |
|  | ZEB2-AS1 |  |  |
|  | LIX1L-AS1 |  |  |
|  | LINC02205 |  |  |
|  | HPYR1 |  |  |
|  | LINC02484 |  |  |
|  | LINC01586 |  |  |
|  | TTLL10-AS1 |  |  |
|  | LPGAT1-AS1 |  |  |
|  | C9orf139 |  |  |
|  | LINC01939 |  |  |
|  | LINC01918 |  |  |
|  | LINC00555 |  |  |
|  | LINC02482 |  |  |
|  | TMEM220-AS1 |  |  |
|  | LINC00102 |  |  |
|  | LINC00929 |  |  |
|  | LINC01864 |  |  |
|  | GARS-DT |  |  |
|  | TMEM51-AS1 |  |  |
|  | FLNB-AS1 |  |  |
|  | LINC02426 |  |  |
|  | TTTY7 |  |  |
|  | LINC00668 |  |  |
|  | LINC02505 |  |  |
|  | LINC02613 |  |  |
|  | LINC01933 |  |  |
|  | ITPR1-DT |  |  |
|  | HAND2-AS1 |  |  |
|  | C15orf53 |  |  |
|  | LINC02629 |  |  |
|  | PURPL |  |  |
|  | LINC01793 |  |  |
|  | ZNF295-AS1 |  |  |
|  | ODC1-DT |  |  |
|  | CHRM3-AS2 |  |  |
|  | SOX9-AS1 |  |  |
|  | STPG3-AS1 |  |  |
|  | LINC02475 |  |  |
|  | PABPC1L2B-AS1 |  |  |
|  | LINC01304 |  |  |
|  | LINC02023 |  |  |
|  | LINC00293 |  |  |
|  | MIR4435-2HG |  |  |
|  | LINC01794 |  |  |
|  | SLC8A1-AS1 |  |  |
|  | LINC02322 |  |  |
|  | LINC01502 |  |  |
|  | CACNA1C-AS1 |  |  |
|  | PKN2-AS1 |  |  |
|  | LINC01146 |  |  |
|  | MYLK-AS2 |  |  |
|  | SNHG21 |  |  |
|  | LINC00927 |  |  |
|  | NAMA |  |  |
|  | MHENCR |  |  |
|  | FAM242C |  |  |
|  | OOEP-AS1 |  |  |
|  | LINC00158 |  |  |
|  | FMR1-AS1 |  |  |
|  | LINC02149 |  |  |
|  | STEAP2-AS1 |  |  |
|  | MIAT |  |  |
|  | LINC00853 |  |  |
|  | LINC00410 |  |  |
|  | SRD5A3-AS1 |  |  |
|  | CNTFR-AS1 |  |  |
|  | LINC00052 |  |  |
|  | LINC01023 |  |  |
|  | LINC01144 |  |  |
|  | LINC01297 |  |  |
|  | LINC02436 |  |  |
|  | LINC01970 |  |  |
|  | LINC00907 |  |  |
|  | CNTN4-AS1 |  |  |
|  | GAS6-AS1 |  |  |
|  | TARID |  |  |
|  | FLG-AS1 |  |  |
|  | BSN-DT |  |  |
|  | ATP1B3-AS1 |  |  |
|  | RNF139-AS1 |  |  |
|  | LINC01855 |  |  |
|  | ERICH3-AS1 |  |  |
|  | HUNK-AS1 |  |  |
|  | LINC02032 |  |  |
|  | PEX5L-AS1 |  |  |
|  | RRM1-AS1 |  |  |
|  | LINC00534 |  |  |
|  | VPS33B-DT |  |  |
|  | MUC20-OT1 |  |  |
|  | LINC00570 |  |  |
|  | PPP1R12A-AS1 |  |  |
|  | LY6E-DT |  |  |
|  | LINC00313 |  |  |
|  | CAMTA1-DT |  |  |
|  | MCHR2-AS1 |  |  |
|  | MIR646HG |  |  |
|  | LINC02665 |  |  |
|  | LINC01524 |  |  |
|  | MIR3681HG |  |  |
|  | ZNF571-AS1 |  |  |
|  | DDX39B-AS1 |  |  |
|  | LINC00572 |  |  |
|  | MIR210HG |  |  |
|  | LINC02418 |  |  |
|  | LINC00982 |  |  |
|  | TTTY19 |  |  |
|  | MIR762HG |  |  |
|  | LINC01608 |  |  |
|  | LINC01301 |  |  |
|  | LINC01715 |  |  |
|  | LINC00390 |  |  |
|  | FAM230A |  |  |
|  | LINC00910 |  |  |
|  | LINC01276 |  |  |
|  | NKX2-2-AS1 |  |  |
|  | SDCBP2-AS1 |  |  |
|  | DIO3OS |  |  |
|  | DLGAP2-AS1 |  |  |
|  | LINC01606 |  |  |
|  | LINC01391 |  |  |
|  | NALT1 |  |  |
|  | LINC01203 |  |  |
|  | RNF144A-AS1 |  |  |
|  | HID1-AS1 |  |  |
|  | LINC01002 |  |  |
|  | LINC02168 |  |  |
|  | PSMD6-AS1 |  |  |
|  | SMCR2 |  |  |
|  | ARHGEF26-AS1 |  |  |
|  | LINC01350 |  |  |
|  | MMP25-AS1 |  |  |
|  | LINC00471 |  |  |
|  | BACH1-AS1 |  |  |
|  | LINC01152 |  |  |
|  | GYG2-AS1 |  |  |
|  | LINC01945 |  |  |
|  | IPO9-AS1 |  |  |
|  | HDHD5-AS1 |  |  |
|  | RASAL2-AS1 |  |  |
|  | LINC00385 |  |  |
|  | EVX1-AS |  |  |
|  | LINC01016 |  |  |
|  | LINC01820 |  |  |
|  | FAM222A-AS1 |  |  |
|  | PLCE1-AS1 |  |  |
|  | CATIP-AS2 |  |  |
|  | SH3TC2-DT |  |  |
|  | LINC02241 |  |  |
|  | MIR4307HG |  |  |
|  | LINC01641 |  |  |
|  | LINC01659 |  |  |
|  | LINC01310 |  |  |
|  | LINC02640 |  |  |
|  | OXCT1-AS1 |  |  |
|  | SPATA41 |  |  |
|  | DLX6-AS1 |  |  |
|  | SEMA3B-AS1 |  |  |
|  | LINC01076 |  |  |
|  | LINC2194 |  |  |
|  | LINC00676 |  |  |
|  | LINC01202 |  |  |
|  | LINC02585 |  |  |
|  | LINC00906 |  |  |
|  | FOCAD-AS1 |  |  |
|  | FAM242A |  |  |
|  | TRERNA1 |  |  |
|  | IQCJ-SCHIP1-AS1 |  |  |
|  | LINC01442 |  |  |
|  | RAET1E-AS1 |  |  |
|  | LINC01229 |  |  |
|  | CRTC3-AS1 |  |  |
|  | SIAH2-AS1 |  |  |
|  | LINC00381 |  |  |
|  | LINC02535 |  |  |
|  | LINC00678 |  |  |
|  | LINC01353 |  |  |
|  | A2M-AS1 |  |  |
|  | PAXBP1-AS1 |  |  |
|  | HTR2A-AS1 |  |  |
|  | MTUS2-AS2 |  |  |
|  | ID2-AS1 |  |  |
|  | BACE1-AS |  |  |
|  | LNCARSR |  |  |
|  | MAFTRR |  |  |
|  | RBM15-AS1 |  |  |
|  | LINC01798 |  |  |
|  | SNHG4 |  |  |
|  | LINC01619 |  |  |
|  | EHMT2-AS1 |  |  |
|  | MIS18A-AS1 |  |  |
|  | CAHM |  |  |
|  | FBXO3-DT |  |  |
|  | IL12A-AS1 |  |  |
|  | STEAP3-AS1 |  |  |
|  | LINC02145 |  |  |
|  | LINC02437 |  |  |
|  | TTTY5 |  |  |
|  | LINC01693 |  |  |
|  | GAS5-AS1 |  |  |
|  | C9orf135-DT |  |  |
|  | LINC00308 |  |  |
|  | THSD4-AS1 |  |  |
|  | LHX1-DT |  |  |
|  | LINC01637 |  |  |
|  | GIHCG |  |  |
|  | LINC02454 |  |  |
|  | NDUFV2-AS1 |  |  |
|  | LINC00489 |  |  |
|  | EPCAM-DT |  |  |
|  | LINC00322 |  |  |
|  | LUCAT1 |  |  |
|  | LINC00173 |  |  |
|  | LINC01179 |  |  |
|  | LINC00649 |  |  |
|  | LINC02134 |  |  |
|  | LINC00315 |  |  |
|  | LINC02517 |  |  |
|  | SNHG12 |  |  |
|  | P3H2-AS1 |  |  |
|  | LINC00114 |  |  |
|  | LINC02301 |  |  |
|  | CXXC4-AS1 |  |  |
|  | PSPC1-AS2 |  |  |
|  | LINC02200 |  |  |
|  | LINC01187 |  |  |
|  | LINC00623 |  |  |
|  | DACT3-AS1 |  |  |
|  | DPYD-AS1 |  |  |
|  | DLGAP1-AS1 |  |  |
|  | LINC01562 |  |  |
|  | EIF2AK3-DT |  |  |
|  | LINC02654 |  |  |
|  | LINC00500 |  |  |
|  | HCG18 |  |  |
