# Supplementary material for: Long non-coding RNA linc00921 suppresses tumorigenesis and epithelial-to-mesenchymal transition of triple-negative breast cancer via targeting miR-9-5p/LZTS2 axis
Source: Hum Cell. 2022 Feb 18;35(3):909–23. doi: 10.1007/s13577-022-00685-6 (PMC9013323; doi:10.1007/s13577-022-00685-6)
Supplement: Supplementary file 6 — Supplementary file6 (DOCX 14 KB) [file 13577_2022_685_MOESM6_ESM.docx]

**Supplementary Table 4 Overlapped lncRNAs of GSE119233 and GSE115275**

| **Overlapped downregulated genes** | **Overlapped upregulated genes** |
| --- | --- |
| TRHDE-AS1 | LINC01705 |
| MIR4453HG | LINC01929 |
| PGM5-AS1 | ATP2A1-AS1 |
| RBPMS-AS1 | LINC00460 |
| FGF13-AS1 | LINC01655 |
| LINC01985 | SCAT1 |
| HOXA-AS2 | OGFRP1 |
| LINC02511 | GAPLINC |
| PGM5P4-AS1 | FOXD3-AS1 |
| WDFY3-AS2 | LEF1-AS1 |
| LYPLAL1-AS1 | LINC02280 |
| LRRC8C-DT | ELF3-AS1 |
| NR2F1-AS1 | LINC01388 |
| MAGI2-AS3 | LINC00205 |
| HOTAIRM1 | LINC02544 |
| HHIP-AS1 | LINC01389 |
| LINC01091 | LINC00539 |
| MAST4-AS1 | TMEM92-AS1 |
| LINC01412 | LINC01048 |
| NKAIN3-IT1 | LINC01771 |
| EIF1B-AS1 | MIR200CHG |
| INKA2-AS1 | LINC02317 |
| KCNJ2-AS1 | PVT1 |
| LINC00377 | CT62 |
| LINC00667 | SGO1-AS1 |
| RERE-AS1 | HMGA1P4 |
| BDNF-AS | LINC00052 |
|  | RNF144A-AS1 |
|  | MMP25-AS1 |
