# Supplementary material for: Long non-coding RNA linc00921 suppresses tumorigenesis and epithelial-to-mesenchymal transition of triple-negative breast cancer via targeting miR-9-5p/LZTS2 axis
Source: Hum Cell. 2022 Feb 18;35(3):909–23. doi: 10.1007/s13577-022-00685-6 (PMC9013323; doi:10.1007/s13577-022-00685-6)
Supplement: Supplementary file 7 — Supplementary file7 (DOCX 90 KB) [file 13577_2022_685_MOESM7_ESM.docx]

| **miRmap** | **microT** | **miRanda** | **PicTar** | **TargetScan** | **PITA** |
| --- | --- | --- | --- | --- | --- |
| ACOT7 | CEP104 | ICMT | ICMT | ICMT | ICMT |
| KLHL21 | ICMT | ACOT7 | ACOT7 | ACOT7 | ACOT7 |
| EMC1 | KIAA2013 | IFFO2 | IFFO2 | IFFO2 | KLHL21 |
| LUZP1 | SRSF10 | MUL1 | STMN1 | SRSF10 | EPHA2 |
| SRSF10 | STMN1 | GALE | AHDC1 | STMN1 | RCC2 |
| WASF2 | SLC9A1 | RSRP1 | DNAJC8 | SLC9A1 | EMC1 |
| MYCBP | WASF2 | STMN1 | SNIP1 | AHDC1 | MUL1 |
| ANKRD13 | DNAJC8 | SLC9A1 | MYCBP | SNIP1 | HP1BP3 |
| KYAT3 | MYCBP | AHDC1 | GPBP1L1 | MYCBP | LUZP1 |
| FRRS1 | PABPC4 | TRAPPC3 | PIK3R3 | GPBP1L1 | ZNF436 |
| CLCC1 | BMP8B | SNIP1 | DBT | PIK3R3 | GALE |
| ARNT | GPBP1L1 | YRDC | SLC25A24 | SERBP1 | SYF2 |
| UBE2Q1 | PIK3R3 | MED8 | NOTCH2 | GCLM | RSRP1 |
| ADAR | MKNK1 | GPBP1L1 | UBE2Q1 | SASS6 | STMN1 |
| PYGO2 | CC2D1B | PIK3R3 | SHC1 | SLC25A24 | PAFAH2 |
| SHC1 | USP24 | JUN | SLC19A2 | NOTCH2 | SLC9A1 |
| TADA1 | DOCK7 | SLC44A5 | SCYL3 | OTUD7B | AHDC1 |
| SCYL3 | JAK1 | PIGK | RC3H1 | CERS2 | DNAJC8 |
| DSTYK | DEPDC1 | GCLM | ABL2 | UBE2Q1 | PHC2 |
| SLC30A1 | AC118549.1 | SASS6 | ZBTB41 | SHC1 | SFPQ |
| PTPN14 | SSX2IP | SLC25A24 | PHF13 | SLC19A2 | TRAPPC3 |
| NUP133 | KYAT3 | ST7L | VAMP3 | SCYL3 | SNIP1 |
| C1orf198 | GCLM | NOTCH2 | LDLRAP1 | RC3H1 | C1orf109 |
| TOMM20 | FRRS1 | OTUD7B | ARID1A | ABL2 | MTF1 |
| LYST | SASS6 | CERS2 | ZNF362 | ZBTB41 | MYCBP |
| NID1 | VAV3 | JTB | RNF11 | PTPN14 | ELOVL1 |
| CHML | SLC25A24 | UBE2Q1 | NFIA | PHF13 | MED8 |
| PHF13 | ST7L | SHC1 | AK4 | VAMP3 | SLC6A9 |
| CLCN6 | DENND2C | PIGM | SH3GLB1 | ALPL | GPBP1L1 |
| OTUD3 | NOTCH2 | TADA1 | DR1 | PNRC2 | PIK3R3 |
| NBPF3 | OTUD7B | CREG1 | SNX7 | CLIC4 | POMGNT1 |
| NIPAL3 | ARNT | SLC19A2 | NTNG1 | LDLRAP1 | MKNK1 |
| LDLRAP1 | CERS2 | SCYL3 | CELSR2 | ARID1A | EFCAB14 |
| PDIK1L | UBE2Q1 | PTGS2 | CAPZA1 | ZNF362 | SSBP3 |
| ARID1A | SHC1 | ELK4 | VANGL1 | ZC3H12A | DHCR24 |
| TINAGL1 | ASH1L | PARP1 | MAN1A2 | CAP1 | JUN |
| PPCS | PIGM | NUP133 | FAM46C | RNF11 | SERBP1 |
| BTF3L4 | SLC19A2 | C1orf198 | PRUNE1 | NFIA | GNG12 |
| SCP2 | SCYL3 | VAMP3 | ATP8B2 | AK4 | KYAT3 |
| LRRC8C | RC3H1 | KIF1B | EFNA1 | SH3GLB1 | GBP3 |
| MTF2 | ABL2 | DNAJC16 | SLC50A1 | DR1 | TGFBR3 |
| SNX7 | STX6 | NBPF3 | LMNA | SNX7 | GCLM |
| NTNG1 | EDEM3 | ALPL | ATP1B1 | NTNG1 | F3 |
| CAPZA1 | KIF14 | SRRM1 | CDC73 | CAPZA1 | DBT |
| HIPK1 | SLC30A1 | LDLRAP1 | BTG2 | VANGL1 | SLC25A24 |
| VANGL1 | PTPN14 | PDIK1L | FBXO28 | MAN1A2 | CLCC1 |
| MAB21L3 | TOMM20 | ARID1A | FAM107B | FAM46C | WDR47 |
| MAN1A2 | NID1 | PPP1R8 | ABI1 | PRUNE1 | ST7L |
| FAM46C | PHF13 | EPB41 | NRP1 | ATP8B2 | RHOC |
| NOTCH2N | VAMP3 | TINAGL1 | PARG | ZBTB7B | IGSF3 |
| PRUNE1 | UBE4B | ZC3H12A | SGMS1 | EFNA1 | NOTCH2 |
| ATP8B2 | VPS13D | CDCA8 | FAM13C | SLC50A1 | PRKAB2 |
| IL6R | SZRD1 | MMACHC | CCDC6 | KIRREL1 | HIST2H2BF |
| SLC50A1 | OTUD3 | SCP2 | CPEB3 | POU2F1 | HIST2H2BE |
| LMNA | ALPL | ALG6 | TM9SF3 | ATP1B1 | OTUD7B |
| UHMK1 | NIPAL3 | TYW3 | GPAM | ZBTB37 | ENSA |
| ATP1B1 | LDLRAP1 | ST6GALN | KIAA1217 | CDC73 | ARNT |

**Supplementary Table 5 Predicted target mRNAs of miR-9-5p**

| HMCN1 | ARID1A | PRKACB | BICC1 | BTG2 | CERS2 |
| --- | --- | --- | --- | --- | --- |
| NAV1 | TINAGL1 | DR1 | SIRT1 | CD55 | S100A10 |
| ATP2B4 | S100PBP | SNX7 | LZTS2 | FBXO28 | S100A16 |
| MDM4 | ZNF362 | NTNG1 | SFXN2 | CNIH4 | JTB |
| CD46 | ZC3H12A | MAB21L3 | BTBD10 | FAM107B | C1orf43 |
| RCOR3 | MACF1 | MAN1A2 | PIK3C2A | ABI1 | UBE2Q1 |
| ATF3 | ZFP69B | FAM46C | PHF21A | NRP1 | ADAR |
| CNIH4 | PPCS | FAM72B | AMBRA1 | PARG | PYGO2 |
| EDARAD | RNF11 | PRUNE1 | CELF1 | TIMM23 | SHC1 |
| LGALS8 | PATJ | ATP8B2 | SHANK2 | SGMS1 | ASH1L |
| USP6NL | LEPR | SLC50A1 | ETS1 | FAM13C | KHDC4 |
| FAM107B | TYW3 | POU2F1 | RASSF10 | CCDC6 | SSR2 |
| NSUN6 | ADGRL2 | ATP1B1 | LDLRAD3 | CPEB3 | DCAF8 |
| MKX | LRRC8D | SOAT1 | PTPRJ | TM9SF3 | TADA1 |
| JCAD | AC093423.3 | HMCN1 | ZDHHC5 | GPAM | CREG1 |
| NRP1 | MTF2 | CDC73 | TMEM109 | MCMBP | SLC19A2 |
| FZD8 | DR1 | FAM72A | RPS6KA4 | IKZF5 | SCYL3 |
| PARG | SNX7 | CD55 | KDM2A | KIAA1217 | RC3H1 |
| TIMM23 | MFSD14A | NENF | ARHGEF1 | BICC1 | ABL2 |
| FAM13C | AC118553.2 | RPS6KC1 | YAP1 | SIRT1 | GLUL |
| CCDC6 | CAPZA1 | LYPLAL1 | ARCN1 | SAMD8 | EDEM3 |
| RHOBTB1 | HIPK1 | SPRTN | M6PR | TNKS2 | PTGS2 |
| LIPA | VANGL1 | TSNAX | YBX3 | CCNJ | ZBTB41 |
| CPEB3 | MAB21L3 | TSNAX-D | KIF21A | TWNK | CSRP1 |
| GOT1 | PTGFRN | LGALS8 | SENP1 | LZTS2 | PPP1R15B |
| CELF2 | MAN1A2 | CNST | CCNT1 | SFXN2 | PIK3C2B |
| PTER | FAM46C | FAM107B | ANKRD52 | DUSP8 | DSTYK |
| TFAM | RPRD2 | TRDMT1 | B4GALNT | BTBD10 | ELK4 |
| BICC1 | PRUNE1 | NSUN6 | CTDSP2 | PIK3C2A | RAB29 |
| SIRT1 | ATP8B2 | ABI1 | DUSP6 | PHF21A | YOD1 |
| SGPL1 | EFNA1 | MKX | ARL1 | AMBRA1 | LPGAT1 |
| SAMD8 | SLC50A1 | NRP1 | NCOR2 | MTMR2 | LBR |
| ENTPD1 | LMNA | ZNF33B | STK38L | ALG9 | PARP1 |
| LZTS2 | POU2F1 | CXCL12 | ASIC1 | ETS1 | TRIM11 |
| SFXN2 | ATP1B1 | PARG | ATF1 | PDE3B | NUP133 |
| PLEKHA1 | BLZF1 | TIMM23 | ACVR1B | LDLRAD3 | ABCB10 |
| ACADSB | SOAT1 | FAM13C | RAB5B | PTPRJ | C1orf198 |
| PIK3C2A | CEP350 | CCDC6 | IKZF4 | ZDHHC5 | TOMM20 |
| HPS5 | HMCN1 | MICU1 | ZC3H10 | TMEM109 | LYST |
| NXF1 | CDC73 | PPP3CB | ESYT1 | RPS6KA4 | NID1 |
| MRE11 | MAPKAPK2 | ACTA2 | RASSF3 | KDM2A | CHML |
| MTMR2 | CD46 | CPEB3 | HMGA2 | ARHGEF17 | AKT3 |
| ALKBH8 | ATF3 | IDE | DYRK2 | YAP1 | B3GALT6 |
| BACE1 | RPS6KC1 | GPAM | RAP1B | ARCN1 | PHF13 |
| SNX19 | CNIH4 | CCDC186 | HCFC2 | M6PR | VAMP3 |
| TEAD1 | TSNAX | TIAL1 | SH2B3 | YBX3 | CLCN6 |
| C11orf58 | TSNAX-DISC1 | MCMBP | ZDHHC20 | PLBD1 | MFN2 |
| LDHA | GGPS1 | IKZF5 | SACS | TMTC1 | NECAP2 |
| CD44 | LGALS8 | MLLT10 | FLT1 | KIF21A | OTUD3 |
| TRIM44 | MTR | KIAA1217 | SPART | SENP1 | NBPF3 |
| LDLRAD3 | CHRM3 | MAP3K8 | ELF1 | CCNT1 | ALPL |
| UVRAG | FAM107B | BICC1 | KLF12 | ANKRD52 | PNRC2 |
| YAP1 | TRDMT1 | SIRT1 | FBXL3 | B4GALNT1 | NIPAL3 |
| ATP5MG | ABI1 | H2AFY2 | DOCK9 | CTDSP2 | CLIC4 |
| ARHGEF1 | MKX | PAPSS2 | ZIC5 | LIN7A | LDLRAP1 |
| KDM5A | NRP1 | LGI1 | FRY | ARL1 | SELENON |
| M6PR | FZD8 | CCNJ | FREM2 | PPTC7 | PDIK1L |
| YBX3 | PARG | CUTC | LACC1 | SBNO1 | SH3BGRL3 |

| PLBD1 | TIMM23 | TWNK | KLF5 | NCOR2 | ARID1A |
| --- | --- | --- | --- | --- | --- |
| IPO8 | SGMS1 | LZTS2 | DNAJC3 | STK38L | SFN |
| AMIGO2 | FAM13C | PPRC1 | RAP2A | ASIC1 | PHACTR4 |
| SENP1 | CCDC6 | SFXN2 | COL4A2 | ATF1 | TINAGL1 |
| CCNT1 | ANK3 | VTI1A | ATP11A | ACVR1B | KPNA6 |
| CBX5 | RHOBTB1 | WDR11 | CUL4A | COPZ1 | TXLNA |
| ZBTB39 | JMJD1C | ZNF511 | LAMP1 | RAB5B | KIAA1522 |
| NAP1L1 | MICU1 | ZNF511-P | AJUBA | IKZF4 | ZNF362 |
| KITLG | CPEB3 | BTBD10 | IPO4 | ZC3H10 | ZC3H12A |
| DUSP6 | CCDC186 | PIK3C2A | EGLN3 | ESYT1 | CAP1 |
| ARL1 | ABLIM1 | HPS5 | SPTSSA | RASSF3 | ZMPSTE24 |
| NUAK1 | SHTN1 | EHD1 | FBXO33 | HMGA2 | SZT2 |
| GIT2 | MCMBP | CTSC | NID2 | DYRK2 | MMACHC |
| PPP1CC | IKZF5 | MTMR2 | GNPNAT1 | RAP1B | NSUN4 |
| NCOR2 | CHST15 | ALKBH8 | ATG14 | HCFC2 | CMPK1 |
| ANO6 | PFKFB3 | SLC35F2 | SIX4 | SH2B3 | RNF11 |
| ATF1 | PTER | CTR9 | KCNH5 | SUDS3 | BTF3L4 |
| METTL7A | CACNB2 | PDE3B | NEK9 | ZDHHC20 | SCP2 |
| IKZF4 | KIAA1217 | METTL15 | FOXN3 | SACS | PCSK9 |
| ESYT1 | ZNF33A | TMEM109 | BTBD7 | SLC7A1 | ALG6 |
| SRGAP1 | BICC1 | TMEM138 | FOXG1 | SPART | AK4 |
| HMGA2 | REEP3 | RPS6KA4 | ARF6 | KLF12 | SH3GLB1 |
| MDM2 | SIRT1 | ARHGEF1 | FRMD6 | FBXL3 | PKN2 |
| NUDT4 | VCL | MRPL48 | PPM1A | DOCK9 | DR1 |
| MRPL42 | KAT6B | C11orf54 | PCNX1 | ZIC5 | FNBP1L |
| PARPBP | SAMD8 | ATP5MG | PSEN1 | NUP58 | SNX7 |
| OAS1 | STAMBPL1 | NLRX1 | GSKIP | FRY | PLPPR4 |
| FBXW8 | LCOR | TBCEL | RCOR1 | FREM2 | S1PR1 |
| SUDS3 | TWNK | M6PR | EHD4 | COG3 | NTNG1 |
| GATC | LZTS2 | YBX3 | UBR1 | KLF5 | CELSR2 |
| UNC119B | SFXN2 | PLBD1 | PPIP5K1 | DNAJC3 | CAPZA1 |
| DENR | NHLRC2 | SINHCAF | FBN1 | RAP2A | HIPK1 |
| EP400 | AL162407.1 | KIF21A | TRPM7 | COL4A2 | VANGL1 |
| SACS | TRUB1 | SENP1 | ONECUT1 | ATP11A | MAB21L3 |
| SHISA2 | SEC23IP | LRIG3 | NEDD4 | CUL4A | PTGFRN |
| STARD13 | PLEKHA1 | ARL1 | ZNF280D | LAMP1 | MAN1A2 |
| FOXO1 | ACADSB | PPP1CC | ADAM10 | AJUBA | FAM46C |
| EPSTI1 | TRIM66 | C12orf49 | ANXA2 | IPO4 | FAM72B |
| LCP1 | BTBD10 | NCOR2 | RORA | HECTD1 | NOTCH2NL |
| TBC1D4 | HPS5 | POLE | ADPGK | EGLN3 | TXNIP |
| FBXL3 | PHF21A | STK38L | SIN3A | SPTSSA | SETDB1 |
| DOCK9 | AMBRA1 | ATF1 | CHSY1 | FBXO33 | PRUNE1 |
| FREM2 | CKAP5 | KRT18 | PAK6 | NID2 | CGN |
| DNAJC15 | AHNAK | IKZF4 | BUB1B-PA | GNPNAT1 | ATP8B2 |
| COG3 | MAP3K11 | ZC3H10 | SNAP23 | ATG14 | IL6R |
| KLF5 | SHANK2 | ESYT1 | SEMA6D | SIX4 | ZBTB7B |
| CUL4A | FAM76B | HMGA2 | RNF111 | KCNH5 | EFNA1 |
| LAMP1 | MTMR2 | DYRK2 | ARID3B | FOXN3 | SLC50A1 |
| IPO4 | ALKBH8 | RAP1B | HMG20A | TC2N | LMNA |
| VCPKMT | HSPA8 | TMTC3 | DNAJA4 | BTBD7 | ATP1B1 |
| NIN | ETS1 | VEZT | TLNRD1 | DICER1 | PRRC2C |
| NID2 | NCAPD3 | TMPO | ZSCAN2 | HSP90AA1 | XPR1 |
| GNPNAT1 | C11orf58 | PARPBP | FURIN | FOXG1 | LAMC1 |
| KCNH5 | KCNC1 | HCFC2 | FBXL16 | PNN | RGL1 |
| NUMB | HIPK3 | SH2B3 | UNKL | ARF6 | C1orf21 |
| NEK9 | TRIM44 | OAS3 | ADCY9 | FRMD6 | HMCN1 |
| TMED8 | LDLRAD3 | SUDS3 | NAGPA | PPM1A | CDC73 |
| SPTLC2 | ZDHHC5 | SACS | DYNC1LI2 | PSEN1 | NAV1 |

| TC2N | FAM111B | SLC46A3 | ZFHX3 | GSKIP | BTG2 |
| --- | --- | --- | --- | --- | --- |
| ATXN3 | TMEM109 | UBL3 | ZDHHC7 | RCOR1 | ATP2B4 |
| RNASE4 | PPP6R3 | SPART | UBN1 | EHD4 | ZC3H11A |
| AL163636. | ARHGEF17 | ELF1 | MKL2 | TTBK2 | ZBED6 |
| FRMD6 | AMOTL1 | MED4 | FBXL19 | PPIP5K1 | CDK18 |
| OTUB2 | ENDOD1 | RCBTB1 | CHD9 | FBN1 | MFSD4A |
| UBE3A | ATM | FBXL3 | MMP15 | TRPM7 | MAPKAPK2 |
| UBR1 | FDX1 | DOCK9 | C16orf70 | ONECUT1 | CD46 |
| FBN1 | SIK2 | ZIC5 | NUTF2 | NEDD4 | RCOR3 |
| TRPM7 | DIXDC1 | COL4A1 | MYO1C | ZNF280D | ATF3 |
| ONECUT1 | DLAT | FRY | NCBP3 | ADAM10 | RPS6KC1 |
| RAB27A | REXO2 | RFC3 | ZBTB4 | ANXA2 | PROX1 |
| PRTG | KMT2A | FREM2 | PMP22 | ADPGK | RRP15 |
| NEDD4 | ARCN1 | LACC1 | ULK2 | SIN3A | CAPN2 |
| ZNF280D | KDM5A | GTF2F2 | RAB34 | CHSY1 | IBA57 |
| RORA | DCP1B | COG3 | CBX1 | PAK6 | GALNT2 |
| SIN3A | M6PR | LRCH1 | FAM117A | BUB1B-PAK6 | SPRTN |
| CHP1 | YBX3 | KLF5 | ANKRD40 | SNAP23 | MAP3K21 |
| SNAP23 | TMTC1 | DNAJC3 | SPAG9 | SEMA6D | GPR137B |
| TMOD3 | CAPRIN2 | MBNL2 | APPBP2 | MINDY2 | EDARADD |
| TLNRD1 | SINHCAF | COL4A2 | SMARCD2 | RNF111 | LGALS8 |
| FURIN | VDR | CUL4A | ARHGDIA | PAQR5 | CNST |
| CHD2 | SENP1 | LAMP1 | RAB40B | ARID3B | KLF6 |
| SEPHS2 | KANSL2 | IPO4 | KIF1C | DNAJA4 | IL15RA |
| CNOT1 | CCNT1 | SPTSSA | MAP2K3 | TLNRD1 | USP6NL |
| CMTR2 | TUBA1B | NIN | CALCOCO | FURIN | FAM107B |
| ZFHX3 | TUBA1A | NID2 | UBE2Z | UNKL | TRDMT1 |
| ZCCHC14 | CBX5 | GNPNAT1 | IGF2BP1 | ADCY9 | NSUN6 |
| JPT2 | ANKRD52 | GCH1 | TANC2 | DYNC1LI2 | ABI1 |
| MKL2 | ZBTB39 | ATG14 | MAP3K3 | CMTR2 | ACBD5 |
| ITPRIPL2 | STAT6 | SIX4 | PITPNC1 | MBTPS1 | MKX |
| IQCK | CTDSP2 | PLEK2 | KCNJ2 | ZDHHC7 | JCAD |
| METTL9 | LRIG3 | NUMB | COLEC12 | NME4 | NRP1 |
| EEF2K | LLPH | NEK9 | TAF4B | JPT2 | FZD8 |
| PRKCB | AC078927.1 | TC2N | GALNT1 | UBN1 | ZNF33B |
| C16orf70 | KRR1 | DICER1 | C18orf25 | MKL2 | CXCL12 |
| SNTB2 | NAP1L1 | HSP90AA | SMAD4 | UBFD1 | PARG |
| NIP7 | OSBPL8 | RNASE4 | KIAA1468 | FBXL19 | TIMM23 |
| WWP2 | LIN7A | AL163636. | DNAJB1 | MMP15 | SGMS1 |
| SHMT1 | KITLG | OXA1L | BRD4 | NUTF2 | FAM13C |
| ZNF286B | DUSP6 | FOXG1 | DYRK1B | MYO1C | SLC16A9 |
| PIGS | ARL1 | G2E3 | POU2F2 | NCBP3 | CCDC6 |
| UTP6 | SSH1 | C14orf28 | BCAT2 | ZBTB4 | RHOBTB1 |
| TNS4 | GIT2 | ARF6 | ZNF615 | SHMT1 | DNA2 |
| SMARCE1 | PPTC7 | SLC39A9 | PTBP1 | ULK2 | DNAJB12 |
| AC073508. | CLIP1 | RIOX1 | UHRF1 | SMARCE1 | MICU1 |
| RETREG3 | RSRC2 | ZC3H14 | CARM1 | AC073508.2 | AP3M1 |
| MPP3 | MPHOSPH9 | GSKIP | RAB8A | VAT1 | GLUD1 |
| ANKRD40 | SBNO1 | UBE3A | LSM14A | MPP3 | ACTA2 |
| SPAG9 | NCOR2 | SRP14 | PLD3 | GJC1 | CPEB3 |
| COX11 | POLE | OIP5 | AXL | FAM117A | IDE |
| TRIM25 | AEBP2 | EHD4 | SDC1 | ANKRD40 | BLNK |
| CYB561 | STK38L | FBN1 | ATL2 | SPAG9 | TM9SF3 |
| SMARCD2 | PPFIBP1 | TRPM7 | SOS1 | APPBP2 | MMS19 |
| HELZ | KLHL42 | ONECUT1 | BCL11A | SMARCD2 | GOT1 |
| ZNF286A | CACNB3 | NEDD4 | BIN1 | H3F3B | COX15 |
| AC005324. | TUBA1C | ZNF280D | MAP3K2 | AATK | ERLIN1 |
| SPECC1 | METTL7A | ADAM10 | BAZ2B | NPLOC4 | ACTR1A |

| TMEM199 | ACVR1B | ANXA2 | DPP4 | ARHGDIA | SH3PXD2A |
| --- | --- | --- | --- | --- | --- |
| TMEM106 | GDF11 | PIF1 | ATF2 | HIC1 | ITPRIP |
| CALCOCO | RAB5B | SIN3A | HECW2 | KIF1C | GPAM |
| KAT7 | ZC3H10 | SEC11A | PGAP1 | MAP2K3 | CCDC186 |
| TANC2 | ESYT1 | LYSMD4 | FAM126B | CALCOCO2 | ABLIM1 |
| MAP3K3 | TSFM | KNL1 | KLF7 | UBE2Z | SHTN1 |
| KCNJ2 | AC025165.3 | RAD51 | IKZF2 | MAP3K3 | MCMBP |
| CBX2 | RASSF3 | AC012476. | OBSL1 | PITPNC1 | FAM53B |
| SLC25A10 | DYRK2 | AP4E1 | HDAC4 | KCNJ2 | CTBP2 |
| AC139530. | RAP1B | RNF111 | CRIM1 | KCTD2 | EBF3 |
| COLEC12 | SYT1 | ARID3B | EPAS1 | ITGB4 | CELF2 |
| FAM210A | NEDD1 | C15orf39 | SOCS5 | SYNGR2 | PTER |
| ZNF24 | SH2B3 | IDH3A | LGALSL | COLEC12 | ARL5B |
| MEX3C | OAS1 | DNAJA4 | RMND5A | TMEM200C | KIAA1217 |
| TMX3 | SUDS3 | IREB2 | BCL2L11 | USP14 | MAP3K8 |
| NAPG | EP400 | MORF4L1 | TMEM87B | TAF4B | CREM |
| GNAL | XPO4 | TLNRD1 | CCNT2 | GALNT1 | ZNF33A |
| TAF4B | ZDHHC20 | ZSCAN2 | GAD1 | C18orf25 | TFAM |
| SKA1 | SACS | PDE8A | ITGA6 | KIAA1468 | BICC1 |
| ZNF585B | SHISA2 | IQGAP1 | AGFG1 | ZBTB7A | ARID5B |
| ONECUT3 | RNF6 | FURIN | SH3BP4 | DNAJB1 | SIRT1 |
| C19orf66 | SPART | ASB7 | RNF24 | BRD4 | COL13A1 |
| RAB8A | FOXO1 | TMEM8A | HM13 | DYRK1B | SGPL1 |
| GTPBP3 | ZC3H13 | MARF1 | SRSF6 | POU2F2 | CHST3 |
| KCTD15 | TBC1D4 | GDE1 | HSPA13 | BCAT2 | VCL |
| LSM14A | KCTD12 | DYNC1LI2 | C21orf91 | PTBP1 | TMEM254 |
| AXL | FBXL3 | NOB1 | ADAMTS5 | ONECUT3 | BMPR1A |
| ZNF230 | MYCBP2 | CMTR2 | RUNX1 | CSNK1G2 | PAPSS2 |
| CYTH2 | CDK8 | NME4 | ERG | DOT1L | IFIT2 |
| ZNF71 | FREM2 | JPT2 | SIK1 | CARM1 | TNKS2 |
| PRKD3 | DNAJC15 | TEDC2 | SIK1B | RAB8A | 44260 |
| ATL2 | COG3 | MKL2 | SLC5A3 | GTPBP3 | KIF11 |
| BCL11A | ITM2B | ZNF720 | DYRK1A | LSM14A | ENTPD1 |
| C1D | KLF5 | CHD9 | COL18A1 | PXDN | CCNJ |
| MOB1A | LMO7 | C16orf70 | ZNF280B | SDC1 | SCD |
| SLC35F5 | MBNL2 | NUTF2 | THOC5 | SLC5A6 | HIF1AN |
| CXCR4 | ATP11A | WWP2 | RBFOX2 | ATL2 | LZTS2 |
| GALNT3 | CUL4A | MYO1C | DDX17 | SOS1 | SFXN2 |
| TLK1 | ZFHX2 | PITPNA | CBX6 | EFEMP1 | PDCD4 |
| LNPK | IPO4 | PLSCR3 | HIC2 | BCL11A | VTI1A |
| NEMP2 | HECTD1 | TMEM256 | FAM118A | DCTN1 | FAM160B1 |
| RAPH1 | HEATR5A | ALOXE3 | VGLL4 | SMPD4 | PLEKHA1 |
| FZD5 | AL139353.1 | PMP22 | CMTM6 | BAZ2B | HTRA1 |
| MREG | SPTSSA | SHMT1 | FYCO1 | ATF2 | ACADSB |
| TNS1 | FBXO33 | PIGS | PBRM1 | HECW2 | DUSP8 |
| HDAC4 | SOS2 | JUP | FOXP1 | PGAP1 | IGF2 |
| PQLC3 | NIN | VAT1 | RYBP | FAM126B | NUP98 |
| EHD3 | NID2 | MPP3 | DCBLD2 | RAPH1 | TPP1 |
| SLC30A6 | GNPNAT1 | MAP3K14 | OSBPL11 | KLF7 | BTBD10 |
| PRKCE | DDHD1 | CBX1 | AMOTL2 | IKZF2 | PIK3C2A |
| SOCS5 | GCH1 | HOXB13 | PIK3CB | TNS1 | HPS5 |
| FOXN2 | ATG14 | FAM117A | SLC33A1 | PER2 | FANCF |
| MXD1 | SIX4 | SPAG9 | TBL1XR1 | CRIM1 | SLC5A12 |
| CCDC138 | KCNH5 | TRIM25 | TRA2B | EPAS1 | LIN7C |
| TMEM87B | NEK9 | CYB561 | BCL6 | SOCS5 | CD59 |
| STEAP3 | TMED8 | SMARCD2 | TFRC | LGALSL | PHF21A |
| CCNT2 | SPTLC2 | UBE2O | UBXN7 | RMND5A | AMBRA1 |
| LYPD6 | DIO2 | RAB40B | RBMS3 | CCDC138 | OSBP |

| FMNL2 | FOXN3 | HIC1 | PDCD6IP | BCL2L11 | PATL1 |
| --- | --- | --- | --- | --- | --- |
| CSRNP3 | CCDC88C | KIF1C | OXSR1 | TMEM87B | CPSF7 |
| CERS6 | ATXN3 | SLC2A4 | ACVR2B | CCNT2 | AHNAK |
| ITGA6 | FOXG1 | MYOCD | KLHL18 | GAD1 | NXF1 |
| TMEM169 | SCFD1 | WSB1 | PCNP | ITGA6 | STX5 |
| CNOT9 | TOGARAM1 | KSR1 | NXPE3 | AGFG1 | MEN1 |
| RHBDD1 | FRMD6 | CPD | ZBTB38 | SH3BP4 | EHD1 |
| TBC1D20 | RTRAF | SLFN5 | RNF7 | RNF24 | CDCA5 |
| TMEM230 | GPR137C | MRPL45 | TSC22D2 | HM13 | FOSL1 |
| NANP | NAA30 | CALCOCO | FNDC3B | HSPA13 | SHANK2 |
| SOGA1 | ARID4A | UBE2Z | AP2M1 | C21orf91 | FAM168A |
| NFATC2 | PPM1A | MAP3K3 | FYTTD1 | ADAMTS5 | PGM2L1 |
| BMP2 | SYNE2 | KCNJ2 | AFAP1 | CLDN14 | KCNE3 |
| RALGAPB | ZC3H14 | KCTD2 | FRYL | ERG | PRCP |
| SRSF6 | GSKIP | TSEN54 | GRSF1 | ZBTB21 | FZD4 |
| TTPAL | RCOR1 | ITGB4 | SEC31A | SIK1 | CTSC |
| ARFGEF2 | EIF5 | COLEC12 | PGRMC2 | SIK1B | SLC36A4 |
| PCMTD2 | CEP170B | ENOSF1 | PABPC4L | SLC5A3 | SMCO4 |
| C21orf91 | AQR | FAM210A | RNF150 | DYRK1A | MRE11 |
| CYYR1 | INO80 | GAREM1 | OTUD4 | COL18A1 | MTMR2 |
| LTN1 | EHD4 | USP14 | C4orf46 | THOC5 | MMP1 |
| RUNX1 | TTBK2 | TWSG1 | MFAP3L | RBFOX2 | SLC35F2 |
| CLDN14 | UBR1 | TAF4B | MRFAP1 | MYH9 | KDELC2 |
| ERG | PPIP5K1 | RNF138 | CPEB2 | DDX17 | RDX |
| BRWD1 | TRPM7 | C18orf25 | PCDH7 | CBX6 | PPP2R1B |
| ZBTB21 | MYO5A | KIAA1468 | SLAIN2 | MICALL1 | ALG9 |
| SLC5A3 | ONECUT1 | SERPINB8 | PCDH10 | GTPBP1 | AP001781.2 |
| MORC3 | PRTG | KANK2 | TRIM2 | FAM118A | BACE1 |
| TRAPPC1 | NEDD4 | DNAJB1 | SNX25 | PIM3 | MCAM |
| RBFOX2 | ADAM10 | BRD4 | GOLPH3 | VGLL4 | SRPRA |
| FOXRED2 | SLTM | SUGP2 | PRLR | CMTM6 | ETS1 |
| FAM118A | ANXA2 | ZNF43 | GDNF | FYCO1 | ADAMTS8 |
| PPARA | RORA | ZNF565 | MIER3 | RHOA | SNX19 |
| METTL6 | HERC1 | DYRK1B | ELOVL7 | PBRM1 | RASSF7 |
| SLC4A7 | PIF1 | POU2F2 | AP3B1 | EOGT | PNPLA2 |
| SUSD5 | CALML4 | TMEM143 | SERINC5 | FOXP1 | TSPAN4 |
| EPM2AIP1 | AC107871.1 | BCAT2 | PJA2 | RYBP | CTR9 |
| FYCO1 | UACA | PTBP1 | FBN2 | DCBLD2 | TEAD1 |
| SMARCC1 | ADPGK | NDUFS7 | FNIP1 | AMOTL2 | PDE3B |
| FOXP1 | PEAK1 | UHRF1 | P4HA2 | PIK3CB | C11orf58 |
| SHQ1 | POLG | ZNF557 | PDGFRB | SLC33A1 | LDHA |
| VGLL3 | CHSY1 | MAP2K7 | FBXW11 | EIF5A2 | TMEM86A |
| FSTL1 | KLF13 | C19orf66 | RNF44 | TBL1XR1 | ZDHHC13 |
| ISY1 | MGA | ILF3 | 6-Mar | BCL6 | NAV2 |
| TMCC1 | SNAP23 | MRI1 | ZNF131 | UBXN7 | METTL15 |
| XRN1 | CCNDBP1 | RAB8A | ISL1 | MKRN2 | CD44 |
| HLTF | MAP1A | GTPBP3 | SNX18 | RBMS3 | TRIM44 |
| EIF5A2 | SEMA6D | ZNF254 | MAP3K1 | TRIM71 | LDLRAD3 |
| PLD1 | USP8 | LSM14A | IPO11 | PDCD6IP | HSD17B12 |
| BCL6 | AP4E1 | AXL | VCAN | OXSR1 | MDK |
| TFRC | RNF111 | ZNF230 | POLR3G | SNRK | PTPRJ |
| SLC6A6 | NEO1 | PRMT1 | C5orf30 | KLHL18 | ZDHHC5 |
| RBMS3 | ARID3B | CNOT3 | TGFBI | WDR6 | CCDC86 |
| PDCD6IP | FBXO22 | ZNF530 | PURA | PTPRG | TMEM109 |
| NKTR | TLNRD1 | ZNF274 | CCNG1 | PCNP | TMEM138 |
| SNRK | ASB7 | PXDN | FGF18 | NXPE3 | FADS2 |
| LIMD1 | FBXL16 | SLC5A6 | CREBRF | ZBTB38 | RTN3 |
| SLMAP | UNKL | ATL2 | CPEB4 | RASA2 | RPS6KA4 |

| PTPRG | ADCY9 | SOS1 | FAF2 | RNF7 | KDM2A |
| --- | --- | --- | --- | --- | --- |
| PCNP | NTAN1 | EFEMP1 | TXNDC5 | TSC22D2 | ALDH3B1 |
| NXPE3 | SMG1 | BCL11A | BLOC1S5- | FXR1 | CCND1 |
| ALCAM | GDE1 | C1D | ATXN1 | AP2M1 | ARHGEF17 |
| SLC35A5 | SEPHS2 | DCTN1 | C6orf106 | LPP | MRPL48 |
| NUDT16 | CDH8 | KRCC1 | SRPK1 | HES1 | NEU3 |
| HPS3 | CDH11 | ANKRD39 | TRERF1 | FYTTD1 | SERPINH1 |
| IL12A | DYNC1LI2 | SEPT10 | TNFRSF21 | AFAP1 | UVRAG |
| FNDC3B | CMTR2 | BAZ2B | COL12A1 | FRYL | C11orf54 |
| FXR1 | ZFHX3 | DPP4 | EPHA7 | PPAT | AMOTL1 |
| CCDC50 | MBTPS1 | GALNT3 | CEP85L | GRSF1 | CEP57 |
| APBB2 | ZCCHC14 | HECW2 | PTPRK | CCNI | YAP1 |
| CXCL11 | ANKRD11 | FAM126B | BCLAF1 | PABPC4L | CUL5 |
| CNOT6L | UBN1 | RAPH1 | FOXF2 | RNF150 | ATM |
| LIN54 | MKL2 | IKZF2 | ID4 | LRBA | SIK2 |
| PABPC4L | ITPRIPL2 | FN1 | PPARD | C4orf46 | DIXDC1 |
| RNF150 | METTL9 | MREG | PPP2R5D | MFAP3L | ATP5MG |
| NEK1 | EEF2K | PQLC3 | SH3BGRL | PIGG | TMEM25 |
| MFAP3L | UBFD1 | CRIM1 | ANKRD6 | MRFAP1 | ARCN1 |
| NSD2 | TNRC6A | SOCS5 | FOXO3 | CPEB2 | NLRX1 |
| EVC | LCMT1 | LGALSL | TBPL1 | PCDH7 | CBL |
| CPEB2 | FBRS | RMND5A | MTHFD1L | SLAIN2 | ARHGEF12 |
| TMEM33 | ZNF720 | RGPD4 | QKI | SGMS2 | EI24 |
| SLAIN2 | HEATR3 | CCDC138 | IGF2BP3 | PCDH10 | TMEM45B |
| SRD5A3 | FTO | BCL2L11 | OSBPL3 | TRIM2 | APLP2 |
| REST | C16orf70 | TMEM87B | HOXA11 | SNX25 | JAM3 |
| THAP6 | NUTF2 | STEAP3 | IGFBP3 | ANKH | KDM5A |
| SGMS2 | NFATC3 | RAB3GAP | PDK4 | MYO10 | LPCAT3 |
| USP53 | CDH1 | R3HDM1 | GIGYF1 | GOLPH3 | SLC2A3 |
| FGF2 | SNTB2 | TANC1 | EPHB4 | PRLR | AICDA |
| LARP1B | CYB5B | GAD1 | ATXN7L1 | GDNF | M6PR |
| PCDH10 | NFAT5 | GULP1 | MTPN | MIER3 | YBX3 |
| ELMOD2 | MYO1C | PMS1 | CREB3L2 | SREK1IP1 | PLBD1 |
| TRIM2 | NCBP3 | ABI2 | HIPK2 | ZBED3 | TMTC1 |
| SNX25 | KRBA2 | DIS3L2 | GLCCI1 | AP3B1 | IPO8 |
| ANKH | AC135178.2 | DGKD | CCDC126 | SERINC5 | SINHCAF |
| PRLR | RPL26 | RNF24 | CREB5 | PJA2 | PKP2 |
| LIFR | MYH4 | TMEM230 | ZNRF2 | REEP5 | KIF21A |
| EMB | MYH1 | FERMT1 | BMPER | FBN2 | GXYLT1 |
| IL6ST | PMP22 | NAPB | DBNL | FNIP1 | SLC38A1 |
| PDE4D | ULK2 | NANP | TMEM248 | P4HA2 | AMIGO2 |
| ELOVL7 | RAB34 | ZCCHC3 | PHTF2 | ZCCHC10 | VDR |
| ENC1 | SSH2 | PANK2 | RUNDC3B | PDGFRB | SENP1 |
| F2RL2 | MED1 | XRN2 | ARPC1A | MGAT1 | ASB8 |
| AP3B1 | SMARCE1 | HM13 | AGFG2 | 6-Mar | ADCY6 |
| EFNA5 | AC073508.2 | STK4 | PRKAR2B | ZNF131 | ARF3 |
| MCC | JUP | PIGT | FOXP2 | ISL1 | KMT2D |
| FBN2 | VAT1 | MIS18A | NRF1 | MAP3K1 | FMNL3 |
| FNIP1 | GPATCH8 | CLDN14 | KLHDC10 | IPO11 | FIGNL2 |
| P4HA2 | GJC1 | ZBTB21 | EN2 | NLN | KRT80 |
| PPP2CA | CBX1 | DYRK1A | UBE3C | VCAN | SPRYD3 |
| DCTN4 | HOXB13 | COL18A1 | CNOT7 | POLR3G | ATF7 |
| GEMIN5 | FAM117A | RBFOX2 | PSD3 | C5orf30 | SMARCC2 |
| PANK3 | SPAG9 | MYH9 | REEP4 | TNFAIP8 | ANKRD52 |
| 6-Mar | HEATR6 | DDX17 | ZNF395 | TGFBI | ZBTB39 |
| RAI14 | SMARCD2 | CBX7 | FBXO16 | CTNNA1 | NEMP1 |
| CCDC152 | GNA13 | SPECC1L | KIF13B | PURA | STAT6 |
| GPX8 | HELZ | FBXO7 | SLC20A2 | CCNG1 | B4GALNT1 |

| JMY | AATK | VGLL4 | TRAM1 | NPM1 | CTDSP2 |
| --- | --- | --- | --- | --- | --- |
| ZFYVE16 | RAB40B | SATB1 | SNX16 | FGF18 | PPM1H |
| VCAN | KIF1C | CMTM6 | STK3 | CREBRF | CPM |
| PPIP5K2 | MYOCD | PRKAR2A | RNF19A | CPEB4 | NAP1L1 |
| FER | HS3ST3B1 | PBRM1 | UBR5 | FAF2 | OSBPL8 |
| TNFAIP8 | GOSR1 | SFMBT1 | NDRG1 | SLC22A23 | PPP1R12A |
| TGFBI | NF1 | EOGT | SCRIB | TXNDC5 | KITLG |
| CTNNA1 | DHX8 | FRMD4B | SLC39A14 | BLOC1S5-TXN | DUSP6 |
| CYSTM1 | NMT1 | FOXP1 | CCAR2 | ATXN1 | ARL1 |
| KIAA0141 | KPNB1 | RYBP | ADGRA2 | HIST1H4H | GNPTAB |
| RNF14 | UBE2Z | SHQ1 | BAG4 | C6orf106 | NUAK1 |
| SLC36A1 | YPEL2 | CBLB | CTHRC1 | SRPK1 | CORO1C |
| MFAP3 | DHX40 | TMEM39A | FAM91A1 | TNFRSF21 | KCTD10 |
| GALNT10 | TANC2 | FSTL1 | PHF20L1 | DST | GIT2 |
| CCNG1 | MAP3K3 | OSBPL11 | RANBP6 | EPHA7 | PPP1CC |
| HMMR | PITPNC1 | ISY1 | TLN1 | PTPRK | C12orf49 |
| NPM1 | KCNJ2 | HLTF | RNF38 | BCLAF1 | PXN |
| CPEB4 | ITGB4 | SLC33A1 | SHB | SERAC1 | RHOF |
| FAF2 | CBX2 | EIF5A2 | AUH | FAM8A1 | DIABLO |
| CNOT6 | RNF213 | TRA2B | ZNF367 | ID4 | AC048338.1 |
| TRERF1 | COLEC12 | BCL6 | ABCA1 | HIST1H2AE | PITPNM2 |
| MMS22L | TMEM200C | UBXN7 | PTBP3 | UHRF1BP1 | NCOR2 |
| GOPC | FAM210A | PIGZ | TNC | PPARD | SLC15A4 |
| CEP85L | ROCK1 | MELTF | FBXW2 | UBR2 | POLE |
| ECHDC1 | SYT4 | CNTN4 | SLC1A1 | PPP2R5D | ADIPOR2 |
| BCLAF1 | ARHGAP28 | PPARG | LURAP1L | SRF | TULP3 |
| SERAC1 | ANKRD12 | MKRN2 | UBE2R2 | SH3BGRL2 | RIMKLB |
| FAM8A1 | RALBP1 | RAB5A | CLTA | NUS1 | CDKN1B |
| ID4 | CHMP1B | RBMS3 | SPIN1 | RNF146 | GPRC5A |
| BTN2A1 | CEP192 | TGFBR2 | ANP32B | TBPL1 | EMP1 |
| HIST1H2A | GREB1L | CRTAP | COL15A1 | MTHFD1L | MED21 |
| ZSCAN16 | TAF4B | PDCD6IP | MRRF | QKI | STK38L |
| ANKRD6 | DSG2 | WDR48 | ZBTB34 | CYTH3 | KLHL42 |
| PRDM1 | C18orf25 | CTNNB1 | SLC27A4 | IGF2BP3 | ANO6 |
| MARCKS | MAPK4 | SNRK | ARX | OSBPL3 | ASIC1 |
| NUS1 | ONECUT2 | KLHL18 | PHF8 | HOXA11 | ATF1 |
| L3MBTL3 | KIAA1468 | WDR6 | ARMCX2 | INHBA | METTL7A |
| ADGRG6 | ZNF236 | SLMAP | AMMECR | IGFBP3 | ACVR1B |
| PHACTR2 | DNMT1 | RPP14 | ATP11C | PDK4 | KRT86 |
| SASH1 | KANK2 | HTD2 | SLITRK4 | ZNF394 | RAB5B |
| ARID1B | PRKACA | PCNP | SLC10A3 | GIGYF1 | IKZF4 |
| TWISTNB | WIZ | NXPE3 | SYAP1 | EPHB4 | ZC3H10 |
| HOXA1 | ZNF850 | FAIM | ZFX | CREB3L2 | ESYT1 |
| HOXA11 | DYRK1B | ZBTB38 | DDX3X | HIPK2 | PIP4K2C |
| KIAA0895 | POU2F2 | TSC22D2 | CLCN5 | GLCCI1 | SRGAP1 |
| IGFBP3 | BCAT2 | P2RY1 | FAM199X | CCDC126 | LEMD3 |
| TNS3 | ONECUT3 | IL12A | RNF128 | CREB5 | HMGA2 |
| HIP1 | NFIC | SMC4 | FMR1 | DBNL | DYRK2 |
| PDK4 | CHAF1A | MFN1 |  | RABGEF1 | RAP1B |
| SMURF1 | ZNF557 | FXR1 |  | AC027644.4 | MDM2 |
| CCDC71L | MAP2K7 | AP2M1 |  | TMEM248 | RAB3IP |
| BMT2 | CC2D1A | LPP |  | PHTF2 | THAP2 |
| POT1 | RAB8A | HES1 |  | RUNDC3B | TMEM19 |
| TMEM209 | LSM14A | FYTTD1 |  | ARPC1A | C12orf29 |
| SLC35B4 | PAK4 | MRFAP1L1 | | ZNF655 | NUDT4 |
| LMBR1 | PRMT1 | TAPT1 |  | ZKSCAN1 | MRPL42 |
| ZNRF2 | ZNF264 | FRYL |  | AGFG2 | VEZT |
| BMPER | ZNF530 | PPAT |  | FOXP2 | PARPBP |

| DBNL | PXDN | HOPX | MET | UBE3B |
| --- | --- | --- | --- | --- |
| KCTD7 | RNASEH1 | SEC31A | CALU | FAM216A |
| TMEM248 | KIDINS220 | EIF4E | NRF1 | SH2B3 |
| GNAI1 | SDC1 | SLC39A8 | KLHDC10 | OAS1 |
| RUNDC3B | APOB | CENPE | EN2 | OAS3 |
| RBM48 | ITSN2 | ELOVL6 | UBE3C | OAS2 |
| ZKSCAN1 | PPM1G | PGRMC2 | CNOT7 | FBXW8 |
| CUX1 | PRKD3 | PABPC4L | PSD3 | SUDS3 |
| CBLL1 | ATL2 | RNF150 | REEP4 | RNF10 |
| FOXP2 | SOS1 | LRBA | ZNF395 | UNC119B |
| TES | CCDC88A | C4orf46 | FBXO16 | ORAI1 |
| CAV2 | EFEMP1 | HMGB2 | KIF13B | DENR |
| CPA4 | MOB1A | PIGG | SLC20A2 | TMED2 |
| INSIG1 | LRRTM4 | MRFAP1 | PLAG1 | ULK1 |
| UBE3C | TGOLN2 | BLOC1S4 | PDE7A | EP400 |
| FAM167A | IMMT | CPEB2 | TRAM1 | SACS |
| MTUS1 | RGPD2 | PCDH7 | STK3 | SHISA2 |
| CA8 | BIN1 | EREG | RNF19A | SLC46A3 |
| ASPH | MAP3K2 | THAP6 | UBR5 | SLC7A1 |
| ZFAND1 | CXCR4 | USO1 | NDRG1 | STARD13 |
| SNX16 | NEB | BMP2K | SCRIB | SPART |
| STK3 | BAZ2B | SGMS2 | SLC39A14 | FOXO1 |
| YWHAZ | GALNT3 | USP53 | CCAR2 | LCP1 |
| SNTB1 | ATF2 | PCDH10 | ADGRA2 | MED4 |
| HAS2 | LNPK | ELMOD2 | BAG4 | RCBTB1 |
| SLA | PRKRA | TRIM2 | VPS13B | KLF12 |
| SCRIB | FKBP7 | FNIP2 | PHF20L1 | TBC1D4 |
| ERI1 | NEMP2 | RAPGEF2 | DENND3 | KCTD12 |
| CCAR2 | PGAP1 | SNX25 | ERMP1 | FBXL3 |
| ERLIN2 | RAPH1 | GOLPH3 | GBA2 | DOCK9 |
| PLEKHA2 | PLEKHM3 | MIER3 | RNF38 | ZIC5 |
| PI15 | IKZF2 | PLK2 | SHB | COL4A1 |
| ESRP1 | MREG | ENC1 | AUH | RASA3 |
| VPS13B | TNS1 | ZBED3 | ZNF367 | PAN3 |
| CTHRC1 | AP1S3 | AP3B1 | ABCA1 | MEDAG |
| DENND3 | ARL4C | TTC37 | PTBP3 | FRY |
| ERMP1 | PER2 | CHD1 | ALAD | RFC3 |
| BNC2 | PQLC3 | FBN2 | TNC | FREM2 |
| KLHL9 | WDR43 | FNIP1 | FBXW2 | WBP4 |
| GNE | EHD3 | P4HA2 | SLC1A1 | DNAJC15 |
| PTAR1 | SLC30A6 | AFF4 | LURAP1L | GTF2F2 |
| ALAD | CRIM1 | PDGFRB | CARD19 | COG3 |
| TNC | SOCS5 | GEMIN5 | ANP32B | RB1 |
| TSC1 | FOXN2 | RNF44 | COL15A1 | KLF5 |
| LURAP1L | MTHFD2 | MGAT1 | TGFBR1 | HS6ST3 |
| MTAP | RMND5A | 6-Mar | SLC27A4 | RAP2A |
| DCAF10 | RGPD1 | C5orf22 | AP1S2 | ZIC2 |
| COL15A1 | RGPD4 | CCDC152 | ARX | COL4A2 |
| TMEM268 | GCC2 | ZNF131 | PHF8 | ATP11A |
| AP1S2 | RANBP2 | ISL1 | ARMCX2 | CUL4A |
| PHF8 | CCDC138 | ITGA1 | AMMECR1 | LAMP1 |
| SPIN4 | BCL2L11 | SNX18 | SYAP1 | AJUBA |
| MAGT1 | TMEM87B | MAP3K1 | DDX3X | IPO4 |
| ARMCX2 | STEAP3 | IPO11 | RP2 | HECTD1 |
| ATP11C | RALB | RASGRF2 | CLCN5 | EGLN3 |
| SLITRK4 | CCNT2 | VCAN | FAM199X | SPTSSA |
| SLC10A3 | PKP4 | PPIP5K2 | RNF128 | SNX6 |
| PRPS2 | CSRNP3 | C5orf30 | FMR1 | SEC23A |

| RP2 | GAD1 | TNFAIP8 | FBXO33 |
| --- | --- | --- | --- |
| CLCN5 | ITGA6 | SLC12A2 | VCPKMT |
| FAM199X | MAP3K20 | TGFBI | SAV1 |
| RNF128 | GULP1 | EGR1 | NIN |
| SLC25A43 | PMS1 | CTNNA1 | NID2 |
| VAMP7 | AOX1 | PAIP2 | TXNDC16 |
|  | FAM117B | PURA | GNPNAT1 |
|  | DGKD | CYSTM1 | DDHD1 |
|  | RNF24 | IK | ATG14 |
|  | TMEM230 | RNF14 | SIX4 |
|  | FERMT1 | CCNG1 | KCNH5 |
|  | NAPB | HMMR | PPP2R5E |
|  | SOGA1 | FGF18 | MAX |
|  | NFATC2 | CREBRF | PLEK2 |
|  | PLCB1 | FAF2 | RDH11 |
|  | ASXL1 | TXNDC5 | NUMB |
|  | PHF20 | BLOC1S5-TXNDC5 | TMED10 |
|  | TTPAL | HIST1H4H | ANGEL1 |
|  | NCOA3 | GABBR1 | SPTLC2 |
|  | ARFGEF2 | GTPBP2 | DIO2 |
|  | C21orf91 | PLA2G7 | FOXN3 |
|  | ADAMTS5 | TNFRSF21 | CCDC88C |
|  | MIS18A | ELOVL5 | TC2N |
|  | CLDN14 | COL12A1 | BTBD7 |
|  | PIGP | SESN1 | DICER1 |
|  | ERG | ECHDC1 | WARS |
|  | ZBTB21 | PTPRK | HSP90AA1 |
|  | SIK1 | BCLAF1 | BAG5 |
|  | SIK1B | KATNA1 | RNASE4 |
|  | RBM11 | FOXQ1 | AL163636.2 |
|  | HUNK | FOXF2 | BCL2L2 |
|  | SLC5A3 | SNRNP48 | NYNRIN |
|  | COL18A1 | ID4 | FOXG1 |
|  | ASCC2 | HIST1H2AE | G2E3 |
|  | PRR14L | PGBD1 | C14orf28 |
|  | RBFOX2 | MAPK14 | ARF6 |
|  | MYH9 | UBR2 | FRMD6 |
|  | FOXRED2 | PPP2R5D | GNG2 |
|  | SNU13 | SH3BGRL2 | SYNE2 |
|  | CERK | UFL1 | SUSD6 |
|  | NEFH | CEP57L1 | SMOC1 |
|  | TNRC6B | RFX6 | PCNX1 |
|  | FAM118A | NUS1 | DCAF4 |
|  | PPARA | RNF217 | PSEN1 |
|  | ATP2B2 | TBPL1 | RIOX1 |
|  | VGLL4 | MYB | FCF1 |
|  | RAF1 | TAB2 | JDP2 |
|  | XPC | PPP1R14C | TDP1 |
|  | METTL6 | MTHFD1L | OTUB2 |
|  | TOP2B | SCAF8 | GLRX5 |
|  | SLC4A7 | ACAT2 | BDKRB2 |
|  | CMTM6 | SLC22A3 | GSKIP |
|  | FYCO1 | MAP3K4 | VRK1 |
|  | CCR1 | MRM2 | RCOR1 |
|  | SETD2 | EIF2AK1 | CEP170B |
|  | PBRM1 | TWIST1 | NIPA1 |
|  | FOXP1 | IGF2BP3 | UBE3A |
|  | RYBP | OSBPL3 | TJP1 |

| SHQ1 | SKAP2 | BMF |
| --- | --- | --- |
| VGLL3 | HOXA11 | CCDC9B |
| SENP7 | HOXA13 | EHD4 |
| GCSAM | MPLKIP | UBR1 |
| TMEM39A | IGFBP3 | PPIP5K1 |
| FSTL1 | SLC25A40 | FBN1 |
| GOLGB1 | STEAP4 | SECISBP2L |
| OSBPL11 | PDK4 | TRPM7 |
| MBD4 | GIGYF1 | ONECUT1 |
| AMOTL2 | EPHB4 | RAB27A |
| PIK3CB | ORC5 | CCPG1 |
| ATR | POT1 | NEDD4 |
| HLTF | SLC35B4 | RFX7 |
| EIF5A2 | AKR1B1 | ZNF280D |
| BCL6 | C7orf49 | ADAM10 |
| PIGZ | MTPN | BNIP2 |
| CNTN4 | GLCCI1 | ANXA2 |
| SETD5 | PHF14 | ICE2 |
| PPARG | CCDC126 | PIF1 |
| SLC6A6 | EEPD1 | IGDCC3 |
| RARB | DBNL | INTS14 |
| RBMS3 | RABGEF1 | AAGAB |
| TGFBR2 | AC027644.4 | CLN6 |
| STT3B | DTX2 | MYO9A |
| PDCD6IP | PHTF2 | ADPGK |
| OXSR1 | GNAI1 | SIN3A |
| WDR48 | RUNDC3B | TSPAN3 |
| SNRK | GATAD1 | PEAK1 |
| LIMD1 | ARPC1A | SEC11A |
| SACM1L | ZKSCAN1 | POLG |
| KLHL18 | PRKAR2B | LYSMD4 |
| RPP14 | CBLL1 | CHSY1 |
| HTD2 | IFRD1 | THBS1 |
| PTPRG | CAV2 | PAK6 |
| ATXN7 | MET | BUB1B-PAK6 |
| PPP4R2 | KLHDC10 | KNL1 |
| NXPE3 | CPA4 | GCHFR |
| BBX | ACTR3B | CHP1 |
| PARP14 | EN2 | TYRO3 |
| NUDT16 | UBE3C | SNAP23 |
| CEP63 | CNOT7 | SEMA6D |
| SLC25A36 | REEP4 | USP8 |
| C3orf58 | TNFRSF10B | MINDY2 |
| TSC22D2 | NEFL | RNF111 |
| MBNL1 | PNMA2 | HACD3 |
| SMC4 | ZNF395 | PAQR5 |
| NLGN1 | FBXO16 | KIF23 |
| FXR1 | SLC20A2 | ARID3B |
| ATP11B | CA8 | C15orf39 |
| AP2M1 | PDE7A | ISL2 |
| LPP | MYBL1 | HMG20A |
| CCDC50 | TRAM1 | DNAJA4 |
| HES1 | UBE2W | IREB2 |
| PAK2 | ZFAND1 | MORF4L1 |
| SENP5 | SNX16 | TLNRD1 |
| FYTTD1 | CCNE2 | ZSCAN2 |
| AFAP1 | MTERF3 | PDE8A |
| BOD1L1 | STK3 | ABHD2 |

| SEL1L3 | RNF19A | IQGAP1 |
| --- | --- | --- |
| BEND4 | UBR5 | CRTC3 |
| FRYL | NUDCD1 | FURIN |
| CLOCK | HAS2 | CHD2 |
| ANKRD17 | AGO2 | SYNM |
| CXCL11 | SCRIB | MEF2A |
| CCNI | NRBP2 | TMEM8A |
| CNOT6L | TNKS | FBXL16 |
| EIF4E | SLC39A14 | UNKL |
| CENPE | CCAR2 | RNPS1 |
| TBCK | DPYSL2 | ADCY9 |
| ZGRF1 | ESCO2 | NAGPA |
| RNF150 | ERLIN2 | FOPNL |
| NEK1 | ADGRA2 | GDE1 |
| MFAP3L | BAG4 | DCUN1D3 |
| PIGG | PLEKHA2 | SEPHS2 |
| MRFAP1 | FNTA | VKORC1 |
| CPEB2 | AC110275.1 | AMFR |
| N4BP2 | STMN2 | CNOT1 |
| SLAIN2 | CTHRC1 | CMTM4 |
| REST | FAM91A1 | DYNC1LI2 |
| MOB1B | PHF20L1 | CHTF8 |
| ALB | DENND3 | PDF |
| EREG | AK3 | COG8 |
| THAP6 | CDKN2A | NOB1 |
| SHROOM3 | TOPORS | CMTR2 |
| PTPN13 | VCP | PHLPP2 |
| AFF1 | GBA2 | ZFHX3 |
| NFKB1 | C9orf64 | MBTPS1 |
| SGMS2 | AUH | TLDC1 |
| LARP7 | ZNF367 | COTL1 |
| ANK2 | KLF4 | ZDHHC7 |
| PCDH10 | PTBP3 | ZCCHC14 |
| ELMOD2 | INIP | CHMP1A |
| SMARCA5 | ALAD | NME4 |
| FAM160A1 | TNC | RAB40C |
| ARFIP1 | TTLL11 | JPT2 |
| TRIM2 | RNF208 | MLST8 |
| MND1 | SLC1A1 | TEDC2 |
| ANKH | LURAP1L | TNFRSF12A |
| PRLR | RECK | UBN1 |
| GDNF | OSTF1 | PMM2 |
| LIFR | CARD19 | MKL2 |
| RICTOR | PTPDC1 | ABCC1 |
| EMB | ERCC6L2 | ITPRIPL2 |
| MIER3 | ANP32B | VPS35L |
| DEPDC1B | COL15A1 | IQCK |
| ELOVL7 | TGFBR1 | TMEM159 |
| ENC1 | FKTN | METTL9 |
| FAM169A | SLC31A2 | EEF2K |
| ZBED3 | MRRF | UBFD1 |
| AP3B1 | SLC27A4 | DCTN5 |
| PCSK1 | SET | PRKCB |
| LOX | PMPCA | MAZ |
| FBN2 | AP1S2 | FBRS |
| P4HA2 | ARX | FBXL19 |
| IRF1 | ARMCX2 | CHD9 |
| AFF4 | MORF4L2 | MMP15 |

| SAR1B | AMMECR1 | C16orf70 |
| --- | --- | --- |
| DIAPH1 | CUL4B | NUTF2 |
| GEMIN5 | ENOX2 | PLA2G15 |
| EBF1 | SLITRK4 | HAS3 |
| PANK3 | SYAP1 | SNTB2 |
| FBXW11 | SAT1 | NIP7 |
| SH3PXD2B | ZFX | CYB5B |
| MGAT1 | RP2 | WWP2 |
| 44261 | NBDY | SF3B3 |
| TRIO | RNF128 | KIAA0513 |
| FAM105A | PAK3 | IRF8 |
| RAI14 | PLS3 | MAP1LC3B |
| DNAJC21 | SLC25A43 | DEF8 |
| ISL1 | HPRT1 | MYO1C |
| ITGA1 | FMR1 | PITPNA |
| ITGA2 | CSAG1 | SLC43A2 |
| GPX8 | EMD | TSR1 |
| MAP3K1 |  | NCBP3 |
| MAP1B |  | UBE2G1 |
| RASGRF2 |  | ZBTB4 |
| VCAN |  | ALOXE3 |
| RHOBTB3 |  | PIK3R5 |
| LNPEP |  | PMP22 |
| PPIP5K2 |  | SHMT1 |
| C5orf30 |  | FAM83G |
| FER |  | ULK2 |
| DCP2 |  | PIGS |
| TNFAIP8 |  | RAB34 |
| RAD50 |  | FAM222B |
| AC116366.3 |  | NUFIP2 |
| C5orf24 |  | SSH2 |
| KDM3B |  | BLMH |
| PURA |  | MYO19 |
| KIAA0141 |  | PCGF2 |
| RBM27 |  | PIP4K2B |
| AC091959.3 |  | ORMDL3 |
| SLC26A2 |  | TOP2A |
| LARP1 |  | TNS4 |
| CYFIP2 |  | SMARCE1 |
| CCNG1 |  | AC073508.2 |
| NPM1 |  | RETREG3 |
| FGF18 |  | EZH1 |
| CREBRF |  | VAT1 |
| CPEB4 |  | BRCA1 |
| FAF2 |  | MPP3 |
| NSD1 |  | ATXN7L3 |
| CNOT6 |  | GJC1 |
| TRERF1 |  | MAP3K14 |
| RCAN2 |  | PLEKHM1 |
| TNFRSF21 |  | CBX1 |
| DST |  | HOXB6 |
| COL12A1 |  | FAM117A |
| ELOVL4 |  | COL1A1 |
| EPHA7 |  | MBTD1 |
| PNISR |  | COX11 |
| SESN1 |  | TRIM25 |
| REV3L |  | APPBP2 |
| FYN |  | CYB561 |

| GOPC | SMARCD2 |
| --- | --- |
| ECHDC1 | HELZ |
| NHSL1 | GRB2 |
| SHPRH | UNC13D |
| SERAC1 | TRIM65 |
| SFT2D1 | TIMP2 |
| FOXF2 | AATK |
| PHACTR1 | NPLOC4 |
| FAM8A1 | ARHGDIA |
| ID4 | MAFG |
| ZSCAN16 | RAB40B |
| UHRF1BP1 | HIC1 |
| FOXP4 | PAFAH1B1 |
| SRF | CTNS |
| CD2AP | KIF1C |
| PHF3 | ACADVL |
| SENP6 | SLC2A4 |
| ANKRD6 | COX10 |
| LIN28B | HS3ST3B1 |
| PRDM1 | PIGL |
| CRYBG1 | GID4 |
| CEP57L1 | SMCR8 |
| MARCKS | ALDH3A2 |
| RWDD1 | MAP2K3 |
| DCBLD1 | TMEM97 |
| NUS1 | MRPL45 |
| TBPL1 | MLLT6 |
| ARFGEF3 | WIPF2 |
| ADGRG6 | IGFBP4 |
| PHACTR2 | CNP |
| UTRN | MLX |
| TAB2 | CNTNAP1 |
| MTHFD1L | G6PC3 |
| AKAP12 | CALCOCO2 |
| TULP4 | UBE2Z |
| MAP3K4 | IGF2BP1 |
| AFDN | KAT7 |
| DGKB | AKAP1 |
| ANKMY2 | YPEL2 |
| TWIST1 | TANC2 |
| IGF2BP3 | DCAF7 |
| OSBPL3 | MAP3K3 |
| SKAP2 | DDX42 |
| HOXA11 | PRKCA |
| HIBADH | PITPNC1 |
| INHBA | PRKAR1A |
| PURB | KCNJ2 |
| IGFBP3 | KCTD2 |
| TNS3 | TSEN54 |
| HIP1 | ITGB4 |
| PCLO | UBALD2 |
| PDK4 | MGAT5B |
| ASNS | SEC14L1 |
| ZNF394 | SEPT9 |
| EPHB4 | SYNGR2 |
| PUS7 | CBX2 |
| SLC35B4 | CCDC137 |
| C7orf49 | SLC25A10 |

| KIAA1549 | AC139530.2 |
| --- | --- |
| HIPK2 | COLEC12 |
| KMT2C | ENOSF1 |
| PHF14 | GAREM1 |
| CCDC126 | TPGS2 |
| TAX1BP1 | SMAD2 |
| CREB5 | MEX3C |
| BMPER | BCL2 |
| CDK13 | TMX3 |
| MRPL32 | ZNF516 |
| DBNL | TXNL4A |
| RABGEF1 | USP14 |
| AC027644.4 | SMCHD1 |
| TMEM248 | TWSG1 |
| PHTF2 | RAB31 |
| RUNDC3B | PRELID3A |
| ZNF655 | MIB1 |
| ZKSCAN1 | TAF4B |
| TES | RNF138 |
| CAV2 | GALNT1 |
| CALU | C18orf25 |
| CPA4 | SKA1 |
| MKLN1 | MAPK4 |
| INSIG1 | ME2 |
| EN2 | KIAA1468 |
| UBE3C | SERPINB8 |
| FAM167A | CTDP1 |
| CNOT7 | BTBD2 |
| PSD3 | MKNK2 |
| ZNF395 | AP3D1 |
| FBXO16 | TIMM13 |
| KIF13B | LMNB2 |
| SLC20A2 | GPR108 |
| PLAG1 | S1PR2 |
| CA8 | CDC37 |
| ASPH | TMED1 |
| MYBL1 | KANK2 |
| NCOA2 | PRKACA |
| TRAM1 | ASF1B |
| JPH1 | DNAJB1 |
| CCNE2 | BRD4 |
| RNF19A | WIZ |
| UBR5 | ELL |
| TRPS1 | ZNF43 |
| SAMD12 | RHPN2 |
| HAS2 | ZNF565 |
| SCRIB | DYRK1B |
| NRBP2 | NUMBL |
| TNKS | POU2F2 |
| SLC39A14 | TMEM143 |
| CCAR2 | BCAT2 |
| CHMP7 | NUP62 |
| DOCK5 | C19orf48 |
| ERLIN2 | ZNF577 |
| ASH2L | ZNF615 |
| BAG4 | PPP6R1 |
| DDHD2 | ZNF772 |
| PLEKHA2 | PTBP1 |

| VDAC3 | CSNK1G2 |
| --- | --- |
| FNTA | NFIC |
| AC110275.1 | FZR1 |
| TGS1 | UHRF1 |
| ZFHX4 | ZNF557 |
| DECR1 | C19orf66 |
| ESRP1 | ILF3 |
| INTS8 | CARM1 |
| SDC2 | LDLR |
| VPS13B | SWSAP1 |
| ZHX2 | NACC1 |
| PHF20L1 | MRI1 |
| DENND3 | ZSWIM4 |
| PTPRD | CC2D1A |
| PSIP1 | ADGRE5 |
| BNC2 | RAB8A |
| MLLT3 | GTPBP3 |
| TLN1 | ZNF254 |
| RNF38 | ZNF507 |
| PAX5 | KCTD15 |
| PTAR1 | LSM14A |
| GNAQ | UBA2 |
| ZNF367 | ZFP36 |
| ABCA1 | PLD3 |
| ELP1 | SHKBP1 |
| TMEM245 | AXL |
| PTPN3 | ZNF230 |
| PTBP3 | PVR |
| ALAD | NECTIN2 |
| TNC | TOMM40 |
| FBXW2 | QPCTL |
| PPP6C | ARHGAP35 |
| CAMSAP1 | BICRA |
| SLC1A1 | GRWD1 |
| LURAP1L | MYADM |
| ACER2 | ZNF580 |
| MTAP | ZNF444 |
| TLE4 | ZNF71 |
| SPIN1 | ZNF304 |
| CARD19 | ZNF274 |
| ANP32B | PXDN |
| COL15A1 | SDC1 |
| SLC44A1 | LDAH |
| FKTN | PTRHD1 |
| DNAJC25 | DNAJC27 |
| COL27A1 | SLC5A6 |
| TRIM32 | SLC30A3 |
| MRRF | PPM1G |
| ZBTB34 | STRN |
| SLC27A4 | PRKD3 |
| COL5A1 | ZFP36L2 |
| ARX | PREPL |
| PHF8 | RTN4 |
| ATRX | EFEMP1 |
| MAGT1 | BCL11A |
| ARMCX2 | SERTAD2 |
| RAB9B | C1D |
| AMMECR1 | DUSP11 |

| UPF3B | DCTN1 |
| --- | --- |
| THOC2 | TGOLN2 |
| MBNL3 | KRCC1 |
| SLITRK4 | STARD7 |
| SLC10A3 | TMEM127 |
| SHROOM2 | ANKRD39 |
| PRPS2 | SEPT10 |
| SYAP1 | SLC35F5 |
| NHS | BIN1 |
| TSPAN7 | SMPD4 |
| AF241726.2 | FAM168B |
| RP2 | CXCR4 |
| CLCN5 | RND3 |
| ATP7A | ARL5A |
| PGK1 | AC068547.1 |
| FAM199X | STAM2 |
| RNF128 | BAZ2B |
| TMEM164 | DPP4 |
| SLC25A43 | FIGN |
| FMR1 | GALNT3 |
| VAMP7 | ATP5MC3 |
|  | LNPK |
|  | TTC30A |
|  | HECW2 |
|  | PGAP1 |
|  | TRAK2 |
|  | RAPH1 |
|  | METTL21A |
|  | FZD5 |
|  | IKZF2 |
|  | FN1 |
|  | MREG |
|  | TNS1 |
|  | AP1S3 |
|  | SERPINE2 |
|  | SLC16A14 |
|  | ARL4C |
|  | HDAC4 |
|  | HDLBP |
|  | PQLC3 |
|  | LPIN1 |
|  | VSNL1 |
|  | SELENOI |
|  | KHK |
|  | SNX17 |
|  | ZNF512 |
|  | YPEL5 |
|  | LCLAT1 |
|  | CRIM1 |
|  | EML4 |
|  | PRKCE |
|  | EPAS1 |
|  | RHOQ |
|  | SOCS5 |
|  | FOXN2 |
|  | LGALSL |
|  | AFTPH |
|  | ANTXR1 |

| MXD1 |
| --- |
| SEMA4F |
| TCF7L1 |
| MAT2A |
| RPIA |
| SLC9A2 |
| CCDC138 |
| BCL2L11 |
| MERTK |
| DDX18 |
| STEAP3 |
| RALB |
| R3HDM1 |
| KYNU |
| LYPD6 |
| FMNL2 |
| GPD2 |
| CERS6 |
| BBS5 |
| GAD1 |
| ITGA6 |
| MAP3K20 |
| HNRNPA3 |
| ITGAV |
| MFSD6 |
| NAB1 |
| FAM117B |
| NRP2 |
| CCNYL1 |
| TMEM169 |
| KCNE4 |
| AGFG1 |
| ATG16L1 |
| SH3BP4 |
| TBC1D20 |
| NSFL1C |
| CENPB |
| RNF24 |
| TMEM230 |
| PCNA |
| TMX4 |
| RRBP1 |
| NAPB |
| PLAGL2 |
| RBM12 |
| NFS1 |
| B4GALT5 |
| SPATA2 |
| ATP9A |
| ARFRP1 |
| ZNF512B |
| ZCCHC3 |
| TRIB3 |
| SIRPA |
| ATRN |
| PRNP |
| MCM8 |
| BMP2 |

| BTBD3 |
| --- |
| XRN2 |
| GINS1 |
| HM13 |
| POFUT1 |
| CHMP4B |
| CEP250 |
| AAR2 |
| C20orf24 |
| TGIF2-C20orf24 |
| RPRD1B |
| RALGAPB |
| SRSF6 |
| HNF4A |
| TTPAL |
| STK4 |
| PIGT |
| DNTTIP1 |
| ZSWIM3 |
| ARFGEF2 |
| PTPN1 |
| PARD6B |
| RBM38 |
| RAB22A |
| STX16 |
| GNAS |
| FAM217B |
| SLC17A9 |
| TPD52L2 |
| PCMTD2 |
| HSPA13 |
| C21orf91 |
| CYYR1 |
| ADAMTS5 |
| LTN1 |
| MIS18A |
| C21orf59 |
| RUNX1 |
| CLDN14 |
| VPS26C |
| ERG |
| BRWD1 |
| C2CD2 |
| ZBTB21 |
| SIK1 |
| SIK1B |
| USP25 |
| GABPA |
| SLC5A3 |
| CBR1 |
| MORC3 |
| DYRK1A |
| BACE2 |
| RRP1B |
| PDXK |
| TRAPPC10 |
| FP565260.7 |
| ADARB1 |

| COL18A1 |
| --- |
| ZNF280B |
| ZNF70 |
| PITPNB |
| XBP1 |
| THOC5 |
| CASTOR1 |
| AC004997.1 |
| SF3A1 |
| RBFOX2 |
| MYH9 |
| FOXRED2 |
| TMEM184B |
| CSNK1E |
| DDX17 |
| JOSD1 |
| CBX6 |
| RANGAP1 |
| NAGA |
| POLDIP3 |
| SHISAL1 |
| CELSR1 |
| CERK |
| BCR |
| GRK3 |
| FBXO7 |
| CDC42EP1 |
| PDXP |
| Z83844.3 |
| MICALL1 |
| GTPBP1 |
| SYNGR1 |
| NUP50 |
| FAM118A |
| PPARA |
| TRMU |
| PIM3 |
| SUMF1 |
| RAD18 |
| ATP2B2 |
| VGLL4 |
| MRPS25 |
| ANKRD28 |
| SATB1 |
| SLC4A7 |
| CMTM6 |
| GLB1 |
| SUSD5 |
| LRRFIP2 |
| CSRNP1 |
| CDCP1 |
| FYCO1 |
| CCR1 |
| SMARCC1 |
| PRKAR2A |
| WDR82 |
| PBRM1 |
| DCP1A |

| DENND6A |
| --- |
| MAGI1 |
| EOGT |
| FRMD4B |
| FOXP1 |
| RYBP |
| SHQ1 |
| VGLL3 |
| DCBLD2 |
| TMEM39A |
| LRRC58 |
| FSTL1 |
| KPNA1 |
| OSBPL11 |
| ISY1 |
| CNBP |
| TMCC1 |
| AMOTL2 |
| DZIP1L |
| XRN1 |
| PLOD2 |
| PLSCR1 |
| HLTF |
| TM4SF1 |
| C3orf33 |
| AC104472.3 |
| SLC33A1 |
| SSR3 |
| LXN |
| PHC3 |
| EIF5A2 |
| PLD1 |
| NCEH1 |
| TBL1XR1 |
| GNB4 |
| ABCC5 |
| TRA2B |
| BCL6 |
| TFRC |
| UBXN7 |
| MELTF |
| EDEM1 |
| MTMR14 |
| JAGN1 |
| TATDN2 |
| AC022384.1 |
| SYN2 |
| MKRN2 |
| SLC6A6 |
| NR2C2 |
| EAF1 |
| RAB5A |
| NR1D2 |
| RARB |
| RBMS3 |
| TGFBR2 |
| PDCD6IP |
| OXSR1 |

| ACVR2B |
| --- |
| CTNNB1 |
| NKTR |
| ZBTB47 |
| SNRK |
| TCAIM |
| LIMD1 |
| KLHL18 |
| ARIH2 |
| WDR6 |
| DAG1 |
| GNAI2 |
| MAPKAPK3 |
| RAD54L2 |
| RPP14 |
| HTD2 |
| MITF |
| PCNP |
| NXPE3 |
| ALCAM |
| SLC35A5 |
| ATP6V1A |
| ADPRH |
| PLXNA1 |
| RAB7A |
| ZBTB38 |
| RNF7 |
| U2SURP |
| HPS3 |
| TSC22D2 |
| P2RY1 |
| IL12A |
| PRKCI |
| FNDC3B |
| MFN1 |
| NDUFB5 |
| FXR1 |
| ATP11B |
| DVL3 |
| AP2M1 |
| LPP |
| IL1RAP |
| HES1 |
| FYTTD1 |
| TMEM129 |
| MRFAP1L1 |
| AFAP1 |
| TAPT1 |
| APBB2 |
| FRYL |
| USP46 |
| PPAT |
| HOPX |
| GRSF1 |
| CXCL11 |
| SCARB2 |
| CNOT6L |
| SEC31A |

| LIN54 |
| --- |
| SLC39A8 |
| CENPE |
| LEF1 |
| ELOVL6 |
| ANKRD50 |
| SLC7A11 |
| SETD7 |
| RNF150 |
| OTUD4 |
| LRBA |
| SH3D19 |
| C4orf46 |
| SH3RF1 |
| NEK1 |
| MFAP3L |
| FGFR3 |
| NSD2 |
| HTT |
| EVC |
| MRFAP1 |
| CPEB2 |
| PACRGL |
| PCDH7 |
| RHOH |
| SLAIN2 |
| KIT |
| SRD5A3 |
| EREG |
| THAP6 |
| USO1 |
| COPS4 |
| GPAT3 |
| NPNT |
| SGMS2 |
| USP53 |
| FGF2 |
| JADE1 |
| PCDH10 |
| ELMOD2 |
| ABCE1 |
| EDNRA |
| TRIM2 |
| TMEM144 |
| RAPGEF2 |
| FAM218A |
| SNX25 |
| ANKH |
| MYO10 |
| GOLPH3 |
| RAD1 |
| LIFR |
| IL6ST |
| MIER3 |
| PLK2 |
| PDE4D |
| ELOVL7 |
| SREK1IP1 |

| ENC1 |
| --- |
| F2RL2 |
| ZBED3 |
| AP3B1 |
| LHFPL2 |
| ARSB |
| MTX3 |
| MBLAC2 |
| LYSMD3 |
| TTC37 |
| CHD1 |
| PJA2 |
| STARD4 |
| EPB41L4A |
| MCC |
| FEM1C |
| ALDH7A1 |
| FBN2 |
| FNIP1 |
| P4HA2 |
| IRF1 |
| SEPT8 |
| AFF4 |
| PPP2CA |
| CDC23 |
| DNAJC18 |
| HARS |
| DIAPH1 |
| NR3C1 |
| PDGFRB |
| DCTN4 |
| GEMIN5 |
| FBXW11 |
| BOD1 |
| RNF44 |
| DBN1 |
| TBC1D9B |
| MGAT1 |
| PAPD7 |
| 44261 |
| FAM105A |
| OTULIN |
| C5orf22 |
| RAI14 |
| ZNF131 |
| ISL1 |
| ITGA2 |
| GPX8 |
| MAP3K1 |
| IPO11 |
| ZFYVE16 |
| RASA1 |
| POLR3G |
| RGMB |
| PPIP5K2 |
| C5orf30 |
| WDR36 |
| DCP2 |

| TNFAIP8 |
| --- |
| PRRC1 |
| ISOC1 |
| CDC42SE2 |
| SLC22A5 |
| TGFBI |
| FAM53C |
| EGR1 |
| CTNNA1 |
| PAIP2 |
| PURA |
| CYSTM1 |
| IK |
| KIAA0141 |
| RNF14 |
| NDFIP1 |
| TCOF1 |
| SLC36A1 |
| G3BP1 |
| MFAP3 |
| CCNG1 |
| HMMR |
| NPM1 |
| FGF18 |
| ERGIC1 |
| CREBRF |
| CPEB4 |
| FAF2 |
| GRK6 |
| CNOT6 |
| SERPINB9 |
| SLC22A23 |
| SSR1 |
| TXNDC5 |
| BLOC1S5-TXNDC5 |
| ATXN1 |
| HIST1H2BC |
| HIST1H4H |
| LEMD2 |
| C6orf106 |
| SRPK1 |
| GTPBP2 |
| TNFRSF21 |
| TRAM2 |
| ELOVL5 |
| COL12A1 |
| EPHA7 |
| MMS22L |
| BVES |
| CDK19 |
| GOPC |
| ECHDC1 |
| PTPRK |
| SGK1 |
| BCLAF1 |
| SF3B5 |
| NUP43 |
| CNKSR3 |

| IRF4 |
| --- |
| FOXF2 |
| FOXC1 |
| RIPK1 |
| CD83 |
| FAM8A1 |
| ID4 |
| HIST1H2AC |
| HIST1H1E |
| HIST1H2BD |
| HIST1H2BE |
| HIST1H2AE |
| HIST1H3E |
| BTN2A1 |
| HIST1H2AI |
| HIST1H3H |
| HIST1H2BN |
| PGBD1 |
| UHRF1BP1 |
| PPARD |
| KCTD20 |
| C6orf89 |
| CNPY3 |
| PPP2R5D |
| SRF |
| BAG2 |
| CD109 |
| SH3BGRL2 |
| NT5E |
| PM20D2 |
| ANKRD6 |
| PRDM1 |
| FOXO3 |
| AMD1 |
| MFSD4B |
| MARCKS |
| NUS1 |
| L3MBTL3 |
| TBPL1 |
| TNFAIP3 |
| ADGRG6 |
| AIG1 |
| PHACTR2 |
| SASH1 |
| MTHFD1L |
| SCAF8 |
| ARID1B |
| ACAT2 |
| SLC22A3 |
| QKI |
| MRM2 |
| ACTB |
| CYTH3 |
| KDELR2 |
| ZNF12 |
| TWIST1 |
| TWISTNB |
| SP8 |

| IGF2BP3 |
| --- |
| OSBPL3 |
| SKAP2 |
| HOXA1 |
| HOXA11 |
| HOXA13 |
| KIAA0895 |
| MPLKIP |
| H2AFV |
| PURB |
| IGFBP3 |
| TNS3 |
| POM121C |
| HIP1 |
| SEMA3C |
| SLC25A40 |
| CDK6 |
| BET1 |
| PDK4 |
| SLC25A13 |
| SMURF1 |
| GIGYF1 |
| EPHB4 |
| ORC5 |
| SYPL1 |
| NAMPT |
| CCDC71L |
| BMT2 |
| POT1 |
| GCC1 |
| TMEM209 |
| PODXL |
| SLC35B4 |
| C7orf49 |
| MTPN |
| CREB3L2 |
| KIAA1549 |
| ZC3HAV1 |
| LMBR1 |
| DNAAF5 |
| GPER1 |
| MAFK |
| LFNG |
| FOXK1 |
| WIPI2 |
| GLCCI1 |
| CCDC126 |
| CREB5 |
| ZNRF2 |
| BMPER |
| EEPD1 |
| ANLN |
| DBNL |
| YKT6 |
| RABGEF1 |
| AC027644.4 |
| TMEM248 |
| DTX2 |

| PHTF2 |
| --- |
| GNAI1 |
| CROT |
| PEG10 |
| NPTX2 |
| ARPC1A |
| ARPC1B |
| ZKSCAN1 |
| AGFG2 |
| PRKAR2B |
| IFRD1 |
| TES |
| CAV2 |
| WNT16 |
| HILPDA |
| NRF1 |
| KLHDC10 |
| CPA4 |
| CALD1 |
| UBN2 |
| AGK |
| ZNF282 |
| REPIN1 |
| ACTR3B |
| INSIG1 |
| EN2 |
| NOM1 |
| UBE3C |
| DNAJB6 |
| FAM167A |
| CNOT7 |
| MTUS1 |
| ASAH1 |
| PSD3 |
| HR |
| REEP4 |
| TNFRSF10B |
| NEFL |
| PNMA2 |
| TRIM35 |
| ZNF395 |
| FBXO16 |
| KIF13B |
| FGFR1 |
| SLC20A2 |
| SNAI2 |
| OPRK1 |
| PLAG1 |
| IMPAD1 |
| CA8 |
| MYBL1 |
| TRAM1 |
| PAG1 |
| ZFAND1 |
| SNX16 |
| TP53INP1 |
| STK3 |
| RNF19A |

| YWHAZ |
| --- |
| RRM2B |
| UBR5 |
| KLF10 |
| SLC25A32 |
| NUDCD1 |
| SYBU |
| TRPS1 |
| SNTB1 |
| HAS2 |
| ZHX1 |
| ASAP1 |
| SLA |
| NDRG1 |
| AGO2 |
| SCRIB |
| NRBP2 |
| ARHGEF10 |
| KBTBD11-OT1 |
| MTMR9 |
| SLC7A2 |
| SLC39A14 |
| CCAR2 |
| BNIP3L |
| DPYSL2 |
| EXTL3 |
| RBPMS |
| ERLIN2 |
| ADGRA2 |
| ASH2L |
| BAG4 |
| TACC1 |
| PLEKHA2 |
| TCIM |
| HGSNAT |
| SPIDR |
| GDAP1 |
| PI15 |
| OSGIN2 |
| ESRP1 |
| LAPTM4B |
| CTHRC1 |
| EBAG9 |
| FAM91A1 |
| PHF20L1 |
| DENND3 |
| MAF1 |
| GLIS3 |
| AK3 |
| BNC2 |
| KLHL9 |
| B4GALT1 |
| TLN1 |
| RNF38 |
| FBXO10 |
| SHB |
| PTAR1 |
| UBQLN1 |

| C9orf64 |
| --- |
| AUH |
| IPPK |
| NINJ1 |
| SLC35D2 |
| TSTD2 |
| TRIM14 |
| ABCA1 |
| KLF4 |
| ELP1 |
| TMEM245 |
| EPB41L4B |
| PTBP3 |
| INIP |
| ALAD |
| POLE3 |
| TNC |
| CDK5RAP2 |
| MEGF9 |
| FBXW2 |
| PSMD5 |
| TRAF1 |
| STOM |
| ZBTB6 |
| DENND1A |
| GOLGA1 |
| DPM2 |
| SPOUT1 |
| TSC1 |
| MED22 |
| SURF4 |
| SEC16A |
| ABCA2 |
| PNPLA7 |
| SLC1A1 |
| LURAP1L |
| ACER2 |
| MTAP |
| UBE2R2 |
| TRMT10B |
| DCAF10 |
| FXN |
| GDA |
| SPIN1 |
| CARD19 |
| PTPDC1 |
| ANP32B |
| COL15A1 |
| TGFBR1 |
| ZNF189 |
| FAM206A |
| SLC31A1 |
| PRPF4 |
| TMEM268 |
| PAPPA |
| NEK6 |
| ZBTB34 |
| DNM1 |

| SLC27A4 |
| --- |
| URM1 |
| PTPA |
| PRRC2B |
| AP1S2 |
| SCML2 |
| SH3KBP1 |
| MAP7D2 |
| RPS6KA3 |
| TAB3 |
| SLC35A2 |
| IQSEC2 |
| SMC1A |
| PHF8 |
| SPIN4 |
| MAGT1 |
| ARMCX2 |
| MORF4L2 |
| PSMD10 |
| AMMECR1 |
| SEPT6 |
| LAMP2 |
| CUL4B |
| HS6ST2 |
| ATP11C |
| SLITRK4 |
| TREX2 |
| HAUS7 |
| MECP2 |
| SLC10A3 |
| PRPS2 |
| SYAP1 |
| SAT1 |
| MAGEB2 |
| DDX3X |
| KRBOX4 |
| RP2 |
| CLCN5 |
| MSN |
| OGT |
| CHIC1 |
| RNF128 |
| NXT2 |
| PGRMC1 |
| SLC25A43 |
| BCORL1 |
| PHF6 |
| SLC9A6 |
| FMR1 |
| EMD |
