# Supplementary material for: Long non-coding RNA linc00921 suppresses tumorigenesis and epithelial-to-mesenchymal transition of triple-negative breast cancer via targeting miR-9-5p/LZTS2 axis
Source: Hum Cell. 2022 Feb 18;35(3):909–23. doi: 10.1007/s13577-022-00685-6 (PMC9013323; doi:10.1007/s13577-022-00685-6)
Supplement: Supplementary file 8 — Supplementary file8 (DOCX 17 KB) [file 13577_2022_685_MOESM8_ESM.docx]

**Supplementary Table 6 Overlapped genes of bioinformatics algorithms**

| UBE2Q1 |
| --- |
| SHC1 |
| SCYL3 |
| LDLRAP1 |
| ARID1A |
| SNX7 |
| MAN1A2 |
| FAM46C |
| PRUNE1 |
| ATP8B2 |
| SLC50A1 |
| ATP1B1 |
| FAM107B |
| NRP1 |
| PARG |
| FAM13C |
| CCDC6 |
| CPEB3 |
| BICC1 |
| SIRT1 |
| LZTS2 |
| SFXN2 |
| M6PR |
| YBX3 |
| SENP1 |
| ARL1 |
| NCOR2 |
| ESYT1 |
| SACS |
| FBXL3 |
| FREM2 |
| KLF5 |
| CUL4A |
| IPO4 |
| NID2 |
| GNPNAT1 |
| TRPM7 |
| ONECUT1 |
| NEDD4 |
| TLNRD1 |
| MKL2 |
| SMARCD2 |
| MAP3K3 |
| KCNJ2 |
| COLEC12 |
| TAF4B |
| RAB8A |
| LSM14A |
| SOCS5 |
| RBFOX2 |
| FOXP1 |
| BCL6 |
| RBMS3 |
| PDCD6IP |
| NXPE3 |
| RNF150 |
| CPEB2 |
| PCDH10 |
| TRIM2 |
| AP3B1 |
| FBN2 |
| P4HA2 |
| CCNG1 |
| FAF2 |
| ID4 |
| HOXA11 |
| IGFBP3 |
| PDK4 |
| DBNL |
| UBE3C |
| SCRIB |
| CCAR2 |
| TNC |
| LURAP1L |
| COL15A1 |
| ARMCX2 |
| RNF128 |
