# Supplementary material for: Long non-coding RNA linc00921 suppresses tumorigenesis and epithelial-to-mesenchymal transition of triple-negative breast cancer via targeting miR-9-5p/LZTS2 axis
Source: Hum Cell. 2022 Feb 18;35(3):909–23. doi: 10.1007/s13577-022-00685-6 (PMC9013323; doi:10.1007/s13577-022-00685-6)
Supplement: Supplementary file 9 — Supplementary file9 (DOCX 60 KB) [file 13577_2022_685_MOESM9_ESM.docx]

| **Downregulated genes** | **Upregulated genes** |
| --- | --- |
| APOH | CGA |
| PRB2 | COL11A1 |
| SULT2A1 | MYB |
| FGB | ATP6V0A4 |
| HBG1 | MMP13 |
| ALB | GRP |
| FGA | AP1M2 |
| AADAC | PRSS22 |
| HBB | MMP1 |
| SFRP5 | CST1 |
| HBA2 | RAB25 |
| ITIH2 | EDN2 |
| FABP1 | BSPRY |
| GLYAT | IL20 |
| AKR1C2 | ASCL1 |
| TNMD | E2F7 |
| ALDH1L1 | ELF3 |
| MAOA | LAD1 |
| NRXN1 | CST4 |
| HSD17B13 | LYPD3 |
| ACSM2A | ASPM |
| CPB2 | BUB1B |
| ANGPTL5 | DEPDC1 |
| TIMP4 | KRT6B |
| THRSP | TJP3 |
| TSPAN8 | MELK |
| UGT2B4 | OLR1 |
| PLG | E2F8 |
| FGL1 | TMEM270 |
| CIDEA | KIF14 |
| CES1 | B4GALNT4 |
| OR6A2 | PBK |
| C8B | ASPHD1 |
| CDO1 | BUB1 |
| MYOT | ESRP1 |
| HEG1 | CRABP2 |
| PLIN1 | NUF2 |
| ADIPOQ | ITGB6 |
| C2orf40 | KRT8 |
| HBD | PLA2G4F |
| CKMT2 | ESRP2 |
| MYOM1 | CDC20 |
| CYP3A4 | CCNB2 |
| SLC10A1 | EPCAM |
| TF | PDXP |
| SCARA5 | FSCN3 |
| ATXN3L | TFAP2A |
| NTRK2 | MUC1 |
| FAM13A | IGHV3OR16-9 |
| CYP3A5 | FAM83B |

**Supplementary Table 7 Downregulated and upregulated mRNAs in TNBC of GSE115275**

| LIPE | CST2 |
| --- | --- |
| S100B | PTK7 |
| GPAM | KIF20A |
| CAVIN2 | KRT2 |
| CIDEC | CENPF |
| RERGL | PET100 |
| G0S2 | RBBP8NL |
| XAGE3 | CEP55 |
| LEP | MMP3 |
| TRHDE | NEK2 |
| KNG1 | ATP2C2 |
| GYG2 | S100A14 |
| TMTC1 | EXO1 |
| TRIM6-TRIM34 | CDCA3 |
| ZBTB16 | RRM2 |
| C1QTNF9B | CDC45 |
| ADH1B | ESCO2 |
| ABCD2 | TTK |
| GP5 | TOP2A |
| IL6 | PKIB |
| PPP1R14A | HMMR |
| FGFBP2 | EPYC |
| ADRB1 | PPEF1 |
| GYS2 | PAFAH1B3 |
| LHCGR | CDKN2A |
| RXRG | FAM83D |
| HSPB6 | ERBB3 |
| CFD | ERCC6L |
| ACVR1C | GTSE1 |
| SCN4A | CDC25C |
| NLGN1 | HIST1H2AI |
| MAOB | KRT80 |
| RDH12 | ADAMDEC1 |
| OR52E8 | ANLN |
| SVEP1 | CELSR1 |
| AUTS2 | CXCL11 |
| EPB42 | SOX11 |
| ABCA8 | HOOK1 |
| CT47A12 | ERBB2 |
| RBP4 | PITX1 |
| HPSE2 | OR2B6 |
| C1QTNF9 | KRT19 |
| ADGRB3 | ATRNL1 |
| SLC51B | SOWAHB |
| SORBS1 | COL10A1 |
| NPY5R | HIST1H3G |
| DMGDH | HIST1H2BG |
| FHL5 | CDS1 |
| MATN2 | HIST1H2BF |
| DNASE1L3 | NECTIN4 |
| LPL | DEPDC1B |

| CFHR2 | SMKR1 |
| --- | --- |
| RGN | NMU |
| BMP5 | SMARCB1 |
| SLC16A12 | TTC39A |
| CYP3A7 | SFN |
| EYS | PTK6 |
| HRC | OIP5 |
| AOX1 | HOMER2 |
| MRAP | CENPM |
| KIF25 | POLQ |
| BCHE | MKI67 |
| KCNB1 | LLGL2 |
| PPP1R1A | PLPP2 |
| GABRG1 | IBSP |
| MAP1LC3C | TMEM132A |
| MYH11 | CDC42BPG |
| SLC7A10 | CXADR |
| FREM1 | ZNF750 |
| GPC3 | SPINT2 |
| FABP9 | CDH1 |
| SLC22A3 | EPHA10 |
| AMBP | MB |
| ANGPTL1 | KIF4A |
| VIT | FBN2 |
| ALX3 | MYBL2 |
| ZNF502 | SCNN1A |
| PAGE4 | DLGAP5 |
| TCF15 | KIF11 |
| EBF1 | SAPCD2 |
| PALMD | SHROOM3 |
| CFHR3 | STYK1 |
| ITIH5 | SLC25A35 |
| ABCC6 | TK1 |
| CAV1 | CYP2B6 |
| NAALAD2 | FCGR1A |
| MOGAT1 | MTFR2 |
| DNAJB13 | SHCBP1 |
| MT1A | SH2D3A |
| IGFBP6 | UNC5B |
| DPRX | KIF4B |
| PLIN5 | ARL10 |
| APOD | CDCA5 |
| BTN1A1 | CDK1 |
| GHR | CA12 |
| ITIH3 | IRF6 |
| AQP7 | CORIN |
| DMRT2 | PRC1 |
| GDF10 | SSX2IP |
| AKAP12 | DPPA5 |
| LRP1B | TESMIN |
| PDC | CKAP2L |

| MMP27 | NDC80 |
| --- | --- |
| HP | DDR1 |
| CPED1 | KNL1 |
| AGMO | KIFC1 |
| STXBP6 | GRB7 |
| TNNT3 | RECQL4 |
| KCNA5 | MPZL3 |
| GC | C1orf210 |
| F10 | KIF23 |
| AKR1C8P | CCL11 |
| SMAD9 | CDC25A |
| PMP2 | RAD51 |
| APOA1 | F12 |
| OR10C1 | KRT85 |
| ANKRD29 | UBE2C |
| LRRIQ3 | UHRF1 |
| CHRDL1 | CDCA4 |
| SLC2A4 | SLC2A1 |
| LZTS2 | C11orf80 |
| GABRA2 | MAB21L4 |
| NTRK3 | TPX2 |
| COL25A1 | SYCE2 |
| MUSTN1 | CDKN3 |
| AFM | KIF18A |
| EVC | STXBP2 |
| PLAC9 | FBXO43 |
| CYP4F12 | INTS8 |
| PPBP | CLDN4 |
| CHL1 | S100A2 |
| CCNYL1 | NCAPG |
| SAA4 | HIST2H3C |
| FMO2 | KIF2C |
| ADH1A | CRYBB3 |
| WASF3 | SALL4 |
| CASQ2 | ZWINT |
| LRRC4B | ANKRD22 |
| HCAR2 | GPR50 |
| FAM162B | DTL |
| RNF207 | CGN |
| FGF2 | WDR76 |
| CDY2B | SMC1B |
| ASPA | GPR19 |
| CFHR4 | BIK |
| F2 | SKA1 |
| CYP8B1 | ST14 |
| AVPR1A | MYO5B |
| EBF3 | KRT83 |
| MBL2 | RASGRP1 |
| CSN1S1 | CLECL1 |
| LMO3 | FCGR3A |
| SLIT3 | STIL |

| ALAS2 | PLEK2 |
| --- | --- |
| MEOX1 | TFR2 |
| NTS | SPP1 |
| COL4A1 | AUNIP |
| MYH1 | ESPL1 |
| KCNAB1 | TMPRSS13 |
| LVRN | TRIP13 |
| IL33 | TACSTD2 |
| MED12L | CLPS |
| ADH4 | CENPE |
| RDH5 | IL17RB |
| CTNNA3 | NIPA2 |
| HHIP | KIAA1210 |
| ADRA2A | TPD52 |
| CADM2 | STARD10 |
| SLC14A1 | TRPS1 |
| MEOX2 | TREM1 |
| PENK | AURKA |
| ANK2 | ADORA3 |
| HSPA12A | TRAF4 |
| MYOM2 | HIST1H3H |
| CAV3 | TYMP |
| PTPRQ | SLC7A11 |
| HPD | MST1R |
| COX7A1 | CENPU |
| FBXO40 | FCGR1B |
| WNT11 | RHPN2 |
| FCN2 | ST8SIA6 |
| SCN9A | GIPC1 |
| HAO1 | TMEM244 |
| CD36 | ROR2 |
| FAM107A | MARVELD3 |
| ADIRF | VAV3 |
| LRRC2 | GGACT |
| VEGFD | ERMN |
| ABCA9 | IGSF3 |
| KLF14 | RHPN1 |
| NR0B1 | ATP6V0B |
| LPA | CABLES2 |
| ABCA6 | AP1S3 |
| ADAMTS5 | GALNT5 |
| HSD11B1 | FAM72D |
| SNCG | HTRA4 |
| TEX50 | GALNT6 |
| FAM89A | CLDN7 |
| PLIN4 | UBE2T |
| NDN | JPT1 |
| ABLIM3 | CDC7 |
| DBX2 | WDR34 |
| IGF1 | CADPS |
| MAB21L1 | MAL2 |

| MME | SUSD4 |
| --- | --- |
| DPT | DIPK1C |
| RGS5 | FCRLA |
| TEX26 | HMGA1 |
| ADCY4 | POC1A |
| CCDC69 | CTHRC1 |
| XIRP1 | EPHB3 |
| SLC1A7 | PARD6B |
| ANGPTL8 | MDK |
| ZNF385D | CENPK |
| HTR4 | RAB11FIP4 |
| ANKRD18B | PLS1 |
| RAB3C | EPPK1 |
| USP44 | ARFGEF3 |
| CXCL14 | SLC38A5 |
| ADAMTS18 | ADAMTS6 |
| CLVS1 | CXCL9 |
| LEPR | FN1 |
| INA | E2F1 |
| APOF | FLNB |
| CCDC3 | CDCP1 |
| PHACTR3 | CELSR2 |
| PAMR1 | TRIB3 |
| LDB2 | NEBL |
| NRN1 | SLC22A12 |
| EDAR | PRR22 |
| CRHBP | CHEK1 |
| STEAP4 | UTS2 |
| BHMT2 | IL4I1 |
| C2CD2 | DPH1 |
| CGB2 | CCNA2 |
| SAA2-SAA4 | HPSE |
| PPARG | CCNB1 |
| EGFLAM | TLCD1 |
| COL6A6 | SULF1 |
| CAT | GALNT3 |
| SYNM | THBS2 |
| NOSTRIN | ACTL10 |
| GABRE | KCNK1 |
| RUNDC3B | KISS1 |
| NPY1R | IRX5 |
| COL9A1 | ITGB8 |
| SLC19A3 | HELLS |
| SCN7A | KIF26B |
| PTPA | MCM10 |
| TLL1 | TCF19 |
| HBEGF | HIST1H4D |
| TMEM132C | FAM72B |
| RELN | CTSD |
| PDZRN4 | DLG3 |
| C7 | SQLE |

| DDR2 | STRC |
| --- | --- |
| SLC16A7 | MATN3 |
| MYO16 | GPSM2 |
| OGN | TYMS |
| HTR1F | MAD2L1 |
| HOXA5 | ECT2 |
| GNAI1 | DNAAF3 |
| HOXA2 | PTTG2 |
| MRAS | TMEM238 |
| TBX15 | RAD54L |
| CYP4X1 | BCAS4 |
| SRPX | CKS2 |
| CCL14 | CLEC5A |
| MESP1 | RIBC2 |
| CYP4B1 | HIST1H2AE |
| IGFBP1 | ABRACL |
| HYAL1 | CD2 |
| EMCN | CTSV |
| LIFR | FNDC10 |
| SPP2 | GPR12 |
| SPTBN1 | ORC1 |
| GYS1 | CAPN1 |
| PTPN5 | SYTL1 |
| NIPSNAP3B | ABCC3 |
| SAA2 | CXCL10 |
| MMRN2 | CORO1A |
| VIP | RAB27B |
| MARCO | RHCE |
| TESC | NBEAL2 |
| DENND2A | EOMES |
| ERG | CFL1 |
| CLDN5 | SHOX |
| AMOTL2 | IL1A |
| FZD4 | ARHGAP11A |
| SMIM10 | NKX3-2 |
| ACACB | FAM72A |
| HMGCLL1 | PLK4 |
| GSTM5 | SKA3 |
| WDR3 | C6orf132 |
| PLPP1 | DYNC1I2 |
| LRRN3 | LY6E |
| RASIP1 | HIST1H2BD |
| C1orf115 | SMCO2 |
| ADGRL4 | TNFRSF9 |
| TSPAN7 | IFITM10 |
| NPR1 | KIF20B |
| SYNE3 | SCG5 |
| ADD1 | TIGD3 |
| CCDC141 | CD8B |
| TMEM246 | CD80 |
| PCDH19 | ADAM8 |

| SELP | RARRES1 |
| --- | --- |
| C1QTNF2 | EPS8L2 |
| PFKFB1 | AURKB |
| ZNF676 | DHRS13 |
| FAM13C | NSG2 |
| REP15 | OCLN |
| USHBP1 | KCNE4 |
| ALX1 | MARVELD2 |
| GSX1 | PRLR |
| FAM149A | PNOC |
| C2orf88 | HIST2H4A |
| SLC35G2 | TRIM59 |
| NDNF | UNC93B1 |
| LAMA2 | WDR90 |
| ENPP2 | ARHGAP39 |
| NKX2-1 | EFNA1 |
| CATSPER2 | TAF7L |
| TCEAL2 | EPHA8 |
| BTBD17 | STARD3 |
| ABCA10 | GRM8 |
| ACSS3 | TMEM171 |
| VLDLR | ADGRB2 |
| AIFM2 | MGAT4B |
| SLCO1B1 | ZYG11A |
| NOVA1 | H1FX |
| NXNL1 | DSC2 |
| AGXT | WWC1 |
| CSRNP3 | NGEF |
| AVPI1 | CD72 |
| MAMDC2 | SERTAD4 |
| GNG11 | FKBP4 |
| ANGPTL3 | CDC6 |
| TBX18 | ALG1L |
| CYYR1 | MACC1 |
| FMOD | RGS4 |
| C10orf90 | CYBA |
| SEMA3A | RASGEF1A |
| TACC1 | CLEC11A |
| VWF | COL12A1 |
| PON1 | MEX3A |
| CABP1 | LILRA4 |
| ADGRF5 | PRR11 |
| PTPRB | SCX |
| TNS1 | TACC3 |
| RNF125 | DEF6 |
| ORM1 | LPAR2 |
| LMOD1 | PPP1R13L |
| TTR | SYCP2 |
| GBP7 | CNKSR1 |
| ABLIM1 | METRN |
| EXOC1L | BARX2 |

| MAP7D3 | STK26 |
| --- | --- |
| PKDCC | DDIAS |
| NEURL1 | MMP9 |
| RGCC | SLC4A2 |
| RUNX1T1 | MIF |
| PAQR9 | TUBB8P12 |
| COBLL1 | DHCR7 |
| CNKSR2 | VANGL1 |
| HCAR1 | GPR84 |
| SOCS2 | ARRDC1 |
| PYGL | EZR |
| SH3D19 | FANCD2 |
| CEP126 | NETO2 |
| NAP1L2 | RCC2 |
| NKX1-1 | NEIL3 |
| TPO | PPP1CA |
| CCDC178 | CLBA1 |
| P2RY12 | CFAP99 |
| TMEM220 | REEP4 |
| NES | XRCC2 |
| TNNC2 | LILRB4 |
| COG8 | CD300LF |
| LECT2 | SMPX |
| PLA2G4A | CENPI |
| DCLK1 | HIST1H4E |
| PDE2A | ZNF695 |
| COLEC11 | PLXNC1 |
| MAPK10 | CXCR4 |
| RELL1 | LRFN3 |
| ID1 | CANT1 |
| CDH12 | FCGR3B |
| DPY19L2 | SMC4 |
| ABCG5 | CDCA8 |
| FAM47E | TREM2 |
| STK32A | DNAH8 |
| GLDN | CENPL |
| PPDPFL | SIGLEC7 |
| PRKAR2B | NME1 |
| CYP7A1 | DPP3 |
| GREM1 | GAPDH |
| ANO3 | SF3B4 |
| RNF157 | PTPRCAP |
| ADRB2 | AGPAT5 |
| PON3 | TLR10 |
| STARD9 | LEF1 |
| CEP112 | CKAP2 |
| RNASE4 | SPAG1 |
| ARID5B | CNIH2 |
| ECM2 | UBE2Z |
| ADCY5 | FAM110A |
| C1QTNF7 | SDC4 |

| KL | BNIPL |
| --- | --- |
| MGLL | IFT140 |
| CNTNAP3 | FANCI |
| HOXA13 | ABHD17C |
| COL19A1 | MAP7 |
| GATA6 | CCL20 |
| CFL2 | HIST2H2BE |
| MMRN1 | KRT36 |
| CDH13 | AGRN |
| TNFRSF11B | MMP10 |
| APOL6 | GJB2 |
| BAAT | RNASE2 |
| EDNRB | SLC29A2 |
| C3orf70 | CORO2A |
| LRRC38 | BEAN1 |
| GOLGA6L22 | KIF3C |
| KCNJ8 | RCAN3 |
| PRXL2A | MCM4 |
| FAT4 | HIST2H2AA3 |
| TAL1 | THOP1 |
| ZIM2 | ANKRD13D |
| CAPN11 | COL5A1 |
| DMD | CDCA7 |
| CTNNAL1 | PAXX |
| FLRT2 | PRRG4 |
| CSPG4 | TFEC |
| PID1 | ITPR3 |
| ADAMTS9 | ARID1A |
| ACVRL1 | PARPBP |
| MYH10 | PIF1 |
| CALCRL | SPATA17 |
| PDE3A | SLC18A3 |
| RNF150 | ERI3 |
| CTTNBP2 | SLC9A3R1 |
| ELANE | CASP2 |
| STOX1 | WDR62 |
| BANK1 | NSD2 |
| RBMS3 | WDHD1 |
| CD34 | WHRN |
| GSN | KRT4 |
| IL4 | AKT1 |
| MARC1 | CTLA4 |
| PLCXD3 | HAGHL |
| PELI3 | POSTN |
| SOD3 | USP21 |
| CLEC14A | TULP1 |
| PLSCR4 | FEN1 |
| GABRB3 | LRGUK |
| THSD7B | DTX2 |
| SCRN2 | NFE2L3 |
| JAM2 | TMED3 |

| NECTIN3 | HIST1H1T |
| --- | --- |
| SYNPO2 | GRK6 |
| PKD1L1 | TPBG |
| TBATA | TMEM121 |
| ADAMTS1 | RPS6KB2 |
| PDE1A | UBASH3B |
| TINAGL1 | FAAH |
| SPRY2 | CATSPER1 |
| RORB | SLAMF7 |
| BTBD16 | HCN3 |
| RRAS2 | SLC4A8 |
| EFEMP1 | IGFL3 |
| SEPT8 | SOWAHD |
| ABI3BP | CENPW |
| NCAM1 | JUP |
| F3 | ARHGAP32 |
| NR3C1 | SPERT |
| SYBU | MACROD1 |
| SCN2B | ZNF710 |
| RFTN2 | ZNF217 |
| DLC1 | TUBAL3 |
| SOX18 | HDHD3 |
| C2CD6 | NGB |
| GRIA3 | KPNA2 |
| ELN | SEC61A2 |
| ST6GALNAC3 | EMILIN1 |
| PTGDS | C19orf47 |
| LRRC34 | TIMM17B |
| NATD1 | FOXD4L1 |
| ELMSAN1 | HAMP |
| ASS1 | C1QB |
| RGS6 | CHTF18 |
| ITM2A | GINS1 |
| FERMT2 | TCF3 |
| ARHGAP6 | SMG5 |
| LAMA4 | SCT |
| TEX48 | AGMAT |
| HIGD1B | ARL4C |
| MYO3A | TELO2 |
| GAGE12F | MND1 |
| PCCA | SLC27A4 |
| COLEC12 | C7orf26 |
| MSX1 | TNFRSF13C |
| PCDH9 | TNFSF4 |
| MYOCD | WNT5A |
| CORO2B | PHLDA2 |
| SHE | GPR141 |
| DKK2 | LCK |
| EMX2 | GAPT |
| INPP1 | GTF3C1 |
| MTRNR2L11 | LIG1 |

| SASH1 | SPINT1 |
| --- | --- |
| AASS | LAT2 |
| STATH | EEFSEC |
| FAXDC2 | MYBL1 |
| HOXA3 | CBFA2T2 |
| HOXA10 | GPR174 |
| CYSLTR1 | TMEM45A |
| SCGN | ITGAX |
| CPA1 | CD3D |
| TFPI | CDC25B |
| PALM2-AKAP2 | HIST1H4J |
| EPN1 | TAF4 |
| RAB6B | EPHX4 |
| NR3C2 | SLC22A18AS |
| TCF7L1 | FLVCR1 |
| PLA2G16 | TRAF7 |
| CPAMD8 | AGTRAP |
| TNXB | ALDH3B1 |
| ICAM2 | TMEM206 |
| KRT25 | TUBA4A |
| ACOT6 | SLC9A7 |
| CRYBG3 | MMS22L |
| ENPP3 | SIGLEC10 |
| CLMP | TUFT1 |
| MTTP | LDLRAD4 |
| PCDHA3 | APOE |
| BHMT | MEN1 |
| PDE9A | PATJ |
| AFAP1L1 | ATP13A2 |
| CLU | ABCC10 |
| AAMDC | ROGDI |
| MAP1B | PHKG2 |
| IL1RAPL1 | FAAH2 |
| SOWAHA | S100A16 |
| MSRB3 | RTKN2 |
| SLC39A12 | TAB1 |
| LYVE1 | GMIP |
| UNC13C | FOXD4 |
| ACAA2 | CENPP |
| FGF7 | APBA2 |
| ADGB | SLC25A40 |
| KANK1 | TRAF3IP3 |
| CREBRF | TRIM28 |
| C10orf82 | CTAGE15 |
| ANKRD20A4 | TMEM87B |
| RHOQ | ACP5 |
| PLVAP | TDRD5 |
| KRT24 | ERP27 |
| COX4I2 | UBXN11 |
| NFIB | PKP3 |
| SPRY4 | CXorf40A |

| SEMA3D | AEBP1 |
| --- | --- |
| SOBP | NT5DC2 |
| MGST1 | EME1 |
| TLCD2 | HIST1H2BE |
| SERPIND1 | STMN1 |
| TRDN | ATAD3A |
| CFAP300 | CHST11 |
| HACD1 | AKT1S1 |
| PC | C15orf48 |
| LRCH2 | HMG20B |
| EPDR1 | C5orf34 |
| KLF9 | CD300A |
| RCAN2 | CCR5 |
| IL11RA | TRAIP |
| LIPF | LAMP3 |
| PROCR | FGD1 |
| FGF14 | RNF19B |
| MYOZ2 | DIAPH3 |
| CDKN1C | C4orf50 |
| RASD1 | ZNF165 |
| PDGFD | ERAL1 |
| NOVA2 | GDI1 |
| MECOM | GPR137C |
| GLI1 | HIST1H2BC |
| THSD7A | CCR8 |
| CAVIN1 | CD177 |
| CRLF2 | KLHL17 |
| THRB | SCNN1B |
| TBPL1 | TTC9 |
| STPG3 | SCML4 |
| FMO3 | CSK |
| NMT2 | SLC12A8 |
| PIWIL2 | LILRA2 |
| HNMT | RBM47 |
| GAS2L2 | MICALL1 |
| KLKB1 | PCDHGA1 |
| GPRASP1 | SIRPD |
| DLG2 | MEIKIN |
| ABCB5 | ORAI1 |
| ESR2 | ENO2 |
| FAM43A | WDFY3 |
| ALDH6A1 | GPR18 |
| IDNK | MCMDC2 |
| PCDHB11 | KMO |
| MAFK | C15orf62 |
| FXYD1 | CARMIL3 |
| GLIPR1L2 | TROAP |
| SAA1 | CHAF1B |
| GPBAR1 | PLXNA3 |
| HRG | CDH11 |
| SCG3 | HIST1H2BM |

| SERPINI1 | SFRP2 |
| --- | --- |
| CLLU1OS | ARHGEF1 |
| TTBK1 | DAPP1 |
| JHY | ZDHHC13 |
| ALDH5A1 | HM13 |
| DHH | SLC44A2 |
| CNTN3 | MED16 |
| C6orf58 | SLC25A39 |
| PGA3 | ALDH3B2 |
| STAT5B | LILRB1 |
| BMX | ATAD3C |
| PECAM1 | CBLN2 |
| HABP4 | DOT1L |
| CFH | UCK2 |
| UTS2B | DRAP1 |
| GIMAP6 | HTT |
| RNF180 | PNPLA6 |
| HOXA6 | LRFN4 |
| TMEM47 | MAN2B1 |
| F8 | SBF1 |
| PPL | SGO1 |
| GOLGA8N | FZR1 |
| CNRIP1 | EVA1A |
| FOXN3 | ADAM30 |
| NEXN | CENPN |
| SLC6A16 | ADAR |
| ALDH1A1 | GPR65 |
| ZNF677 | CD8A |
| KCTD12 | TEX53 |
| TXNIP | KCNQ3 |
| CARD6 | SAMD1 |
| TP53TG3D | PLPP4 |
| TSHZ2 | ARRB2 |
| DUSP6 | B3GNT4 |
| ASB9 | ZDHHC12 |
| ETFB | ENTPD7 |
| AKAIN1 | LMO7 |
| ADM | GRN |
| LIPC | EXPH5 |
| CSF1 | PPAT |
| GUCA2A | UBL4A |
| WDR17 | DCAF15 |
| TLN2 | PCNA |
| ZNF503 | SHANK2 |
| SLC17A3 | DCAF13 |
| MTRNR2L10 | TPRN |
| SELENOP | PATZ1 |
| ARAP3 | SLC39A6 |
| PRR5-ARHGAP8 | PRRC2A |
| FGFBP1 | APOC1 |
| BTNL3 | SAMD12 |

| STEAP1 | CARD11 |
| --- | --- |
| RTP3 | TPBGL |
| EPHX2 | ABCA4 |
| SLC9A9 | TDO2 |
| TRPC6 | LRRC45 |
| CCDC166 | CCSAP |
| SLC25A20 | RTEL1 |
| IL3RA | SDR42E1 |
| NACAD | DNMT3B |
| ATP8B3 | PDPK1 |
| TP53TG5 | EFNA4 |
| GIMAP7 | LAPTM5 |
| MEF2C | CTNND1 |
| LRMDA | ALOX5 |
| GYPE | SEMA4B |
| XKR4 | BORA |
| TRIM48 | MAGEB17 |
| GRK5 | THEMIS2 |
| NPR2 | SH3BGRL3 |
| SCN11A | SPAG4 |
| SLIT2 | PARP12 |
| HIGD1C | ACCSL |
| CCDC152 | P4HTM |
| PRICKLE3 | H2AFY2 |
| RALYL | USP28 |
| BMP6 | MUC5B |
| COPRS | FAM84B |
| C9 | P2RY10 |
| MROH8 | CLEC7A |
| CX3CL1 | RNF31 |
| PABPC5 | CEP250 |
| CCL3 | CIP2A |
| TTC28 | RPGRIP1 |
| FABP4 | CTNNBIP1 |
| PPP2R1B | P2RX5 |
| TSPEAR | R3HDM4 |
| MARC2 | PPP1R37 |
| PDE5A | BSCL2 |
| SPACA9 | PHF12 |
| SCN3A | CERS6 |
| IL1R1 | YWHAE |
| PTH1R | LCE1B |
| THSD1 | FBF1 |
| C8orf88 | CDSN |
| TKTL1 | NCAPH |
| EHD2 | GINS2 |
| NEGR1 | MBOAT2 |
| ENPEP | TRIM11 |
| EHBP1 | ZMYND19 |
| HEY1 | ARHGEF35 |
| FAM92A | CAMK4 |

| BLID | IRF2BP2 |
| --- | --- |
| MAGEB16 | FXYD5 |
| PAPOLB | PTK2B |
| TRARG1 | LAPTM4B |
| TCEAL7 | GRAMD1A |
| PGM5 | DCANP1 |
| POLR3GL | TRAF2 |
| AHSP | IRX3 |
| LRRC70 | PMF1 |
| MFSD2B | SFI1 |
| CSRP2 | CELA1 |
| TEKT4 | ATP6V0C |
| FLYWCH1 | RNF220 |
| ETS2 | MTBP |
| RAB11FIP2 | CD180 |
| ARHGAP24 | RASEF |
| PLPP3 | C1QTNF5 |
| NDRG2 | GGA1 |
| MCTP1 | ZAP70 |
| TRIM68 | ADA2 |
| GDF7 | CENPO |
| MYCT1 | CDC42SE1 |
| HEPHL1 | CDCA2 |
| HOXB3 | TBCB |
| FGL2 | PMAIP1 |
| ALDH1A2 | CCDC9 |
| ARHGAP10 | TSC22D4 |
| CCDC39 | HGH1 |
| ANKAR | SRPRA |
| HADH | TASOR2 |
| WRB-SH3BGR | IPO4 |
| GBA3 | MTMR14 |
| EQTN | HIST1H2BL |
| DENND5B | C2 |
| TTC29 | OSBPL3 |
| MAGI2 | BEST3 |
| PI16 | PCDHGB1 |
| PNMA8C | NR2F6 |
| GCNT4 | LIN9 |
| DEFA1 | MCM5 |
| EEF1A1 | SAMSN1 |
| COL4A3BP | DPEP1 |
| PLA2G2C | PTPRJ |
| BTBD9 | ARMC6 |
| CCDC36 | TSEN54 |
| ARL14EPL | ZBTB7C |
| KCNC3 | KRT17 |
| PHLDB1 | PTBP1 |
| EPS8 | SELL |
| RBM24 | RGS14 |
| ANKRD65 | RUNX1 |

| BEST1 | IL2RB |
| --- | --- |
| GIMAP1 | AP2M1 |
| NFE2 | DBNDD1 |
| ART5 | MYO1F |
| ZNF781 | TPI1 |
| HOXD8 | BRF1 |
| CLDND2 | RDM1 |
| TULP2 | EVI2A |
| ACOT12 | GZMA |
| ANG | PLSCR2 |
| MPDZ | HSF2BP |
| HSDL2 | GAA |
| TNFRSF10D | SF3B3 |
| HSPA1L | MGAT4A |
| MYLK | ABCB9 |
| DHRS11 | AMPD3 |
| NFU1 | PLXNB1 |
| MAML2 | ADCK2 |
| SERPINA7 | TMEM25 |
| SEMA3C | PLA2G15 |
| SWAP70 | FAM111B |
| LDHAL6A | ELOVL1 |
| HIBCH | ATP6AP1 |
| MXRA7 | APEX2 |
| PIP5K1B | RASGRF1 |
| ZFHX4 | QPCT |
| STAT6 | PYCR1 |
| FGF13 | TMEM176A |
| PRR18 | SLC38A1 |
| LETMD1 | RHOC |
| SSPN | GGA3 |
| ETFDH | VAMP8 |
| APOL3 | ALCAM |
| MXI1 | SEMA4A |
| PZP | SLC16A3 |
| TK2 | SLA |
| WDR93 | ELMO3 |
| BEND5 | TSPAN33 |
| HOXA7 | MESP2 |
| CLYBL | LRRC61 |
| DHFR | TMEM184A |
| HLF | FUT7 |
| AOC3 | HIST1H2BJ |
| RAB44 | OCLM |
| RIMBP2 | DCAF10 |
| ACTN3 | TIGIT |
| HOXC12 | IKZF3 |
| NR1H3 | CCDC86 |
| GPATCH11 | NOL4L |
| HOXB4 | SLC35E1 |
| TRIM49B | CENPH |

| C1GALT1C1L | AXDND1 |
| --- | --- |
| MOGAT3 | CARM1 |
| SNCA | ITGA2B |
| MYO15B | SUCO |
| LRTM1 | EGLN3 |
| MAP2K6 | ZBTB8A |
| SLCO1B3 | PFKFB4 |
| PELI1 | HIST1H2AC |
| PARD3B | FAM234A |
| CCDC144A | NME1-NME2 |
| SCO1 | CACNG1 |
| GNAL | CEBPE |
| STX2 | PUS7 |
| CCT6B | TBC1D3B |
| OR2T12 | NUP62 |
| STARD13 | TMIGD2 |
| ZNF660 | LY86 |
| ROM1 | STIP1 |
| FAM228B | SEC61A1 |
| PDLIM1 | SRGAP2 |
| FER | AP3D1 |
| SNTB1 | FSD1L |
| VCL | TSTA3 |
| CC2D2A | B4GALT2 |
| ARHGEF28 | NAPA |
| PTEN | CALHM6 |
| RGS17 | BAIAP2L1 |
| KRBA2 | EXOSC4 |
| HMGN5 | RAD54B |
| B4GAT1 | BANP |
| GPR62 | AMH |
| ALDH2 | CD226 |
| PRKN | FUK |
| TMEM74B | WDR6 |
| RHOU | TEX45 |
| CCDC50 | CNNM4 |
| JADE2 | XAB2 |
| DHRS7B | CBX4 |
| BORCS8-MEF2B | TMEM40 |
| CTXND1 | APOBR |
| LAMC1 | MRGBP |
| SH2D3C | ZNF581 |
| NEDD9 | CIAO3 |
| MSRA | FYB1 |
| BFSP1 | CD48 |
| FAM217A | ODF2 |
| SYNC | 9-Mar |
| BCORL1 | RFWD3 |
| ATRX | SOX4 |
| S100A12 | POLRMT |
| KANK3 | SLC3A2 |

| L3HYPDH | GFER |
| --- | --- |
| PCDHB3 | RMI2 |
| EPAS1 | BRMS1 |
| ADI1 | CAVIN4 |
| CLEC1A | PRICKLE1 |
| CHURC1 | ZWILCH |
| EID1 | ETV7 |
| PJVK | USP31 |
| FEV | DNASE1L2 |
| ANGPTL2 | HAUS5 |
| PCDH18 | PODXL2 |
| RMDN2 | G6PD |
| JAM3 | C9orf16 |
| CYP2U1 | LINGO3 |
| IGIP | ETNK1 |
| PKIG | MTFP1 |
| CAMKK1 | CACNB3 |
| CLIC2 | BHLHE41 |
| UFSP2 | SIX4 |
| NKX6-2 | CDK16 |
| DAAM2 | LEMD2 |
| MAST3 | ATP6V0D2 |
| PBLD | CD101 |
| ANXA1 | PACS2 |
| GRID1 | TEX11 |
| DUSP22 | CEP97 |
| PYGO1 | TEDC2 |
| SGSM1 | PASK |
| NKAPL | NDRG3 |
| RASL10B | BICDL1 |
| PTGR2 | RNF24 |
| CHRNB2 | SMYD5 |
| PTGER1 | LRWD1 |
| CCL15 | PLD3 |
| RNLS | CCL5 |
| ALS2CR12 | NIPSNAP1 |
| GAB2 | INCENP |
| OR52N4 | METTL11B |
| MKLN1 | GPRC5A |
| ING5 | RFC4 |
| ZFYVE21 | CENPX |
| CDH6 | TMEM141 |
| DPPA3 | TIAM1 |
| GCSAML | MLF2 |
| ESD | RIMS3 |
| SERPINA4 | MGME1 |
| BCKDHB | NINJ1 |
| CTH | PRKDC |
| LMX1A | COL27A1 |
| MAT2A | KDM2A |
| ZNF501 | SFXN1 |

| FRY | PLEKHN1 |
| --- | --- |
| FBXO8 | STAG3 |
| HUWE1 | NEFH |
| UBE2Q2L | VPS13B |
| KCTD8 | PPT1 |
| TSPAN31 | OR2B2 |
| RBPJ | E2F4 |
| ADAMTSL4 | TMEM51 |
| STEAP1B | RHNO1 |
| LRRC71 | BRPF1 |
| BPIFB2 | ERO1A |
| EPHA2 | BARD1 |
| LCAT | GBA2 |
| THPO | ADAM12 |
| ZIC5 | TIMELESS |
| CALHM4 | KLHL35 |
| HOXB8 | FAM102A |
| ACADSB | DCTPP1 |
| SNTB2 | SMIM29 |
| APCDD1 | E2F2 |
| TSLP | DENND1B |
| GULP1 | ANP32E |
| WHAMM | SLC30A2 |
| CYB5D2 | POGK |
| CASC3 | UBQLN4 |
| ADAM22 | LAGE3 |
| UBN2 | HIST1H2BN |
| TTC7B | SLC2A4RG |
| TNS2 | FLAD1 |
| MYCBP2 | RAI14 |
| SHH | OGG1 |
| TMEM273 | IKBKE |
| MCOLN3 | CD9 |
| SESTD1 | INPPL1 |
| ZNF679 | FGFR1OP |
| SLC25A47 | PRKCSH |
| TMIE | SLC35A3 |
| TEX12 | GRK2 |
| PAK4 | KRTAP2-2 |
| GRK1 | FAM174B |
| PINX1 | C19orf57 |
| DENND4C | KLK4 |
| DOK1 | NOL6 |
| OR5K2 | ATAD3B |
| OR2A2 | AMPD2 |
| UGT2B10 | RANGAP1 |
| C21orf62 | PDIK1L |
| REM1 | NRBP1 |
| TDRD6 | DUS1L |
| LCA5 | BCL2L12 |
| TEX43 | TLE6 |

| UGT2A3 | FIGNL1 |
| --- | --- |
| UGT1A9 | TIPRL |
| PIK3C2B | ERGIC3 |
| ACCS | MREG |
| MAF | LRRC59 |
| FMN2 | SLC25A19 |
| WBP4 | TMED9 |
| MYL1 | THOC3 |
| KAT2B | HENMT1 |
| ZNF391 | MCM3 |
| CBLB | TMEM8A |
| SMYD4 | PLA2G2D |
| OSBPL1A | MINK1 |
| ECHDC2 | YIPF3 |
| CHCHD10 | SMPD4 |
| ABHD12B | AP2S1 |
| DEFB123 | LYG1 |
| AGPAT2 | PSMC4 |
| PRKAA1 | CLPSL1 |
| PHLDA1 | SP9 |
| CYBRD1 | SLA2 |
| SEMA6D | CERS4 |
| WSB1 | TMEM63A |
| ACAT1 | OSBPL5 |
| SBDS | P2RY2 |
| CDNF | ARL11 |
| HECW2 | SLC16A6 |
| EPHA3 | IL18 |
| RNF165 | SMC2 |
| KALRN | CNDP2 |
| ZFP28 | SEMA7A |
| FLT1 | RASAL1 |
| UQCRC2 | PANO1 |
| TAOK1 | KIAA1522 |
| TACC2 | TRRAP |
| SH3KBP1 | SLC7A7 |
| SRR | LRRC15 |
| MBNL2 | DGKA |
| MEI1 | CLSTN1 |
| C9orf3 | CDYL2 |
| ADRA1A | FOXK1 |
| CCDC82 | PRCC |
| RPGR | SIRPB1 |
| TMBIM4 | KDM5B |
| PCDHAC2 | OR2J3 |
| NUBPL | PCDHGB5 |
| TNFSF14 | GIP |
| EPHA4 | BMP2K |
| TP53I3 | GZMM |
| GNL1 | SMC1A |
| COX14 | APOBEC3B |

| AXIN2 | ITGAD |
| --- | --- |
| TUSC1 | OCIAD2 |
| C14orf28 | FMNL1 |
| GALT | CNPY3 |
| SEPT4 | UBAP2 |
| VTI1B | ASNA1 |
| APOC3 | HK1 |
| GNPDA2 | CHMP6 |
| SH3BP5 | POP1 |
| ZCWPW2 | AOAH |
| NPIPB8 | SRRT |
| ANKMY2 | SETD1A |
| UBA2 | SGO2 |
| ARG1 | BOP1 |
| LIMA1 | ABCC5 |
| RXYLT1 | PICK1 |
| WDPCP | NCAPH2 |
| ADGRG7 | ZNF541 |
| TARSL2 | RHBDD3 |
| CPQ | FAM219A |
| C12orf40 | CEP72 |
| IFNA8 | NCCRP1 |
| OLFML1 | RNF40 |
| C17orf58 | CD53 |
| JMY | PRKAR1B |
| LRRC32 | MAP3K9 |
| MECP2 | SGPL1 |
| SYNGR4 | CRKL |
| ITSN2 | E4F1 |
| NLGN4X | MXRA5 |
| METTL26 | POU2F2 |
| HYKK | ZNF469 |
| BECN1 | CAPN15 |
| CCDC121 | ABCF3 |
| STXBP1 | LRP8 |
| ZNF888 | RNPEP |
| ZDBF2 | KNTC1 |
| APOA5 | RAB11FIP3 |
| NKIRAS1 | CD300C |
| IMMP2L | KIAA2013 |
| CPTP | SLCO2B1 |
| GPR6 | EPSTI1 |
| USP12 | DDX28 |
| ZFP91 | LRIF1 |
| MGMT | ASCC3 |
| CCM2L | C6orf52 |
| BCL6B | HEATR3 |
| ATG10 | CST7 |
| FOXH1 | PTPN22 |
| USP40 | WDR18 |
| GTF2H2C | TOR3A |

| GALNT16 | RCC1 |
| --- | --- |
| OPN1LW | UNC13D |
| ARSK | THY1 |
| RPA4 | SLC39A3 |
| MYZAP | CDK2AP2 |
| TMLHE | SIPA1L3 |
| TNFRSF21 | NAT9 |
| OR1E1 | MRPS34 |
| TSTD3 | VGF |
| DNAH6 | MSR1 |
| BOK | TMEM30B |
| AKT3 | AP1M1 |
| TCF4 | MIER2 |
| TMEM192 | P2RX7 |
| OVOL1 | COL1A1 |
| SPARCL1 | BRCA2 |
| SLC29A1 | LRRC3C |
| MYF6 | ARHGDIB |
| KBTBD3 | F2RL1 |
| BORCS7 | KDM5C |
| SPNS3 | RAD21 |
| EBF2 | ZNF653 |
| MCEMP1 | NOD2 |
| ATP1B2 | HDAC1 |
|  | DEFB131A |
|  | IGSF6 |
|  | PI4KB |
|  | RRP1 |
|  | TMEM191B |
|  | COL1A2 |
|  | GTPBP3 |
|  | PDPN |
|  | TUBB8 |
|  | RNF5 |
|  | ZNF146 |
|  | METTL27 |
|  | URB2 |
|  | AP2A1 |
|  | C1QTNF6 |
|  | COL5A2 |
|  | GRHL2 |
|  | GIGYF1 |
|  | PTTG1IP |
|  | MMP2 |
|  | DOP1B |
|  | NIT2 |
|  | PKMYT1 |
|  | PI4KA |
|  | ARSE |
|  | MRPL4 |
|  | USH1G |

| GPS1 |
| --- |
| ACAP1 |
| ZNRF2 |
| LSR |
| FAM131A |
| RPS6KA1 |
| NKG7 |
| KNSTRN |
| GEN1 |
| PCDHGA3 |
| IPO13 |
| PUSL1 |
| C1QC |
| UCN |
| CSNK1D |
| LETM2 |
| EMD |
| DSN1 |
| GSS |
| TRAPPC1 |
| SLC10A5 |
| PLP2 |
| HSH2D |
| UBE2M |
| EVL |
| PCDHGA4 |
| B4GALT3 |
| BAIAP2 |
| GALNT2 |
| RPS26 |
| CIDEB |
| MAD1L1 |
| RALY |
| NHP2 |
| APOBEC3F |
| SETD5 |
| GPRIN1 |
| CAMSAP1 |
| CEMIP |
| ARHGDIG |
| CD74 |
| AKAP8L |
| UTP18 |
| CSMD3 |
| SLC7A1 |
| NFKBIE |
| DTYMK |
| POP7 |
| FAM171A2 |
| GRAMD1B |
| NUP188 |

| MTA1 |
| --- |
| CD86 |
| RGS19 |
| CAPZB |
| TSEN34 |
| BLM |
| MORF4L2 |
| HPRT1 |
| IL37 |
| MYO19 |
| CHRNA6 |
| RNF32 |
| TRABD |
| SH3D21 |
| LIMK2 |
| HOPX |
| MYBPH |
| CLTC |
| EDEM1 |
| NME4 |
| SETDB1 |
| LMNB2 |
| ARF1 |
| SMG9 |
| POLR3G |
| SPRY3 |
| ARHGAP27 |
| SPDYE2 |
| TMEM102 |
| CALR |
| MOCOS |
| MRPL14 |
| CEACAM18 |
| GTF2IRD1 |
| KDELR1 |
| FAM207A |
| RAB3IP |
| SH3PXD2B |
| BLK |
| DIRC1 |
| RAD1 |
| ILF3 |
| NUP155 |
| PCDHGA6 |
| KCTD5 |
| FAM98C |
| TPGS1 |
| ZNF593 |
| PPP1R11 |
| GOLT1A |
| SMIM18 |

| APRT |
| --- |
| TNFAIP1 |
| TMEM52 |
| CPNE1 |
| IDH2 |
| ARHGAP22 |
| CLPTM1L |
| SLX4 |
| CD3EAP |
| NAGK |
| DEGS2 |
| LRSAM1 |
| NCF1 |
| NR2C2AP |
| SIGLEC5 |
| BCL7C |
| SRPK1 |
| SH3BP2 |
| APLP2 |
| PUS1 |
| SAMD3 |
| MCOLN1 |
| TGIF1 |
| HSD11B1L |
| KRTAP5-4 |
| BEND6 |
| GNL3L |
| CDCA7L |
| CACTIN |
| PTPRD |
| ZNF443 |
| ABCB8 |
| PDAP1 |
| KCNK15 |
| SIPA1 |
| MNDA |
| PPP1R14B |
| MIA2 |
| UBAC2 |
| CNOT6 |
| CDC42BPA |
| RMND5B |
| CHPF |
| 1-Mar |
| CLN6 |
| RANBP1 |
| SREBF2 |
| HYAL3 |
| NDE1 |
| STAP1 |
| WDR4 |

| MAGEB3 |
| --- |
| SMG8 |
| C7orf50 |
| FRRS1 |
| AIFM3 |
| DYRK1A |
| ANGEL1 |
| MAP2K2 |
| SLC15A3 |
| TMEM9 |
| PLXDC1 |
| SRSF2 |
| HNF4A |
| GPRC5D |
| CHD3 |
| PYCR3 |
| USP22 |
| UBA1 |
| MCM6 |
| LTBR |
| CHEK2 |
| GDF15 |
| NCOA5 |
| ZMIZ1 |
| NXN |
| BCL2L1 |
| REPIN1 |
| SPC24 |
| ASB6 |
| MOB1A |
| RFPL3 |
| FRAT2 |
| RAB4B |
| RAB3D |
| TCP10L2 |
| GET4 |
| TESK2 |
| NRTN |
| GMPPA |
| TECTB |
| SLC2A9 |
| H1F0 |
| SEC23B |
| CDK4 |
| PCDHGA12 |
| SIGMAR1 |
| PTPN3 |
| PPM1J |
| SLC35E2B |
| PEX7 |
| CTPS2 |

| PPIH |
| --- |
| DOHH |
| USF2 |
| ST3GAL4 |
| NSUN5 |
| KATNB1 |
| ZDHHC5 |
| DRD4 |
| CLSPN |
| ABCA2 |
| AZIN1 |
| TAS2R1 |
| LUM |
| MTHFD2 |
| CAPNS1 |
| CXXC1 |
| HIST1H2AG |
| MOXD1 |
| GNB2 |
| NCDN |
| POLR2J |
| HSPA2 |
| SLC4A3 |
| IDUA |
| DCAF11 |
| SLC35B1 |
| GALE |
| PSMG3 |
| SLC12A3 |
| KDM4B |
| PITPNM1 |
| TRANK1 |
| SDF2L1 |
| FAM189B |
| AHRR |
| CCDC51 |
| WRNIP1 |
| GPR108 |
| QSOX2 |
| NRL |
| RFC3 |
| TTC26 |
| ZNF28 |
| KHDC4 |
| POLR2K |
| B4GALT1 |
| ARHGEF5 |
| NUP93 |
| TIGD5 |
| FADS2 |
| BCKDK |

| NUDCD1 |
| --- |
| RFC2 |
| GTPBP1 |
| BANF1 |
| ARHGAP4 |
| FANCF |
| PROSER3 |
| DR1 |
| JOSD2 |
| PWWP2B |
| RIMKLA |
| BFSP2 |
| HHLA3 |
| HSPA14 |
| SMAD1 |
| SCIMP |
| MAEL |
| MSH2 |
| FCMR |
| ABI2 |
| SULF2 |
| DPH7 |
| ACIN1 |
| KIF21B |
| WEE1 |
| RGS10 |
| PRR4 |
| EVPL |
| NME2 |
| VGLL4 |
| APOC4-APOC2 |
| GOLGA4 |
| SF3A2 |
| GID8 |
| RCCD1 |
| YRDC |
| KNOP1 |
| ARMC12 |
| TCIRG1 |
| YEATS4 |
| PHF24 |
| RRH |
| FARSA |
| SNRPB |
| C2CD2L |
| PRPS2 |
| HACD3 |
| USP5 |
| IPCEF1 |
| CLPP |
| NDOR1 |

| ISG15 |
| --- |
| TBL2 |
| PBOV1 |
| TUBA1C |
| FKTN |
| ANKRD11 |
| SDC1 |
| RNF2 |
| TREML1 |
| TTF2 |
| GPATCH2 |
| MCM9 |
| CBX3 |
| ZNF358 |
| NME3 |
| LARP1 |
| STRIP2 |
| GMPPB |
| PDSS1 |
| GM2A |
| TMTC4 |
| GCHFR |
| CPT1A |
| FAM136A |
| OR2A4 |
| U2AF2 |
| MMEL1 |
| CCDC182 |
| RNF38 |
| RAB42 |
| SFMBT1 |
| RGS12 |
| ENC1 |
| SEL1L3 |
| PLCXD2 |
| REPS1 |
| UTS2R |
| SPATA13 |
| LRRK1 |
| SNRPE |
| TGFBRAP1 |
| MAGOHB |
| ACTL6A |
| ELFN2 |
| GTPBP2 |
| SLC4A7 |
| SLC2A8 |
| DNAJC5 |
| NLN |
| RAB10 |
| KCTD20 |

| MYO1G |
| --- |
| SMC5 |
| MLLT10 |
| GGA2 |
| WDR1 |
| TRIM14 |
| SIMC1 |
| C20orf96 |
| EIF2AK1 |
| EPHA1 |
| CXorf21 |
| C8G |
| NFRKB |
| PPP6R2 |
| CD276 |
| LEPROTL1 |
| CLEC16A |
| PLEKHB2 |
| PYGO2 |
| NRG2 |
| UBASH3A |
| PARVG |
